# Gold(i) and gold(iii) carbene complexes from the marine betaine norzooanemonin: inhibition of thioredoxin reductase, antiproliferative and antimicrobial activity†

**DOI:** 10.1039/d4md00358f

**Published:** 2024-07-31

**Authors:** Seyedeh Mahbobeh Mahdavi, Dirk Bockfeld, Igor V. Esarev, Petra Lippmann, René Frank, Mark Brönstrup, Ingo Ott, Matthias Tamm

**Affiliations:** a Institut für Anorganische und Analytische Chemie, Technische Universität Braunschweig Hagenring30 38106 Braunschweig Germany m.tamm@tu-bs.de; b Institute of Medicinal and Pharmaceutical Chemistry, Technische Universität Braunschweig Beethovenstraße 55 38106 Braunschweig Germany; c Department of Chemical Biology, Helmholtz Centre for Infection Research GmbH Inhoffenstrasse 7 38124 Braunschweig Germany

## Abstract

The natural marine betaine norzooanemonin (1,3-dimethylimidazolim-4-carboxylate) and its methyl and ethyl esters were used as ligand precursors to prepare a systematic series (12 members) of neutral monocarbene gold(i/iii) and cationic dicarbene gold(i/iii) complexes. The complexes were evaluated as inhibitors of bacterial thioredoxin reductase and for their antiproliferative and antimicrobial activities. While gold complexes with the parent norzooanemonin scaffold resulted in overall poor performance, the more lipophilic esters proved to be highly bioactive agents, related to their higher cellular uptake. The monocarbene gold(i/iii) complexes showed significant potency as inhibitors of bacterial thioredoxin reductase. In most assays, the efficacy of both gold(i) and gold(iii) analogues was found to be comparable. The cytotoxicity of dicarbene gold(i/iii) complexes against cancer cells was strong, in some cases exceeding that of the standard reference auranofin.

## Abstract 2

Gold(i/iii) complexes based on the marine natural betaine norzooanemonin display excellent antibacterial and cyctotoxic activity based on the nature of carboxylate functionalization.

## Graphical abstract

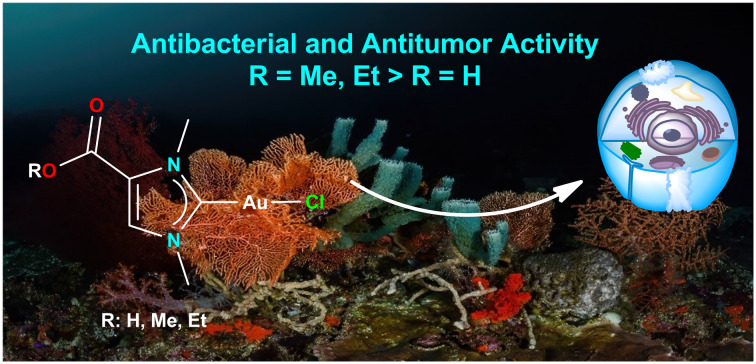

## Introduction

Currently, drug-resistant bacteria cause 700 000 casualties worldwide each year, and the expected increase to up to 10 million incidences by the year 2050 clearly underpins the urgent need for new and improved antibiotics.^1^ Progress in antibiotic agent development strongly relies on the identification of new target structures for the bacterial inhibition. However, the majority of commercialized antibiotics are based on very few chemical motifs, among which prominent examples include penicillins or tetracyclines. Metal complexes offer a wide range of unexplored chemical scaffolds for antibacterial drug design.^2^ Gold complexes in particular have attracted considerable attention in medicinal chemistry since early reports by Robert Koch on the antimicrobial activity of gold cyanido complexes against tuberculosis^3^ and stimulated by the well-known gold drug auranofin with anti-inflammatory activity for the treatment of rheumatoid arthritis. The antibacterial activity of gold complexes is now well documented,^4^ and in this context, an important mechanism is the inhibition of thioredoxin reductase (TrxR). Notably, many Gram-positive pathogenic bacteria lack sufficient levels of glutathione, thus making their metabolism highly dependent on the activity of TrxR, which reduces oxidised thioredoxin (Trx). As a result, the Trx/TrxR system controls the growth of bacteria with low glutathione levels, which can be perturbed by the interference with gold complexes.^5^ The biological role of gold complexes reaches far beyond antibacterial activity, as many gold complexes additionally display excellent antitumour activity.^6^ This is partly due to the fact that some TrxR variants are overexpressed in cancer cells, but are also present in many Gram-positive bacteria. Inhibition of this enzyme may therefore be effective against cancer as well as several infectious diseases, including leishmania, malaria and trypanosomes.^7,8^ In particular, gold complexes with *N*-heterocyclic carbene ligands (NHC) have captured high interest.^8,9^*N*-Heterocyclic carbenes are strong σ-donating ligands, which form robust gold–carbon bonds with exceptionally high hydrolytic stability under physiological conditions.^10^ The synthesis and characterisation of *N*-heterocyclic carbene gold complexes has stimulated their applications in medicine, optics, and catalytic processes.^11^ Highly stable structural motifs include monocarbene complexes of the type (NHC)AuX and cationic dicarbene complexes of the type [(NHC)_2_Au]X (X = anionic ligand). Recent structure–activity-relationship (SAR) studies manifest that (i) monocarbene gold complexes demonstrate significantly higher inhibition of bacterial TrxR and antibacterial activity compared to cationic dicarbene gold complexes, (ii) the dicarbene gold complexes show notably greater antiproliferative activity against cancer cell lines, and (iii) NHC–gold(i) complexes have similar activity compared to NHC–gold(iii) complexes.^12–15^

**Scheme 1:**
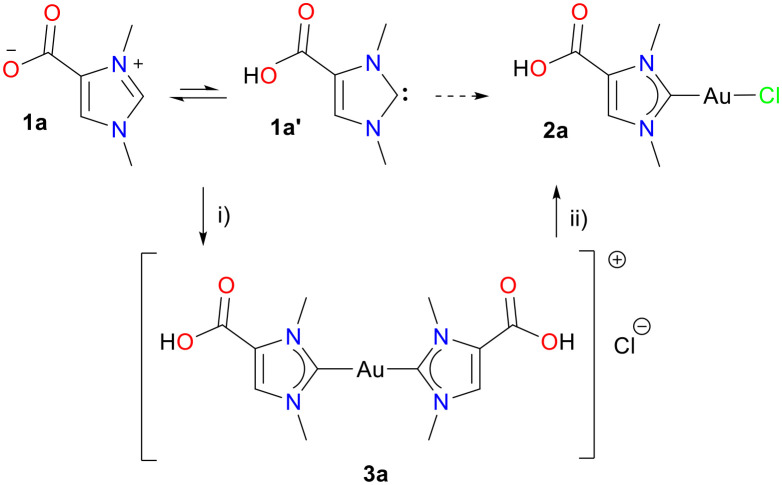
Preparation of mono and dicarbene gold(i) complexes 2a and 3a from norzooanemonin. Reagents and conditions: i) 1 eq. K_2_CO_3_, 0.5 eq. (Me_2_S)AuCl, MeOH, 12 h, rt; then HCl (2 M), pH = 1–2, H_2_O, rt; ii) 1 eq. (Me_2_S)AuCl, MeOH, 4 h, rt.

Among the wide range of NHC ligands, the marine natural product norzooanemonin (1,3-dimethylimidazolium-4-carboxylate, 1) has attracted our interest (Scheme 1). It was isolated from *Pseudopterogorgia americana* (a Caribbean gorgonian) in 1973 and gives rise to tautomers 1a (betaine form, major) and 1a′ (carbene form, minor) Scheme 1.^16^ In the form of 1a′, it can be considered as a member of the smallest NHCs with a carboxyl group, offering routes for chemical functionalisation. Only recently, our group demonstrated the synthesis of gold(i) complexes 2a and 3a from norzooanemonin for the first time.^17^ The synthesis of the monocarbene complex 2a proved to be challenging, as equimolar amounts of 1a and (Me_2_S)AuCl did not yield the desired complex in satisfactory purity. In contrast, the dicarbene complex 3a was readily isolated by reaction with two equivalents of 1a after acidification. The subsequent reaction of 3a with another equivalent of (Me_2_S)AuCl afforded complex 2a. Both mono- and dicarbene gold(i) complexes are of interest for medicinal applications, and 2a and 3a were obtained in analytically pure form. The carboxylic acid complexes 2a and 3a are soluble in polar solvents, but showed reduced cytotoxicity against cancer and human cells, weak activity against Gram-negative and Gram-positive strains as well as poor inhibition of thioredoxin reductase in bacteria. These properties have been attributed to the high polarity of 2a and 3a, which may impede cellular uptake. With this working hypothesis, we decided to increase the lipophilicity by esterification at the carboxyl group, which would thus improve cellular uptake.

## Results and discussion

### Synthesis and characterisation of NHC–gold complexes

**Scheme 2:**
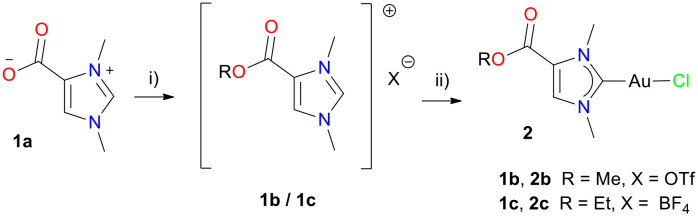
Preparation of monocarbene gold(i) complexes 2b and 2c from norzooanemonin. Reagents and reaction conditions: i) 1b: 1 eq. methyl trifluoromethanesulfonate, DCM, 1 h, rt; 1c: 1 eq. triethyloxonium tetrafluoroborate, DCM, 4 h, rt; ii) 1 eq. K_2_CO_3_, MeOH (for 2b) or EtOH (for 2c), then 1 eq. (Me_2_S)AuCl, 12 h, rt.

Norzooanemonin (1a) was prepared from 1-methylimidazole and dimethyl carbonate as previously reported.^17^ The reaction of 1a with a stoichiometric amount of methyl triflate afforded the methyl ester 1b as a white solid in 86% yield (Scheme 2). Similarly, the ethyl ester 1c was obtained with triethyloxonium tetrafluoroborate as a white solid in 51% yield (Scheme 2). The ^1^H NMR spectra show signals, which unambiguously indicate the introduction of ester moieties. Thus, among the expected resonances of the parent norzooanemonin scaffold, a signal at 3.84 ppm in 1b indicates the methyl ester, while peaks at 4.37 and 1.36 ppm were attributed to the ethyl group in 1c. Further confirmation was provided by the ^13^C{^1^H} NMR spectrum, in which the carboxyl groups resonate at 157.6 ppm and 157.3 ppm for 1b and 1c, respectively. X-ray crystallographic analysis clearly established the identity of 1b and 1c (Tables S1 and S2†), which we considered to be suitable precursors for more lipophilic NHC gold complexes with the norzooanemonin scaffold. Thus, a mixture of ester 1b or 1c with potassium carbonate was stirred in methanol (1b) or ethanol (1c), and the subsequent addition of (Me_2_S)AuCl afforded complexes 2b or 2c as white powders in moderate yields of 50% for 2b and 46% for 2c (Scheme 2).

**Fig. 1:**
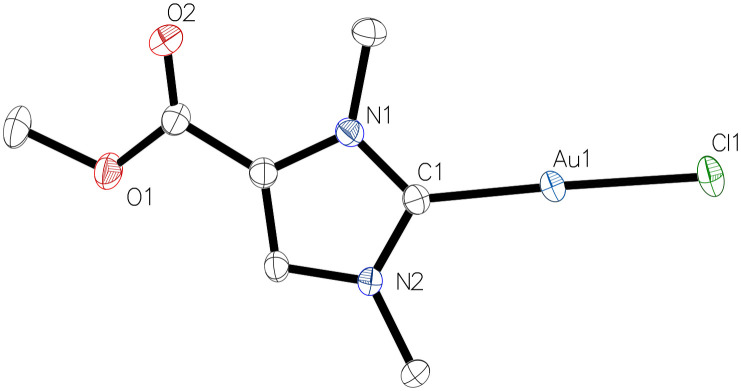
Molecular structure of monocarbene gold(i) complex 2b with one of the two independent molecules.

The ^1^H NMR spectra showed the expected signals for complexes 2b and 2c. In particular, the characteristic H-2 proton signals of the imidazolium heterocycle in the starting materials 1b (9.00 ppm) or 1c (8.84 ppm) were absent, demonstrating the deprotonation and complexation event to give 2b and 2c. The ^13^C{^1^H} NMR spectra display low-field signals at 176.7 ppm (2b) and 176.6 ppm (2c), which are diagnostic for the carbene carbon atom C-2 in gold(i) NHC complexes.^14,17^ In addition, resonances at 158.7 ppm (for 2b) and 158.3 ppm (for 2c) indicate the carboxyl groups. The identity of complexes 2b (Fig. 1 and Table S3†) and 2c (Table S4†) was confirmed by X-ray crystallography. The complexes crystallised in the monoclinic space groups *P*2_1_/*n* (2b) or *P*2_1_/*c* (2c) with two independent units each, the metrical parameters of which are identical within the crystallographic accuracy (Tables S3 and S4†). The angles C1–Au1–Cl1 measured in one of the independent molecules are consistent with the expected nearly linear two-coordinate environment at the gold atom, *i.e.* 178.05(13)° for 2b and 178.42(6)° for 2c. Moreover, the Au1–C1 and Au1–Cl1 bond lengths amount to 1.987(2) Å and 2.2844(6) Å for 2b, and 1.984(2) Å and 2.2873(5) Å for 2c, and are in line with the previously reported data for 2a.^17^

**Scheme 3:**
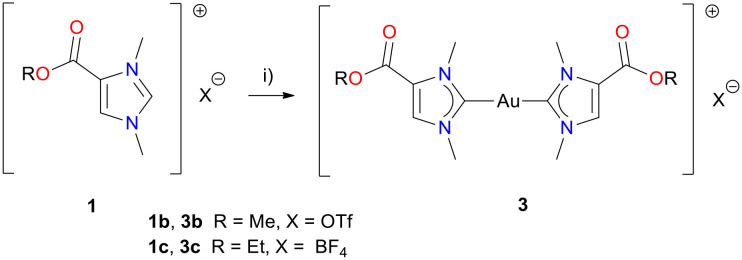
Preparation of dicarbene gold(i) complexes 3b and 3c. Reagents and reaction conditions: i) 1 eq. K_2_CO_3_, 1 eq. (Me_2_S)AuCl, acetone, 4–7 days, rt.

For the synthesis of the cationic dicarbene complexes 3b or 3c, a mixture of 1b or 1c, potassium carbonate and (Me_2_S)AuCl was stirred in acetone (Scheme 3). Purification by column chromatography afforded complexes 3b or 3c as white powders in yields of 66% for 3b and 54% for 3c, respectively. Again the ^1^H and ^13^C{^1^H} NMR spectra of 3b and 3c are consistent with their formulation as dicarbene gold(i) complexes. In comparison to the monocarbene gold(i) complexes (2b, 2c), the ^1^H NMR resonances of 3b and 3c are slightly low-field shifted (*ca.* 0.15 ppm), in accordance with their cationic nature. This trend is even more pronounced for the gold-coordinated carbene atoms C-2 in the ^13^C{^1^H} NMR spectra, *i.e.* 176.7 ppm (2b) *vs.* 188.5 ppm (3b) and 176.6 ppm (2c) *vs.* 188.1 ppm (3c), as previously observed for similar complexes.^18–20^ Furthermore, the ^13^C{^1^H} NMR signals indicated the ester (CO) groups at 159.2 ppm (3b) and 158.5 ppm (3c).

**Fig. 2:**
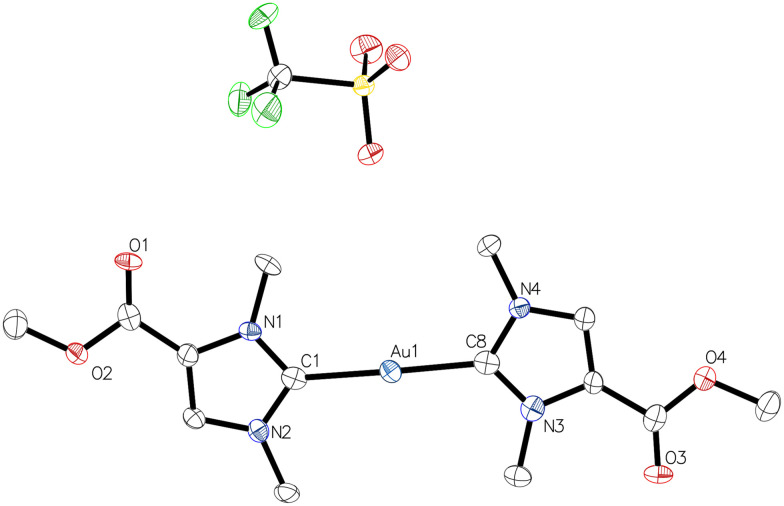
Molecular structure of dicarbene gold(i) complex 3b with one of the two independent units.

The identity of 3b (Fig. 2 and Table S5†) and 3c (Table S6†) was determined by single crystal diffraction. Complex 3b crystallized in the monoclinic space group *P*2_1_/*c* with two independent entities per unit cell, the metrical parameters of which are identical within the crystallographic errors. Complex 3c was obtained in the triclinic space group *P*1̄ with one independent unit. Although the crystallographic data for 3c are of limited quality, the connectivity within this complex is confident, but we prefer to omit discussion of the metrical parameters in 3c. For 3b, the C1–Au–C8 angle in one of the independent units is 178.7(4)°, consistent with the expected linear geometry, and the bond lengths Au1–C1 2.014(9) Å and Au1–C8 2.015(8) Å can be considered as equal.

**Scheme 4:**
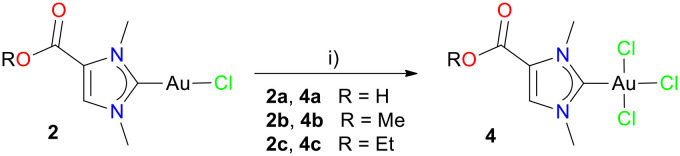
Preparation of monocarbene gold(iii) complexes 4a–c from monocarbene gold(i) complexes 2a–c. Reagents and reaction conditions: i) 1.4 eq. PhICl_2_, DCM or DCM/MeOH, 24 h, rt.

Besides NHC–gold(i) complexes, analogous NHC–gold(iii) complexes are also usually considered as pharmacologically active agents (*vide supra*). Therefore, we set out to prepare such complexes employing the norzooanemonin scaffolds in 1a–c. Thus, with gold(i) complexes 2a–c in hand, the oxidation with iodobenzene dichloride (PhICl_2_) afforded complexes 4a–c as white powders in high yields of 85%, 87% and 89%, respectively (Scheme 4). The ^1^H NMR spectra of the monocarbene gold(iii) complexes 4a–c show slight low-field shifts (up to 0.1 ppm) compared to the corresponding monocarbene gold(i) compounds 2a–c, consistent with the higher oxidation state of the metal centre. In addition, the proton signal for the carboxyl group in 4a is diagnostically exchanged in CD_3_OD solution. In contrast to the ^1^H NMR, the ^13^C{^1^H} NMR spectra of 4a–c show a pronounced high-field shift for the distinct carbene C-2 atoms compared to 2a–c, *i.e.* 176.7 ppm (2a) *vs.* 146.4 ppm (4a), 176.7 (2b) *vs.* 148.9 ppm (4b), and 176.6 (2c) *vs.* 148.3 ppm (4c).^15^ Furthermore, the ^13^C{^1^H} NMR shows signals for the carbonyl groups at 160.2 ppm (4a), 157.7 ppm (4b) and 157.2 ppm (4c).

**Fig. 3:**
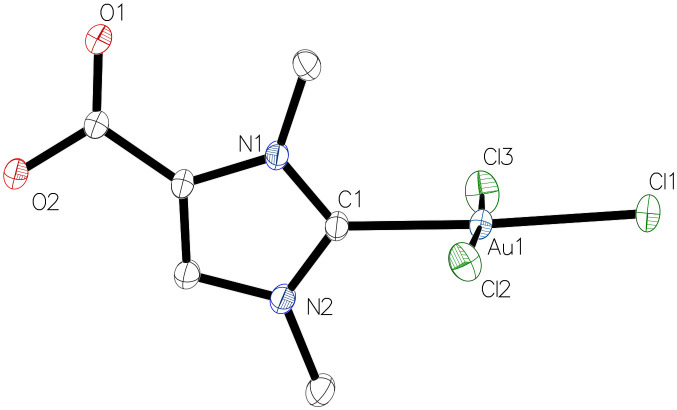
Molecular structure of monocarbene gold(iii) complex 4a with one of the two independent molecules.

The molecular structures of the monocarbene gold(iii) complexes 4a–c were confirmed by X-ray diffraction analysis (Fig. 3, Tables S7–S9†). Complex 4a crystallised in the triclinic space group *P*1̄ with two independent but metrically equal molecules, while 4b and 4c were obtained in the space group *P*2_1_/*c*. The complexes 4a–c were found to have a square planar geometries, as indicated by the linearity of the mutually aligned ligands at the gold centre. Thus, the respective angles Cl1–Au–C1 and Cl2–Au–Cl3 in this order are 176.95(6)° and 174.44(2)° for 4a, 178.89(4)° and 177.234(14) for 4b, 176.53(4)° and 177.747(15) for 4c. The bond lengths Au1−C1 in the gold(i) complexes 2a–c are slightly shorter than in the analogous gold(iii) complexes 4a–c, *i.e.* 1.9849 (12) Å (2a) *vs.* 1.997(2) Å (4a), 1.987(2) Å (2b) *vs.* 2.0022(14) Å (4b), 1.984(2) Å (2c) *vs.* 1.9997(12) (4c).^15^

**Scheme 5:**
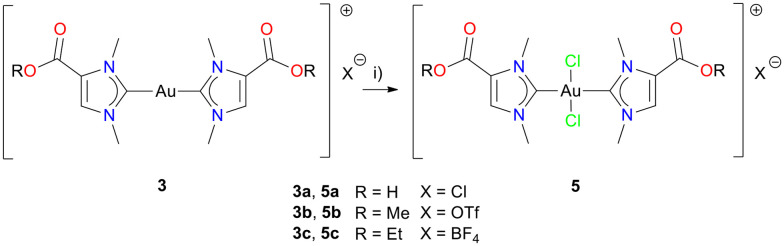
Preparation of dicarbene gold(iii) complexes 5a–c from dicarbene gold(i) complexes 3a–c. Reagents and reaction conditions: i) 1 eq. PhICl_2_, DCM or DCM/MeOH, 24 h, rt.

In analogy to monocarbene gold(iii) complexes 4a–c, the cationic dicarbene gold(iii) complexes 5a–c were obtained with iodobenzene dichloride (PhICl_2_). Complexes 5a–c were purified by column chromatography and obtained as white powders in yields of 70%, 71% and 72%, respectively (Scheme 5). Again, the ^1^H NMR spectra of dicarbene gold(iii) complexes 5a–c display a slight shift of the proton resonances towards lower field (*ca.* 0.05 ppm) compared to the signals of dicarbene gold(i) complexes 3a–c, which can in part be due to the higher oxidation state of the gold centre. In contrast, in the ^13^C{^1^H} NMR spectra the corresponding signals of the distinct C-2 carbene atoms appear at higher field for the gold(iii) complexes 5a–c, *i.e.* 189.6 ppm (3a) *vs.* 157.9 ppm (5a), 188.5 ppm (3b) *vs.* 158.3 ppm (5b), 188.1 ppm (3c) *vs.* 157.9 ppm (5c). This observation is in line with the documented trend for gold(i/iii) NHC complexes.^18–21^

**Fig. 4:**
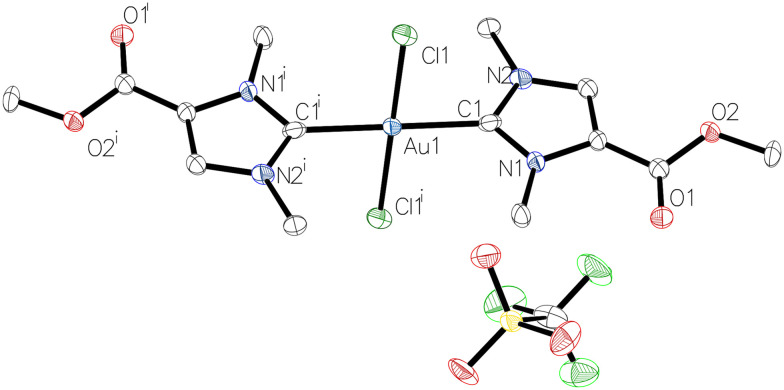
Molecular structure of dicarbene gold(iii) complex 5b. The structure crystallized with two independent half molecules with the gold atom located on an inversion center. The second half of the molecule (C1^i^,…) was generated by the following symmetry operator: 2 − *x*, 2 − *y*, 1 − *z*.

The molecular structures of the dicarbene gold(iii) complexes 5b and 5c were confirmed by X-ray diffraction analysis (Fig. 4, Tables S10 and S11†). The compounds crystallized in the triclinic space group *P*1̄ with two independent units of the complex. The gold atoms in these structures exhibit square planar coordination. The angles C1–Au1–C1′ and Cl1–Au1–Cl1′ display linear alignment resulting from the fact that the gold atoms in these structures are located on inversion centres. The bond lengths Au1–C1/C1′ amount to 2.036(9) Å (5b) and 2.033(3) Å (5c), and are within the range observed for related dicarbene gold(iii) complexes.

As a result of our previous and the current synthetic work, we have established a systematic series of gold complexes 2a–c to 5a–c, in which the carboxylate group of the parent norzooanemonin scaffold 1a is functionalised to a carboxyl (derivatives **a**), methyl ester (**b**) or ethyl ester (**c**) moiety. The series includes monocarbene gold(i/iii) complexes (2a–c, 4a–c) as well as the analogous cationic dicarbene gold(i/iii) complexes (3a–c, 5a–c), giving a total of 12 gold complexes. All complexes have been well characterized including elemental analysis to prove the purity of the materials prior to submission for pharmacological studies. In addition, stability tests were performed on all gold complexes 2a–c to 5a–c (see section S1.4†). Their robustness to possible Au–C bond cleavage and sulphur-nucleophilic attack was demonstrated by ^1^H NMR monitoring. All complexes were found to be stable for at least 24 h at ambient temperature in mixtures of dimethyl sulfoxide and water, which are similar conditions as applied in the pharmacological assays.

### Antiproliferative effects

The antiproliferative potential of the gold–NHC complexes was determined in A549 lung carcinoma cells, MDA-MB-231 breast cancer cells, MCF-7 breast adenocarcinoma cells, and HT-29 colon carcinoma cells (Table 1). Vero E6 African green monkey kidney cells served as a reference cell line to check for possible tumour selectivity. Auranofin (AF) was used as an established reference drug.^15,17^ The antiproliferative efficacy of gold complexes 2a–c to 5a–c showed considerable variation across different cell lines. In A549 cells, the dicarbene complexes 3b–c and 5b–c showed enhanced activity with IC_50_ values in the range of 0.9–5 μM compared to the monocarbene complexes 2b–c and 4b–c in the range of 10–36 μM. Similarly, for MDA-MB-231 cells, the dicarbene complexes exhibited IC_50_ values ranging from 2–19 μM for 3b–c and 5b–c, whereas the monocarbene complexes 2b–c and 4b–c had IC_50_ values in the range of 8–32 μM.

**Table 1:** Antiproliferative activities of gold complexes expressed as IC_50_ values in μM with standard deviations in parentheses (*n* = 3)^a^

Entry	A549	HT-29	MCF-7	MDA-MB-231	Vero E6
2a	60.0^b^	63.9^b^	53.4^b^	76.1^b^	n.d.
2b	22.67 (1.91)	19.83 (2.02)	23.81 (2.44)	12.82 (3.87)	n.d.
2c	10.38 (2.87)	22.05 (5.95)	9.93 (2.08)	8.90 (2.60)	13.11 (0.82)
3a	n.d.	n.d.	n.d.	n.d.	n.d.
3b	5.21 (1.30)	27.74 (2.32)	34.35 (5.99)	19.20(0.49)	>100
3c	1.07 (0.16)	5.78 (2.00)	4.64 (0.88)	2.51 (0.57)	31.54 (2.58)
4a	36.12 (5.52)	>90	47.84 (2.33)	31.78 (2.40)	42.41 (2.88)
4b	17.74 (2.05)	26.16 (3.38)	15.18 (0.95)	15.18 (2.08)	22.23 (1.04)
4c	17.93 (1.59)	28.38 (0.71)	14.63 (0.98)	11.05 (1.42)	20.66 (0.70)
5a	>100	>100	>100	>100	>100
5b	3.35 (0.89)	18.59 (3.14)	20.00 (6.58)	14.83 (1.18)	>100
5c	0.89 (0.10)	4.29 (0.62)	3.91 (0.83)	2.33 (0.15)	30.79 (0.49)
AF	3.53 (0.52)	4.32 (0.79)	1.89 (0.26)	0.80 (0.17)	2.40 (0.14)

a n.d.: not determined, AF: auranofin.

b Ref. 17.

In contrast, HT-29 and MCF-7 cells showed weaker responses and less pronounced differences between mono- and dicarbene complexes, with the dicarbene gold complexes 3b–c and 5b–c achieving effects within the range of 4–34 μM and with the monocarbene gold complexes 2b–c and 4b–c showing IC_50_ values of 10–50 μM. Interestingly, complexes 3c and 5c displayed greater cytotoxicity in A549 cells compared to the gold reference compound auranofin (AF). In line with previous observations (*vide supra*), the dicarbene gold complexes 3b–c and 5b–c demonstrated remarkable efficacy in cytotoxicity.^12,14,18,22^ A clear trend for gold(i) *vs.* gold(iii) complexes, *i.e.*2a–c and 3a–c compared to 4a–c and 5a–c is not observed. Overall, we found the respective esters (2–5)**b** and (2–5)**c** to be more active compared to the non-alkylated derivatives (2–5)**a**. In particular, for the dicarbene complex series 3a–c and 5a–c, the activity increases systematically in the order of increased lipophilicity. Thus, in the series R = H → Me → Et as carboxylate substituents the activity follows the order (3 or 5)**a** < (3 or 5)**b** < (3 or 5)**c** throughout all cell lines tested. In addition, there was a clear preference for the tumour cell lines in comparison to the non-tumour Vero E6 cell line for the dicarbene complexes 3b, 3c, 5b, and 5c, which was not evident for the monocarbene complexes.

### Antibacterial studies

The antibacterial activity of all gold complexes was assessed against six pathogenic bacterial strains, including two Gram-positive species, *i.e. Enterococcus faecium* and methicillin-resistant *Staphylococcus aureus* (MRSA), and four Gram-negative species, *i.e. Acinetobacter baumannii*, *Escherichia coli*, *Klebsiella pneumoniae*, and *Pseudomonas aeruginosa*. Minimum inhibitory concentrations (MICs) were determined using a curve-fitting technique. In general, monocarbene gold(i/iii) complexes demonstrated moderate to high activity, while dicarbene gold(i/iii) complexes were inactive (Tables 2 and S12†).

**Table 2:** Antibacterial activities of monocarbene gold(i/iii) complexes 2a–c and 4a–c. Minimal inhibitory concentrations (MIC) are given in μM^a^

Entry	E.f.	MRSA	A.b.	E.c.	K.p.	P.a.
2a	>100	>100	43 ± 0	86 ± 0	43 ± 7	>100
2b	10 ± 0	10 ± 0	10 ± 0	41 ± 0	41 ± 0	>100
2c	10 ± 0	10 ± 0	10 ± 2	40 ± 0	40 ± 0	>100
4a	>100	>100	36 ± 3	72 ± 0	36 ± 6	>100
4b	17 ± 0	17 ± 0	9 ± 2	35 ± 0	35 ± 0	>100
4c	17 ± 0	17 ± 2	8 ± 1	34 ± 0	34 ± 5	>100
AF	0.3	0.4	55	46	81	>100
ABa	9.5	4.7	1	0.1	0.2	7

a E.f. = *Enterococcus faecium* DSM20477, MRSA = methicillin-resistant *Staphylococcus aureus* DSM 11822, A.b. = *Acinetobacter baumannii* DSM30007, E.c. = *Escherichia coli* DSM 1116, K.p. = *Klebsiella pneumoniae* DSM111678, P.a. = *Pseudomonas aeruginosa* DSM 24068. As positive control antibiotics, amikacin (for P.a.), linezolid (for MRSA) and ciprofloxacin (for all other strains) have been used.

The carboxyl monocarbene gold(i/iii) complexes 2a and 4a did not exhibit any activity against Gram-positive bacteria (*E. faecium*, MRSA) and *P. aeruginosa* (MIC > 100 μM), and only moderate activity against other Gram-negative bacteria with MIC values ranging from 36 μM to 86 μM. However, compared to 2a and 4a, the alkylated complexes 2b,c and 4b,c show elevated activity. The monocarbene gold(i) complexes 2b and 2c exhibited the highest potency, both having MIC values of 10 μM against *E. faecium*, MRSA, and *A. baumannii*. They also showed moderate activity against *E. coli* and *K. pneumoniae* with MIC values of 41 μM for 2b and MIC values of 40 μM for 2c. However, both complexes were inactive against *P. aeruginosa* (MIC > 100 μM). The monocarbene NHC–Au(iii) complexes 4b and 4c also showed notable activity against Gram-positive bacteria (*E. faecium*, MRSA) with MIC values of 17 μM for 4b and 17 μM for 4c, while their activity against *A. baumannii* was even higher (MIC = 9 μM for 4b, and MIC = 8 μM for 4c). However, their activity against other Gram-negative bacteria was moderate, with MIC values of 35 μM for *E. coli* and *K. pneumoniae* in the case of 4b, and 34 μM for *E. coli* and *K. pneumoniae* in the case of 4c, respectively, while they also remained inactive against *P. aeruginosa* (MIC > 100 μM). Overall, gold(i) complexes demonstrated slightly better activity against both Gram-positive and Gram-negative bacteria compared to gold(iii) complexes.

### Inhibition of bacterial TrxR

The inhibition of isolated TrxR from *E. coli* was determined for the selected monocarbene gold(i/iii) complexes 2a–c and 4b–c using a DTNB-based assay (Table 3). As previously reported,^15,17^ NHC–Au complexes are capable of inhibiting the isolated enzyme at low micromolar concentrations. The inhibition of bacterial TrxR for compound 2a was investigated in our previous report (Table 3). Complexes 2b–c and 4b–c showed stronger inhibition of TrxR than the parent compound 2a and comparable to auranofin used as a standard control, again confirming the relationship between lipophilicity and activity according to our initial predictions. Compound 2c shows the strongest inhibition at half of the IC_50_ level compared to auranofin. Gold(iii) compounds 4b and 4c showed slightly weaker inhibition of the enzyme. For all other compounds tested, no activity against TrxR was detected at a concentration of 0.5 μM (see ESI†). Interestingly, the results of the enzyme inhibition studies are in excellent agreement with the antibacterial activity of complexes against Gram-positive and Gram-negative pathogens, with the strongest TrxR inhibitors 2b and 2c showing the lowest MIC values. Such findings support the previous suggestions of TrxR inhibition as a relevant mechanism of bactericidal action of gold complexes.

**Table 3:** Inhibition of bacterial TrxR by monocarbene gold(i/iii) complexes expressed as IC_50_ values (μM) with standard deviation (*n* = 3)

Entry	IC_50_ [μM]
Auranofin	0.210 ± 0.030
2a	0.786 ± 0.118
2b	0.293 ± 0.163
2c	0.133 ± 0.044
4b	0.362 ± 0.033
4c	0.381 ± 0.053

### Gold cellular uptake

The amount of potential drug accumulated in the cell is often described as one of the critical factors for further biological effect. The significant difference in antiproliferative effect observed for dicarbene complexes 5a–c may be directly related to the amount of gold ions taken up by the treated cells. The cellular accumulation of these complexes was evaluated in the most sensitive cancer cell line A549. Considering the resulting IC_50_ values, the confluent cell layer was treated with 2 μM of each selected compound for 6 and 24 h at 37 °C. The gold content was further quantified using high-resolution continuum source atomic absorption spectroscopy (HRCS-AAS).

**Fig. 5:**
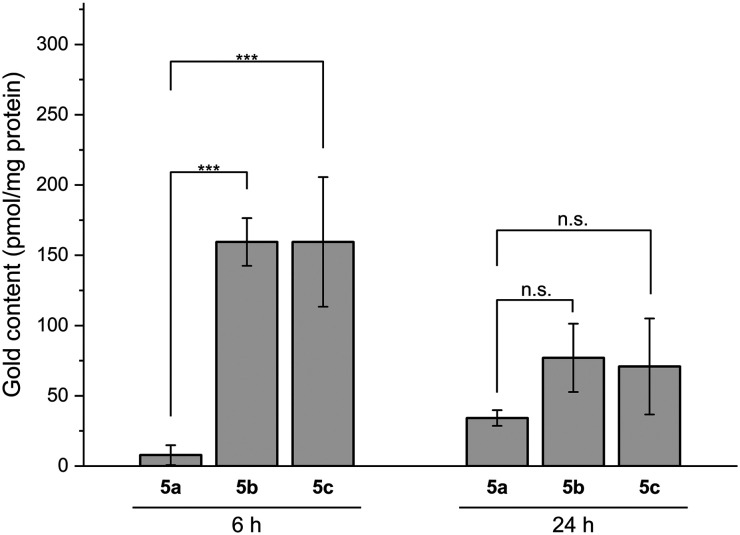
Uptake studies of complexes 5a–c at 2 μM in A549 cells after treatment for 6 and 24 h. The data represent a mean of three independent experiments. The symbol *** indicates a significant difference between the gold concentration in cells treated with selected complexes under the same conditions at the same time point (*p* < 0.001, based on paired Student's *t*-test). For the other conditions, no significant difference was observed (n.s. = not significant, *p* > 0.05).

The resulting uptake study (Fig. 5) revealed highly significant (*p* < 0.001) difference in gold content after cell treatment with 5a and 5b,c for 6 h. The low gold uptake in the case of the former complex may explain the significantly lower toxicity in comparison to the latter compounds. The results are in line with our working hypothesis that more lipophilic complexes 5b,c display a higher degree of gold uptake. Interestingly, however, after 24 h the amount of the metal taken up decreased significantly for 5b,c but not for 5a, so that almost no difference (*p* > 0.05) was observed between all three samples. Such an effect of significant gold excretion has been previously reported for some gold dicarbene complexes.^14^ The metabolic pathways of gold elimination in cells may include degradation of the complex or protein–metal conjugate followed by transfer *via* efflux transporters.^23^

## Conclusions

Norzooanemonin (1a, 1a′) is a naturally occurring marine betaine and has previously been employed in the synthesis of mono- and dicarbene gold(i) complexes 2a and 3a.^17^ The poor antimicrobial and TrxR inhibitory activity of 2a and 3a has been attributed to their high polarity and poor cell membrane penetration. In an attempt to increase lipophilicity, the carboxylate group in the parent norzooanemonin 1a was alkylated to produce methyl ester 1b and ethyl ester 1c, respectively. From the precursors 1a–c, a systematic series of 12 gold carbene complexes 2a–c to 5a–c with an inherent norzooanemonin scaffold were obtained in analytically pure form. The series includes the monocarbene complexes 2a–c and 4a–c as well as the cationic dicarbene complexes 3a–c and 5a–c with variable gold(i) or gold(iii) oxidation states.

In antiproliferative, antimicrobial and TrxR inhibition assays, the esters, *i.e.* compounds (2–5)**b**,**c**, show a much better overall activity compared to the parent compounds (2–5)**a**, confirming the established working hypothesis that elevated lipophilicity leads to increased bioactivity. Examples include the dicarbene ethyl esters 3c and 5c, which exhibit much higher antiproliferative activities against A549 lung cancer cells than the reference compound auranofin. Furthermore, the monocarbene esters 2b–c and 4b–c display high antimicrobial activities against *A. baumannii*, again outperforming the standard auranofin. Our findings are consistent with the general observations that i) monocarbene gold complexes are stronger inhibitors of bacterial TrxR and microbial activity compared to cationic dicarbene gold complexes, (ii) the dicarbene gold complexes are superior in antiproliferative activity throughout various cancer cell lines, and (iii) NHC–gold(i) complexes have similar activity compared to NHC–gold(iii) complexes. A key finding of our study is the superior performance of the ethyl esters in dicarbene gold (i/iii) complexes against various cancer cell lines studied, as found in the stated order of (3 or 5)**a** < (3 or 5)**b** < (3 or 5)**c**. Moreover, the cancer cell selectivity noted for the dicarbene complexes 3b, 3c, 5b and 5c is of particular interest regarding the development of this type of organometallics to tumour-selective anticancer drug candidates. Future investigations will focus on the preparation of higher esters, *i.e.* alkyl ester moieties with carbon atom numbers ≥3 (linear or branched) with increased lipophilicity.

## Data availability

The data supporting this article have been included as part of the ESI.† Crystallographic data have been deposited at the CCDC data base under the entry codes 2355435 (1b), 2355436 (1c), 2355437 (2b), 2355438 (2c), 2355439 (3b), 2355440 (3c), 2355441 (4a), 2355442 (4b), 2355443 (4c), 2355444 (5b) and 2355445 (5c) and can be obtained from the web page https://www.ccdc.cam.ac.uk/ free of charge.

## Conflicts of interest

There is no conflict of interest to declare.

## Supplementary Material

MD-OLF-D4MD00358F-s001

MD-OLF-D4MD00358F-s002
