# Supplementary material for: Gold(i) and gold(iii) carbene complexes from the marine betaine norzooanemonin: inhibition of thioredoxin reductase, antiproliferative and antimicrobial activity
Source: RSC Med Chem. 2024 Jul 31;15(9):3248–55. doi: 10.1039/d4md00358f (PMC11342128; doi:10.1039/d4md00358f)
Supplement: MD-015-D4MD00358F-s001 [file MD-015-D4MD00358F-s001.pdf]

Supplementary Information (SI) for RSC Medicinal Chemistry.
This journal is © The Royal Society of Chemistry 2024

Supporting Information

Gold(I) and Gold(III) Carbene Complexes from the Marine Betaine Norzooanemonin: Inhibition of Thioredoxin Reductase, Antiproliferative and Antimicrobial Activity

CONTENTS

S1.1. General 1

S1.2. Experimental Procedures 2

S1.2.1 Synthesis of Compound 1b 2

S1.2.2. Synthesis of compound 1c 4

S1.2.3. Synthesis of compound 2b 6

S1.2.4. Synthesis of compound 2c 8

S1.2.5. Synthesis of compound 3b 10

S1.2.6. Synthesis of compound 3c 13

S1.2.7. Synthesis of compound 4a 15

S1.2.8. Synthesis of compound 4b 17

S1.2.9. Synthesis of compound 4c 19

S1.2.10. Synthesis of compound 5a 21

S1.2.11. Synthesis of compound 5b 23

S1.2.12. Synthesis of compound 5c 26

S1.3. Single Crystal X-Ray Diffraction 29

S1.4. Stability Tests of Gold Complexes 41

S2.1. Antibacterial Studies 44

S2.2. Cytotoxicity and Anticancer Studies 45

S2.3. Inhibition of bacterial TrxR from *E. coli* 46

S2.4. Cellular uptake and metal quantification studies in A549 cells 47

S3.1. References 48

S1.1. General

Materials and Analytical Methods

All materials were purchased from Aldrich, Wako or TGI. $(Me_2S)AuCl$ was prepared according to literature procedure.[1] Norzooanemonin, gold complexes (**3c**), and (**5c**) have been prepared according to our previous report.[2] All reactions were carried under air and technical grade solvent were used unless otherwise stated. K_2CO_3 and NaOH were used as received without further purification. ^{1}H and ^{13}C NMR spectra were measured on the spectrometers Bruker AV 300 (300 MHz) spectrometers. The chemical shifts are given in parts per million (δ; ppm) relative to residual solvent peaks (δ; 3.31 (CD_3OD), 4.79 (D_2O), 7.26 ($CDCl_3$), 2.50 (DMSO-D_6). Coupling constants (*J*) are reported in Hertz (Hz), and splitting patterns are indicated as s (*singlet*), d (*doublet*), t (*triplet*), m (*multiplet*), sept (*septet*) and br (*broad*). All the spectra were measured at room temperature unless otherwise stated. Elemental analyses of all the complexes were carried out with a Vario Micro Cube System (Elementar Analysensysteme GmbH). High- and Low-resolution electrospray ionization (ESI) measurements were made with a Bruker MicroTOF II mass spectrometer.

S1.2. Experimental Procedures

S1.2.1 Synthesis of Compound 1b

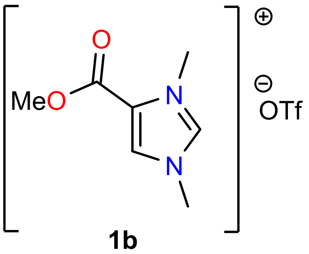

1b

Methyl trifluoromethanesulfonate (0.11 mL, 1 mmol, 1 eq.) was added to norzooanemonin **1a** (0.140 g, 1 mmol, 1 eq.) in dichloromethane and stirred for one hour at room temperature. The solvent was removed, and compound **1b** was washed with cold ethanol and hexane to be obtained as a white solid (0.120 g, 86%). ^{1}H NMR ($CDCl_3$, 300.1 MHz, 293 K): *δ.* 9.00 (1H, s, CH_{im}), 7.98 (1H, s, CH_{im}), 4.02 (3H, s, CH_3), 3.90 (3H, s, CH_3), 3.84 (3H, s, CH_3). ^{13}C{^{1}H} NMR ($CDCl_3$, 75.5 MHz, 293 K): *δ.* 157.6 (*C*OO), 140.5 (N*C*HN), 129.0 (N*C*HC), 124.3 (N*C*COO), 120.3 (q, $^{1}J_{C,F}$= 320 MHz, *C*F_3), 52.7 (O*C*H_3), 36.5 (N*C*H_3), 36.3 (N*C*H_3). Elemental analysis calculated (%) for $C_8H_{11}F_3N_2O_5S$: C, 31.58, H, 3.64, N, 9.21; found: C, 31.02, H, 3.61, N, 9.10. ESI-MS *m/z* anion: calculated for $[M]^+$ 155.08; Found $[M]^+$ 155.14 m/z.

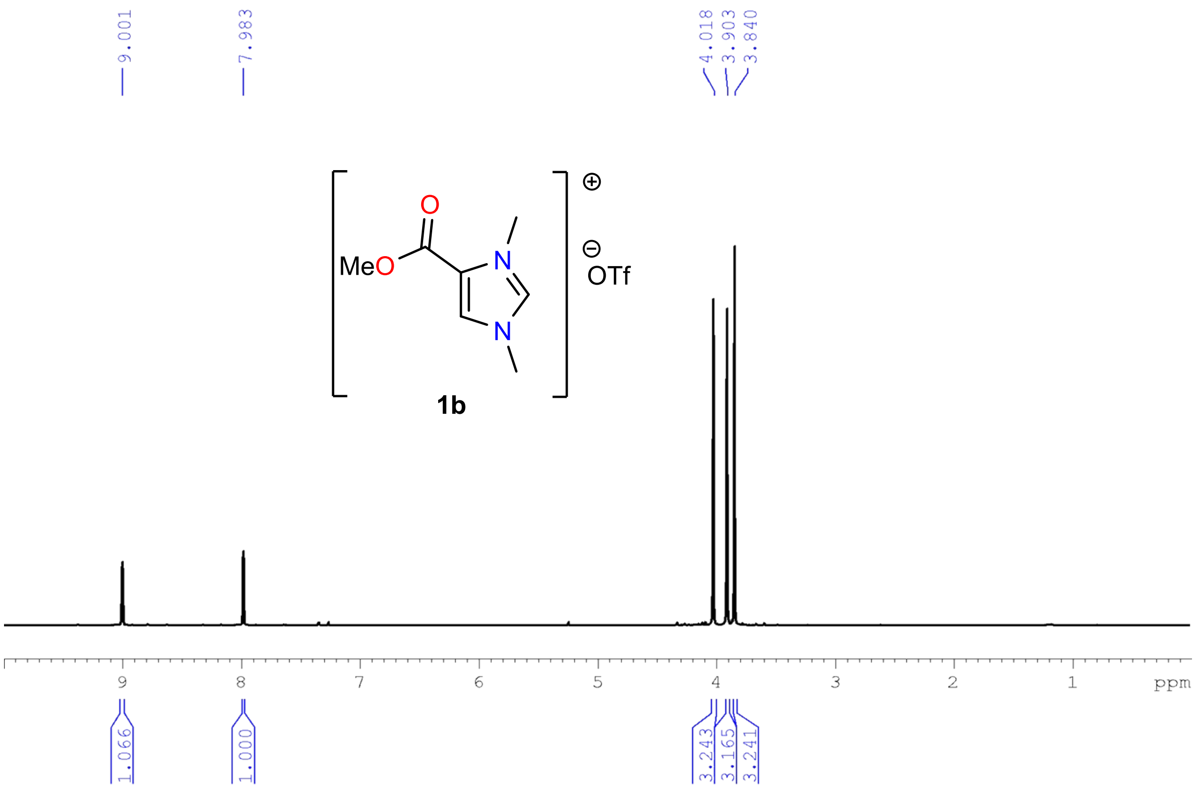

Figure S1. ^{1}H NMR spectrum of **1b** in $CDCl_3$ at room temperature.

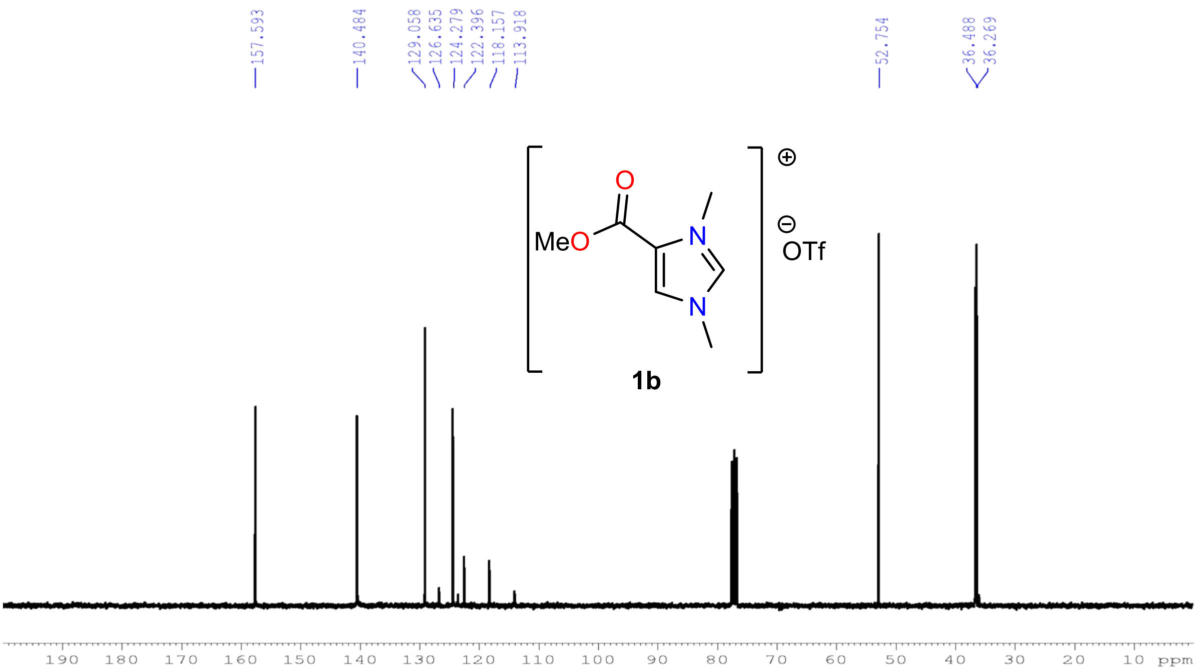

Figure S2. $^{13}C\{^1H\}$ NMR spectrum of **1b** in $CDCl_3$ at room temperature.

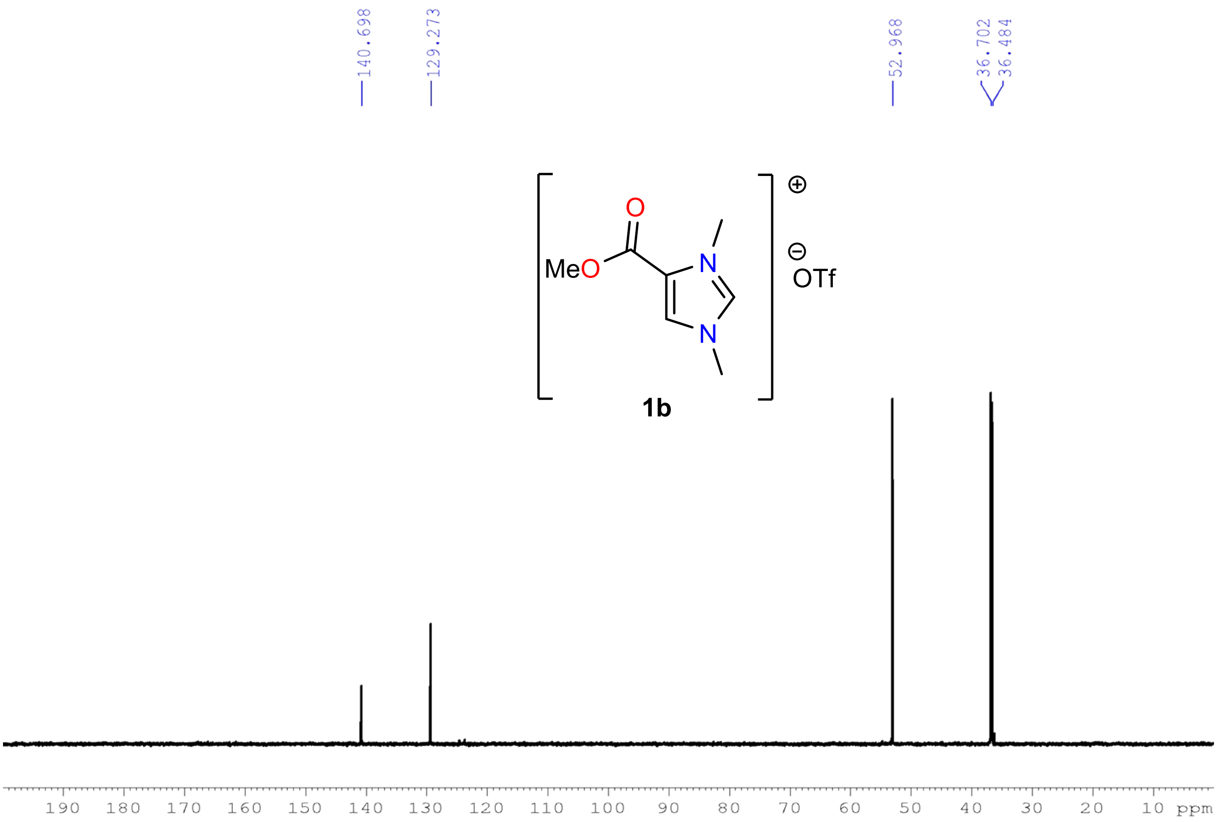

Figure S3. ^{13}C dept NMR spectrum of **1b** in $CDCl_3$ at room temperature.

S1.2.2. Synthesis of compound 1c

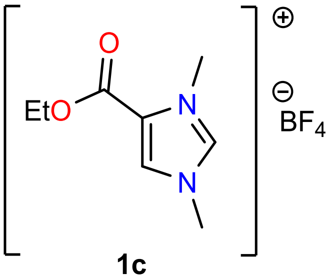

1c

Triethyloxonium tetrafluoroborate (0.190 g, 1 mmol, 1. eq.) was added to norzooanemonin **1a** (0.140 g, 1 mmol, 1. eq.) in dichloromethane and stirred for four hours. Upon removing the solvent and washing with cold ethanol and diethyl ether, compound **1c** was obtained a white solid (0.130 g, 51%). ^{1}H NMR ($CDCl_3$, 300.1 MHz, 293 K): *δ.* 8.84 (1H, s, CH_{im}), 7.89 (1H, s, CH_{im}), 4.37 (2H, q, $^3J_{HH}$= 7.2 Hz, CH_2CH$_3$), 4.09 (3H, s, CH_3), 3.96 (3H, s, CH_3), 1.36 (3H, t, $^3J_{HH}$= 7.1 Hz, CH$_2$$CH_3$). ^{13}C{^{1}H} NMR ($CDCl_3$, 75.5 MHz, 293 K): *δ.* 157.3 (*C*OO), 140.7 (N*C*HN), 128.8 (N*C*HC), 124.7 (N*C*COO), 62.5 (CH_2CH$_3$), 36.7 (NCH_3), 36.5 (NCH_3), 13.9 (CH_3CH$_2$). Elemental analysis calculated (%) for $C_8H_{13}F_4N_2O_2$: C, 37.53, H, 5.12, N, 10.94; found: C, 37.21, H, 5.07, N, 10.89. ESI-MS *m/z* anion: calculated for $[M]^+$ 169.10; Found $[M]^+$ 169.12 m/z.

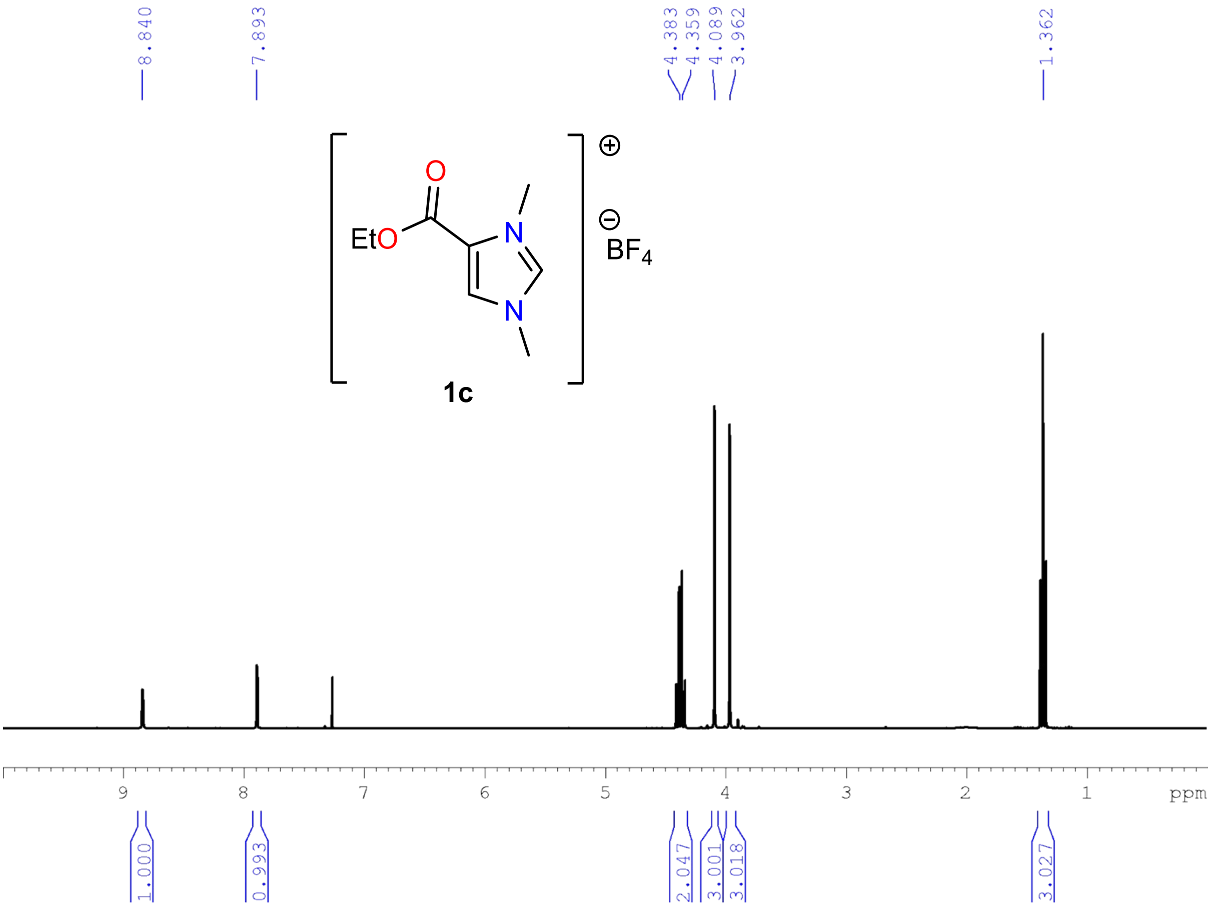

Figure S4. ^{1}H NMR spectrum of **1c** in $CDCl_3$ at room temperature.

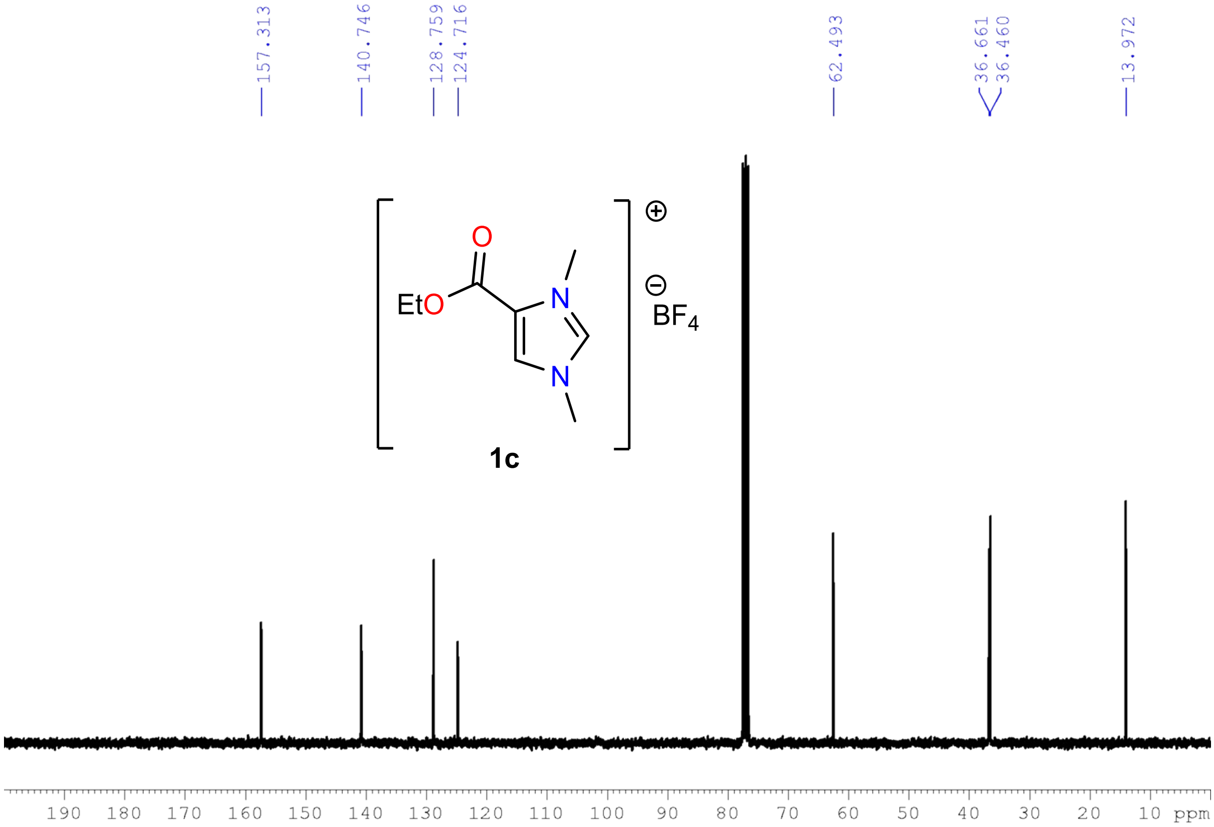

Figure S5. $^{13}C\{^{1}H\}$ NMR spectrum of **1c** in $CDCl_3$ at room temperature.

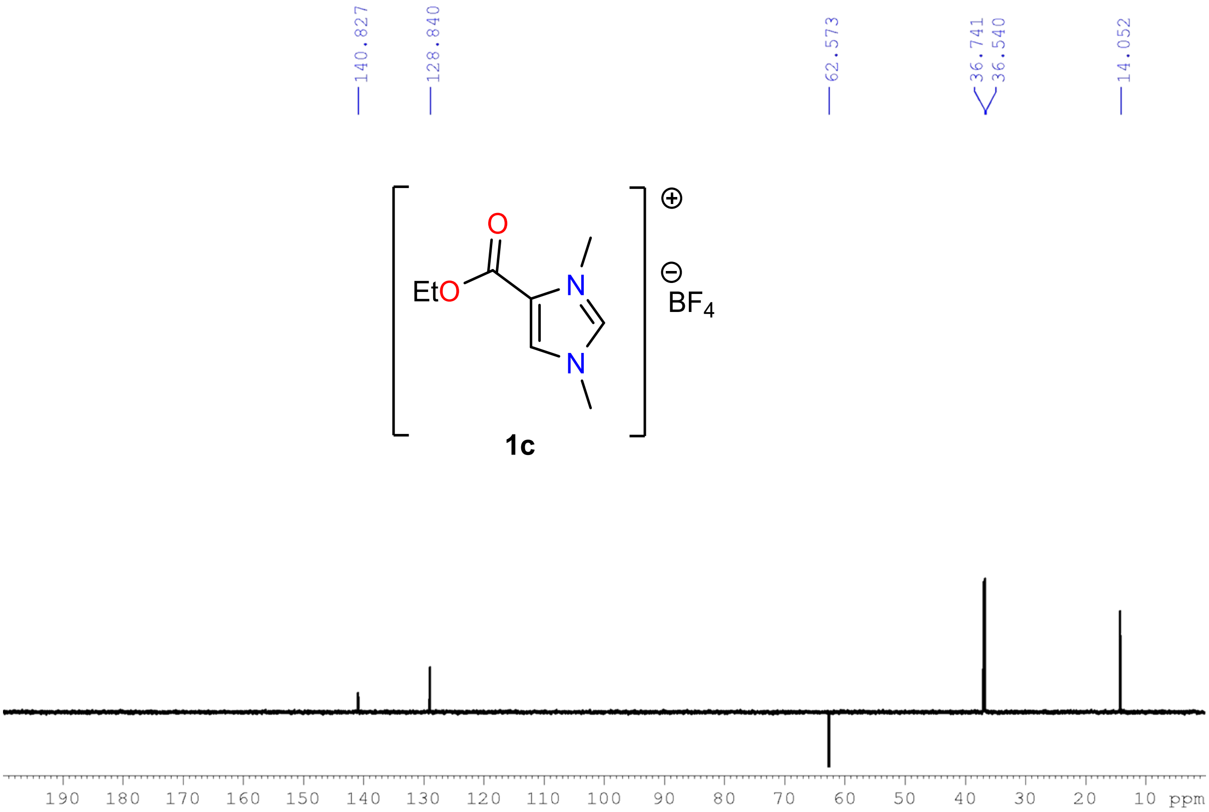

Figure S6. ^{13}C dept NMR spectrum of **1c** in $CDCl_3$ at room temperature.

S1.2.3. Synthesis of compound 2b

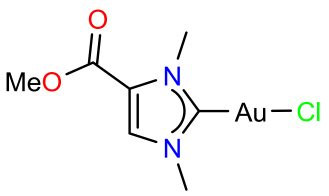

2b

A mixture of compound **1b** (0.076 g, 0.25 mmol, 1 eq.) and K_2CO_3 (0.034 g, 0.25 mmol, 1 eq.) was stirred in methanol for three hours at room temperature. (Me_2S)AuCl (0.073 g, 0.25 mmol, 1 eq) was added to the mixture and stirred for 12 hours. The brownish mixture was filtered using a Whatman filter, and the solvent was removed under vacuum. Complex **2b** was obtained as a white powder after chloroform extraction and solvent removal under vacuum (0.048 g, 50%). ^{1}H NMR ($CDCl_3$, 300.1 MHz, 293 K): *δ.* 7.59 (1H, s, CH_{im}), 4.09 (3H, s, CH_3), 3.88 (3H, s, CH_3), 3.87 (3H, s, CH_3). $^{13}C\{^1H\}$ NMR ($CDCl_3$, 75.5 MHz, 293 K): *δ.* 176.7 (*C*Au), 158.7 (*C*OO), 128.3 (N*C*HC), 123.9 (N*C*COO), 52.4 (O*C*H_3), 38.8 (N*C*H_3), 38.2 (N*C*H_3). Elemental analysis calculated (%) for $C_7H_{10}AuClN_2O_2$: C, 21.75, H, 2.61, N, 7.25; found: C, 21.88, H, 2.89, N, 7.18. ESI-MS *m/z* anion: calculated for $[M + Na]^+$ 409.01; Found $[M + Na]^+$ 409.08 m/z and calculated for $[M – CH_3]^-$ 370.99; Found $[M – CH_3]^-$ 370.68 m/z.

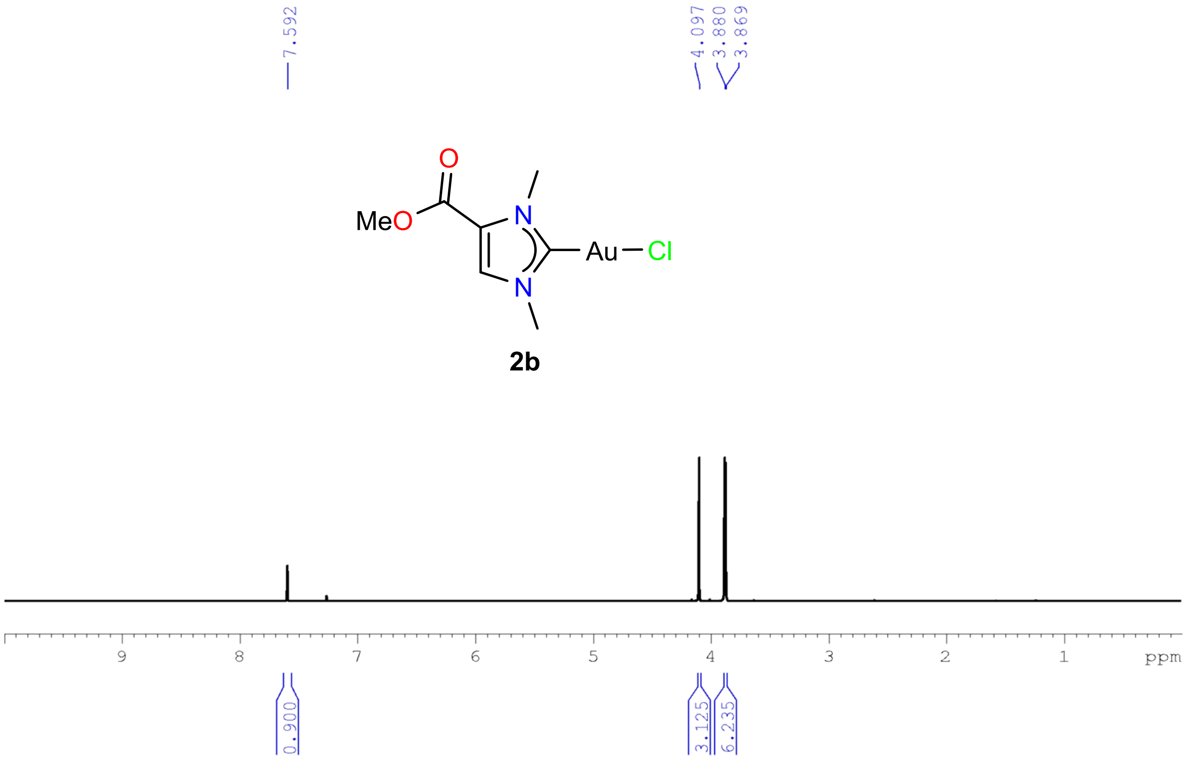

Figure S7. ^{1}H NMR spectrum of **2b** in $CDCl_3$ at room temperature.

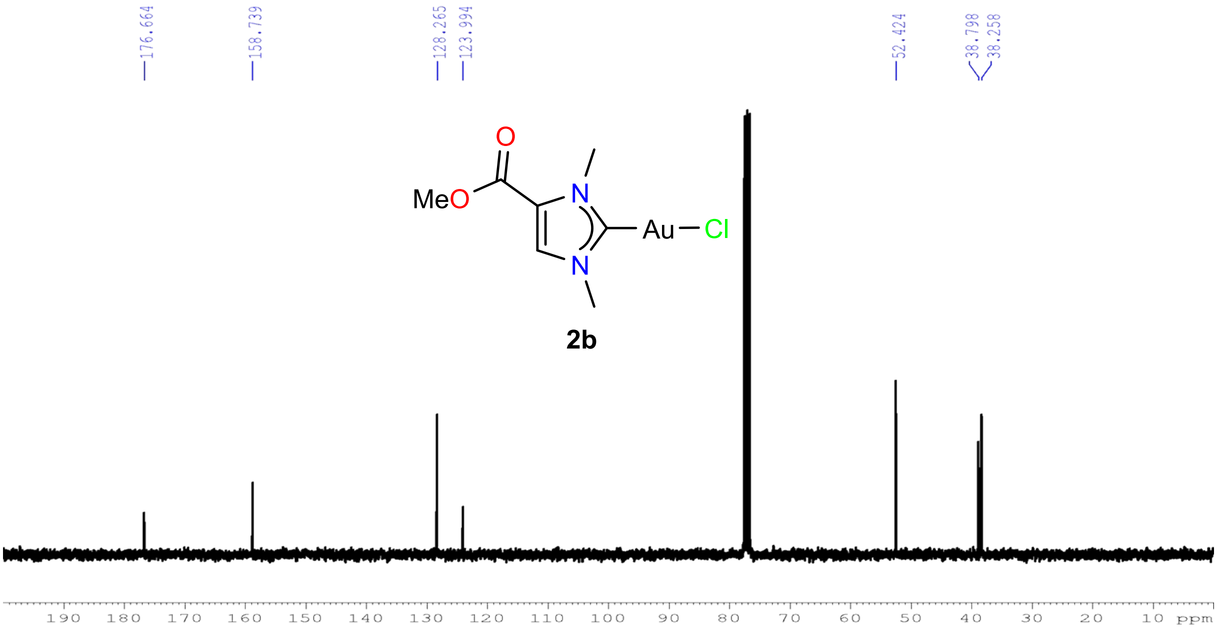

Figure S8. $^{13}C\{^{1}H\}$ NMR spectrum of **2b** in $CDCl_3$ at room temperature.

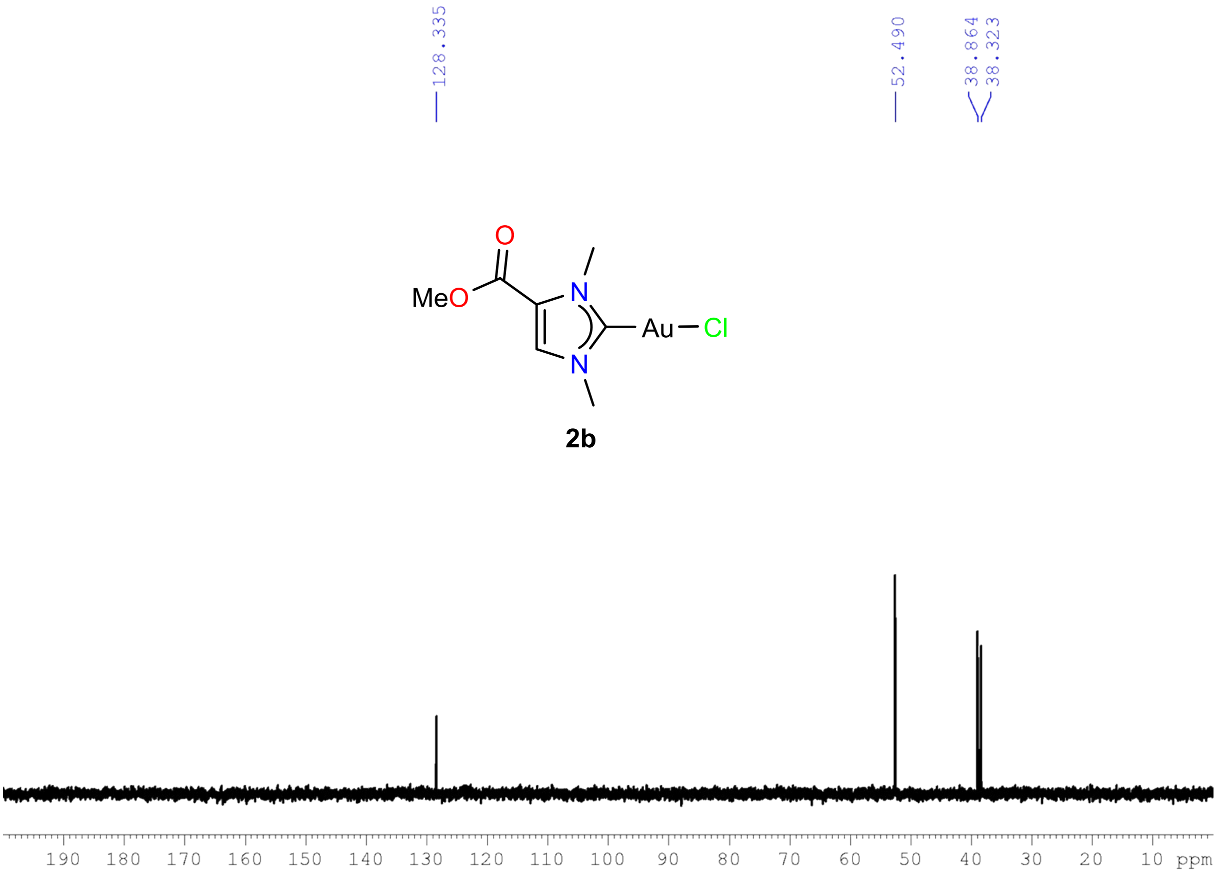

Figure S9. ^{13}C dept NMR spectrum of **2b** in $CDCl_3$ at room temperature.

S1.2.4. Synthesis of compound 2c

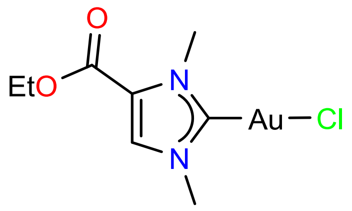

2c

At 50 °C overnight, K_2CO_3 (0.034 g, 0.25 mmol, 1 eq.) was dissolved in ethanol. Compound **1c** (0.064 g, 0.25 mmol, 1 eq.) and $(Me_2S)AuCl$ (0.074 g, 0.25 mmol, 1 eq.) were added at room temperature and stirred overnight. The brownish mixture was filtered and the ethanol was removed under vacuum to yield a white powder. Extraction with chloroform and removal of the solvent yielded complex **2c** as a white powder (0.046 g, 46%).^{1}H NMR ($CDCl_3$, 300.1 MHz, 293 K): *δ.* 7.59 (1H, s, CH_{im}), 4.35 (2H, q, $^3J_{HH}$= 7.2 Hz, CH_2CH$_3$), 4.10 (3H, s, CH_3), 3.87 (3H, s, CH_3), 1.36 (3H, t, $^3J_{HH}$= 7.1 Hz, CH$_2$$CH_3$). ^{13}C{^{1}H} NMR ($CDCl_3$, 75.5 MHz, 293 K): *δ.* 176,6 (*C*Au), 158.3 (*C*OO), 128.1 (N*C*HC), 124.3 (N*C*COO), 61.7 (*C*H$_2$CH$_3$), 38.7 (N*C*H$_3$), 38.3 (N*C*H$_3$), 14.2 (*C*H$_3$CH$_2$). Elemental analysis calculated (%) for $C_8H_{12}AuClN_2O_2$: C, 23.79, H, 3.02, N, 6.99; found: C, 23.89, H, 2.99, N, 6.89. ESI-MS *m/z* anion: calculated for $[M + Na]^+$ 423.02; Found $[M + Na]^+$423.00 m/z and calculated for $[M – H]^-$ 399.02; Found $[M – H]^-$ 398.92 m/z.

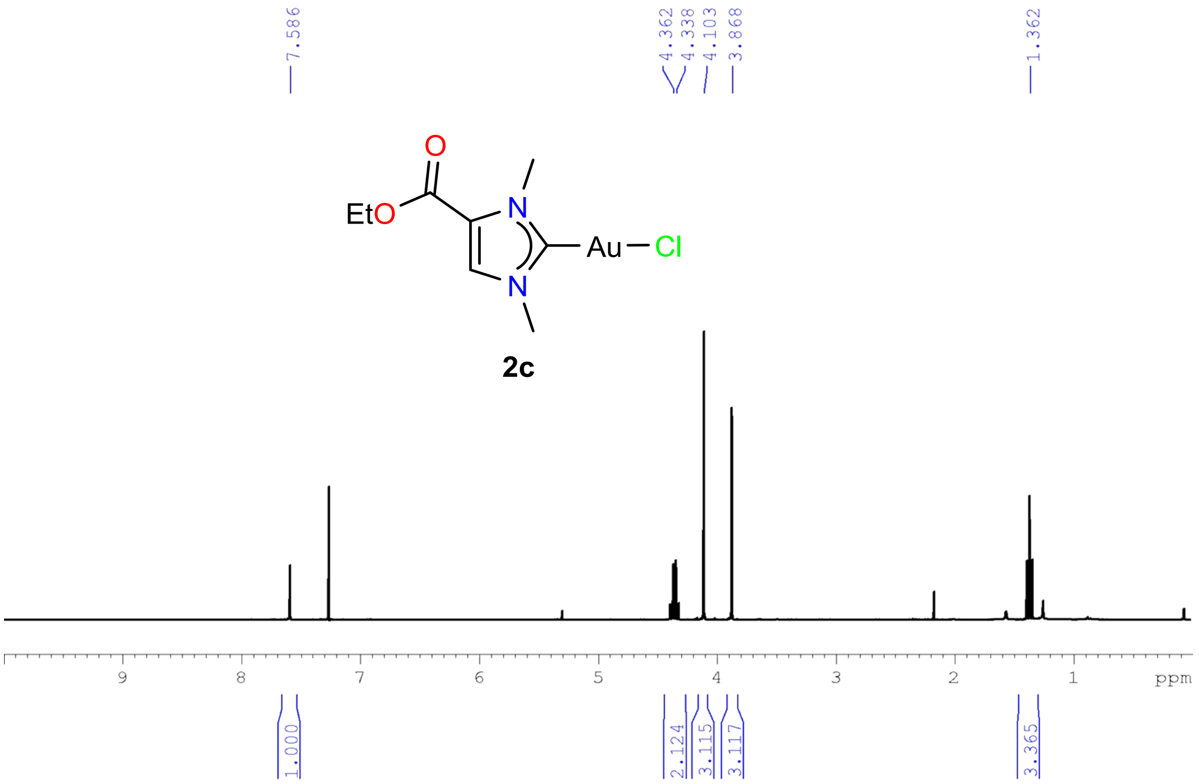

Figure S10. ^{1}H NMR spectrum of **2c** in $CDCl_3$ at room temperature.

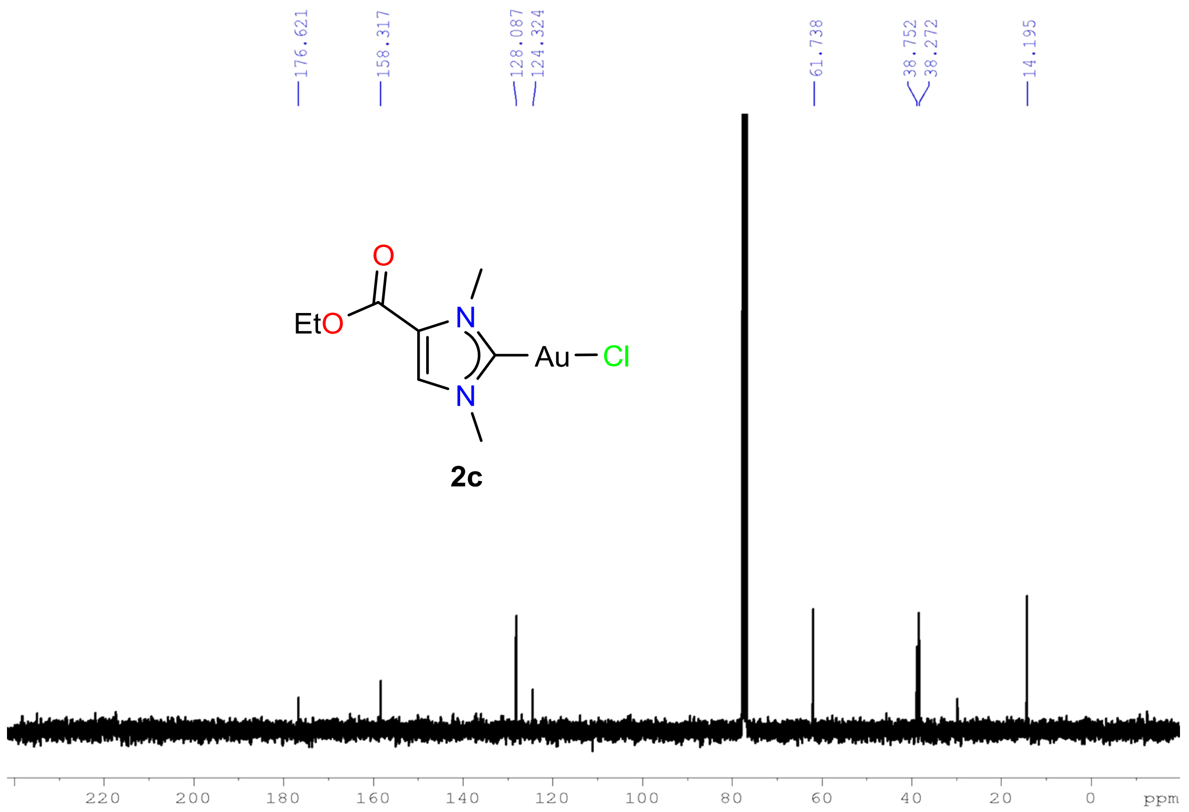

Figure S11. $^{13}C\{^{1}H\}$ NMR spectrum of **2c** in $CDCl_3$ at room temperature.

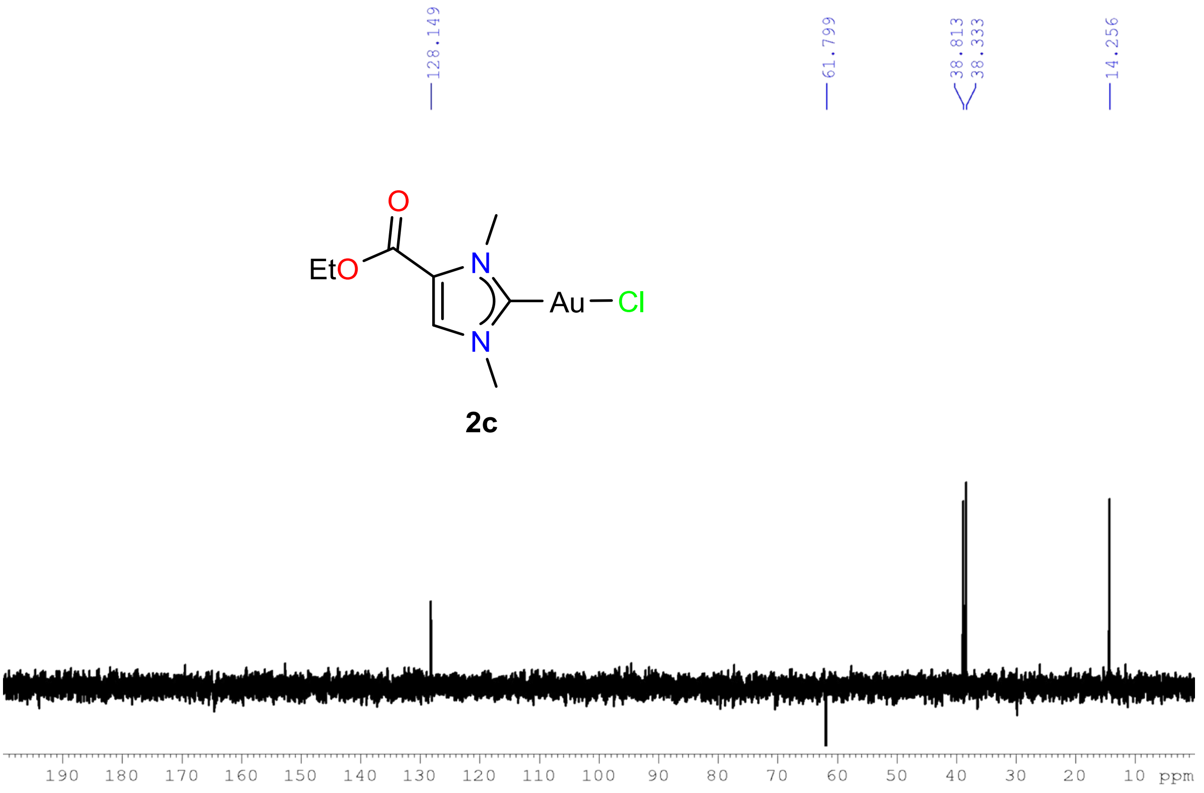

Figure S12. ^{13}C dept NMR spectrum of **2c** in $CDCl_3$ at room temperature.

S1.2.5. Synthesis of compound 3b

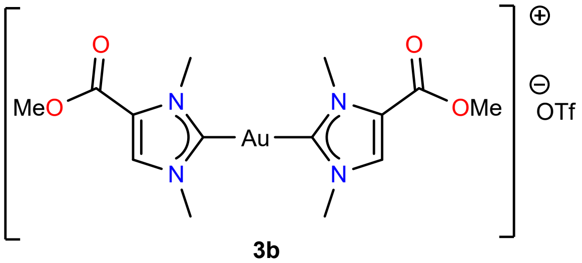

3b

Compounds **1b** (0.228 g, 0.70 mmol, 2 eq.), K_2CO_3 (0.104 g, 0.70 mmol, 2 eq.), and $(Me_2S)AuCl$ (0.103 g, 0.35 mmol, 1 eq.) were stirred in acetone for 7 days, resulting in a brownish mixture which was then filtered using a Whatman filter. The solvent of the filtrate was removed under vacuum, following column chromatography (99% DCM: 1% MeOH), and complex **3b** was obtained as a white powder (0.015 g, 66%). ^{1}H NMR (CD_2Cl_2, 300.1 MHz, 293 K): *δ.* 7.77 (1H, s, CH_{im}), 4.15 (3H, s, CH_3), 3.95 (3H, s, CH_3), 3.89 (3H, s, CH_3). $^{13}C\{^{1}H\}$ NMR (CD_2Cl_2, 75.5 MHz, 293 K): *δ.* 188.5 (*C*Au), 159.2 (*C*OO), 129.6 (N*C*HC), 124.9 (N*C*COO), 52.8 (O*C*H_3), 38.9 (N*C*H_3), 38.4 (N*C*H_3). Elemental analysis calculated (%) for $C_{15}H_{20}AuF_3N_4O_7S$: C, 27.53, H, 3.08, N, 8.56; found: C, 27.72, H, 3.12, N, 8.48. ESI-MS m/*z* anion: calculated for $[M]^+$ 505.11; Found $[M]^+$ 505.20 m/z.

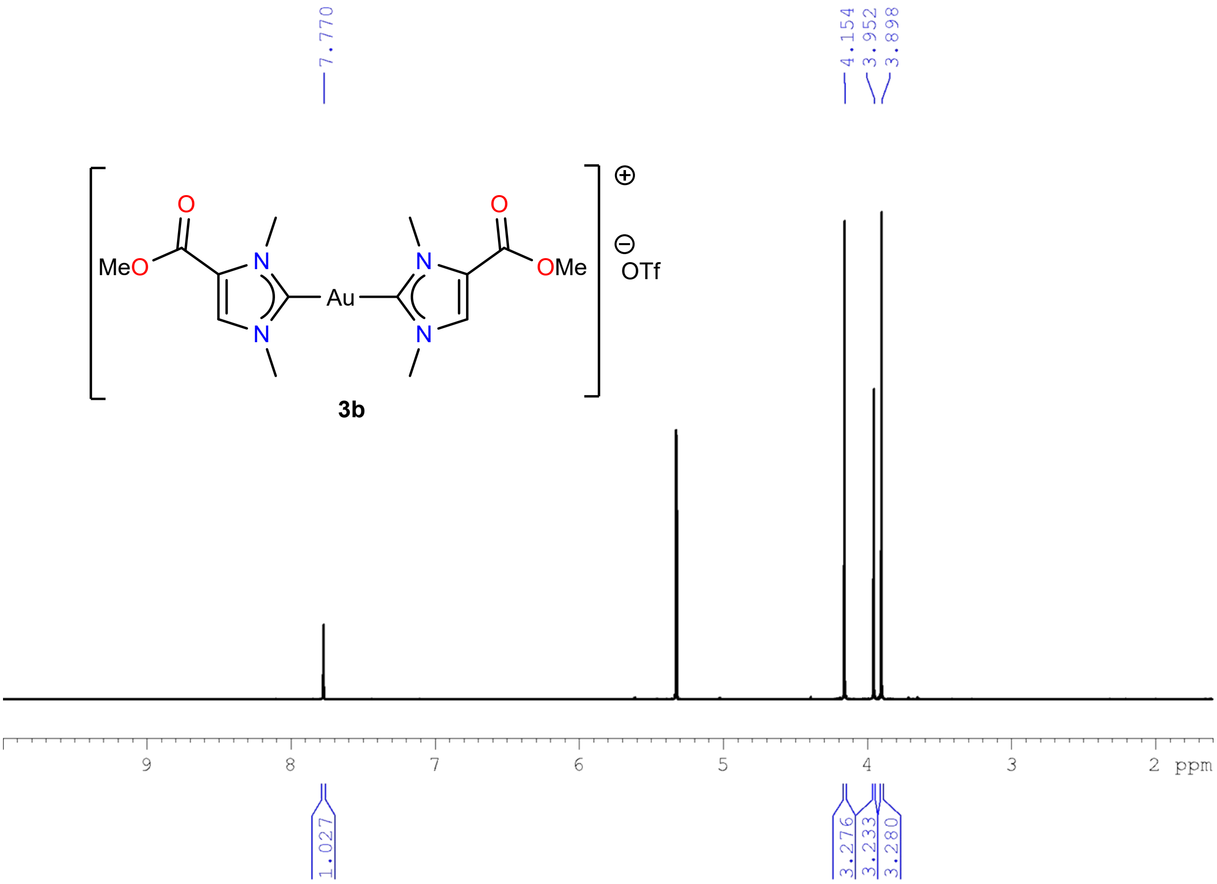

Figure S13. ^{1}H NMR spectrum of **3b** in CD_2Cl_2 at room temperature.

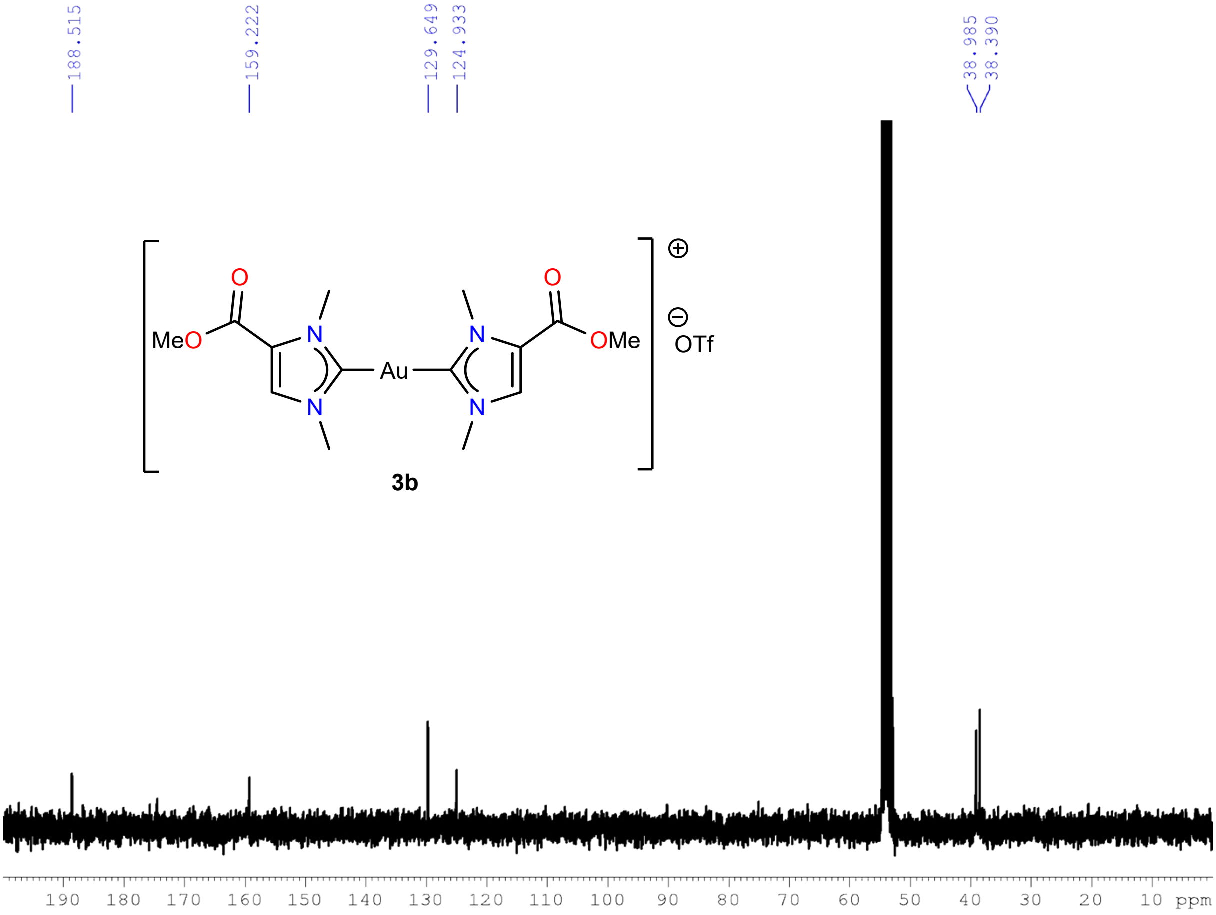

Figure S14. $^{13}C\{^{1}H\}$ NMR spectrum of **3b** in CD_2Cl_2 at room temperature.

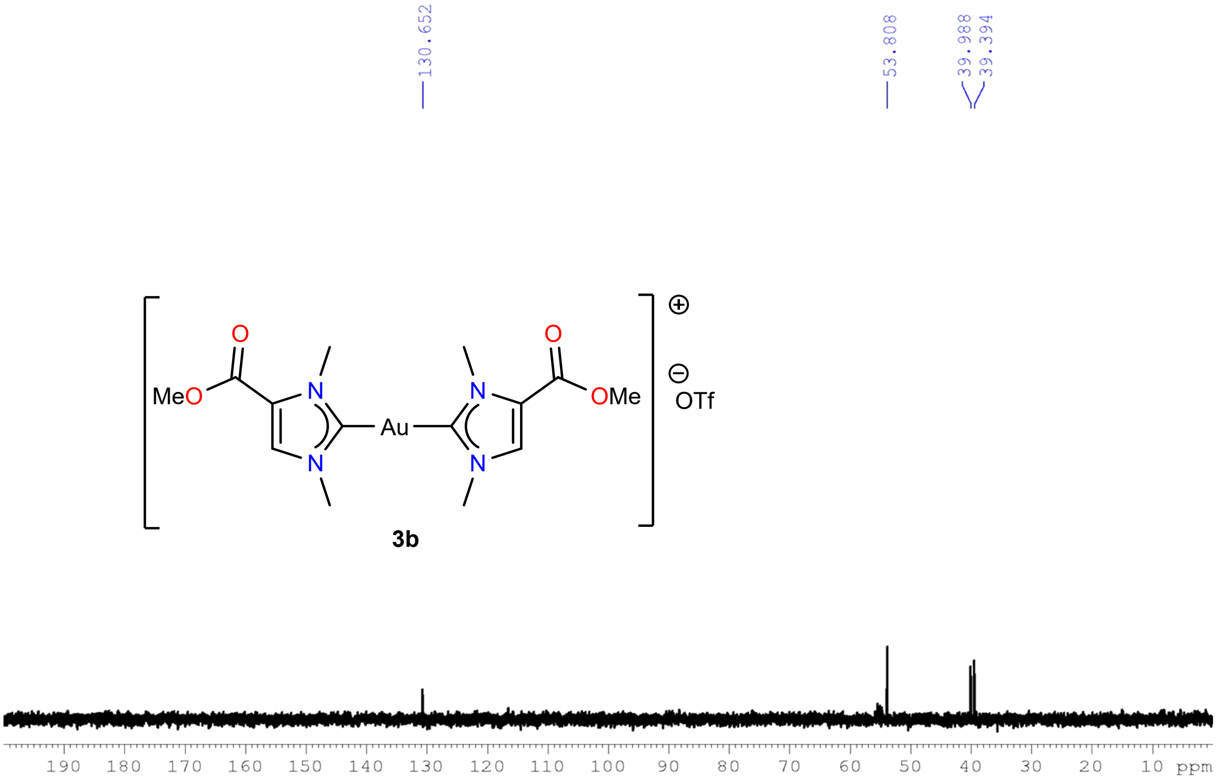

Figure S15. ^{13}C dept NMR spectrum of **3b** in CD_2Cl_2 at room temperature.

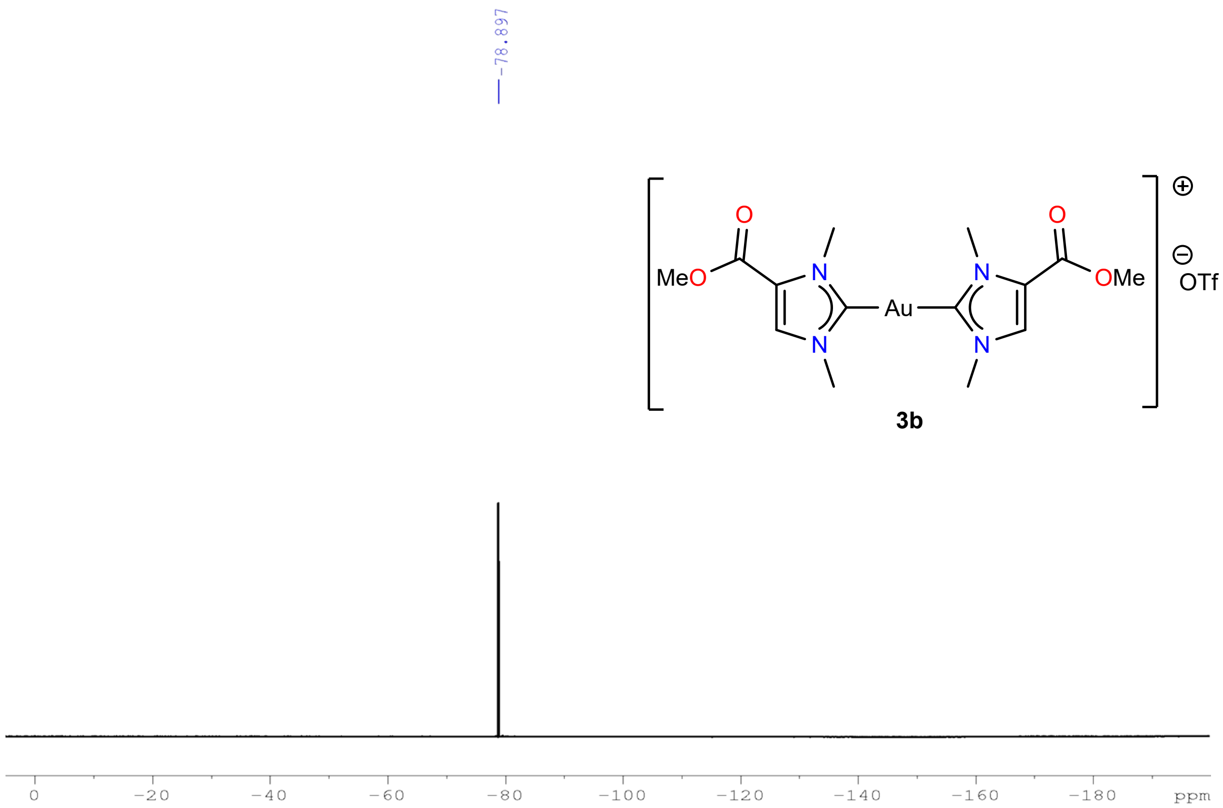

Figure S16. ^{19}F NMR spectrum of **3b** in CD_2Cl_2 at room temperature.

S1.2.6. Synthesis of compound 3c

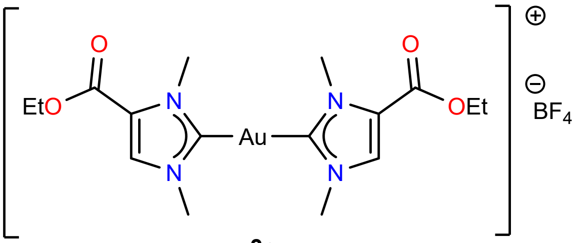

3c

Compound **1c** (1 eq, 0.128 g, 0.5 mmol, 1 eq.), K_2CO_3 (0.069 g, 0.5 mmol, 1 eq.), and $(Me_2S)AuCl$ (0.057 g, 0.25 mmol, 0.5 eq.) were stirred in acetone for four days at room temperature to form a brownish mixture. The solution was filtered using a Whatman filter and the solvent of filtrate was removed under vacuum. After purification by column chromatography (99% DCM: 1% MeOH), complex **3c** was obtained as a white powder (0.084 g, 54%). ^{1}H NMR ($CDCl_3$, 300.1 MHz, 293 K): *δ.* 7.79 (1H, s, CH_{im}), 4.35 (2H, q, $^3J_{HH}$= 7.1 Hz, CH_2CH_3), 4.15 (3H, s, CH_3), 4.00 (3H, s, CH_3), 1.37 (3H, t, $^3J_{HH}$= 7.1 Hz, CH_2CH_3). $^{13}C\{^1H\}$ NMR ($CDCl_3$, 75.5 MHz, 293 K): *δ.* 188.1 (*C*Au), 158.5 (*C*OO), 129.5 (N*C*HC), 124.6 (N*C*COO), 61.8 (*C*H_2CH_3), 38.7 (N*C*H_3), 38.0 (N*C*H_3), 14.1 (*C*H_3CH_2). Elemental analysis calculated (%) for $C_{16}H_{24}AuBF_4N_4O_4$: C, 30.99, H, 3.90, N, 9.03; found: C, 30.91, H, 3.89, N, 9.08. ESI-MS m/*z* anion: calculated for $[M]^+$ 533.15; Found $[M]^+$ 533.25 m/z.

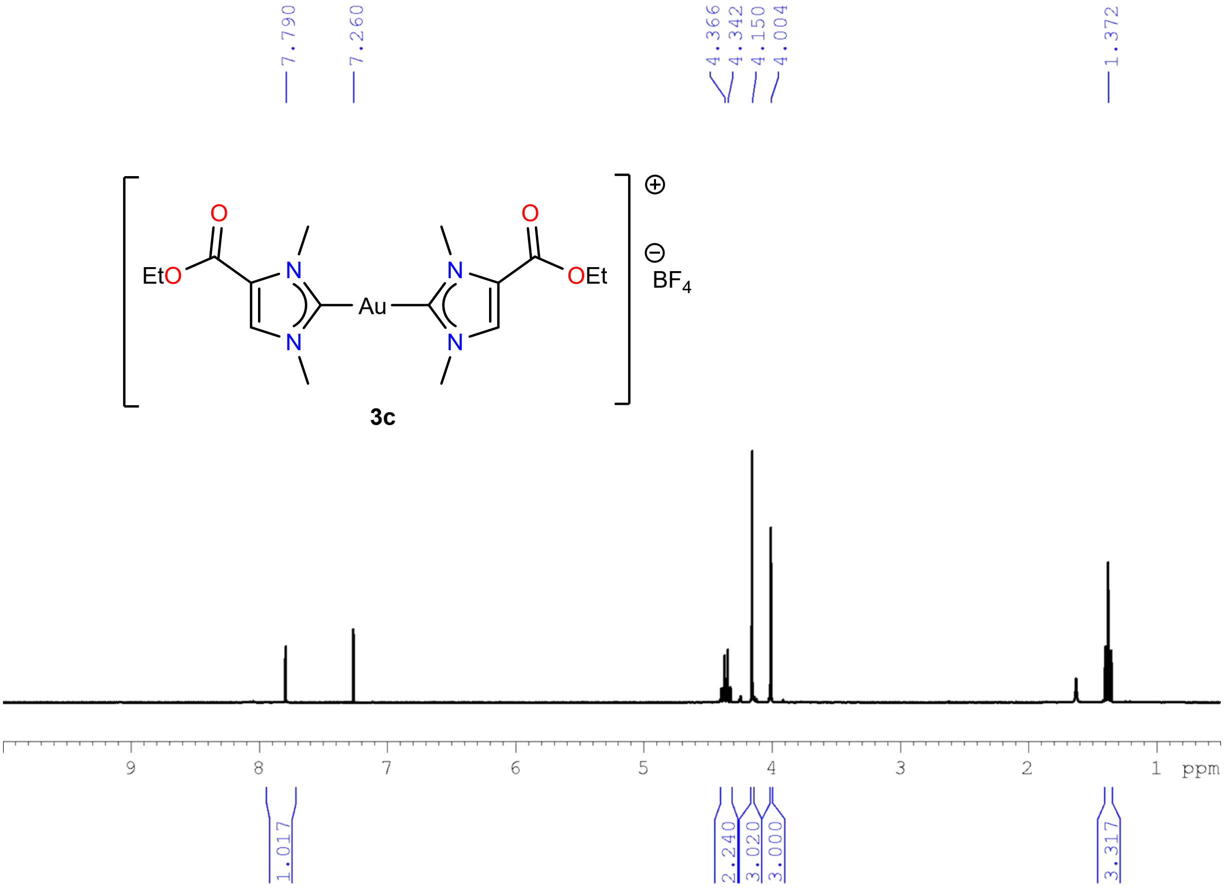

Figure S17. ^{1}H NMR spectrum of **3c** in $CDCl_3$ at room temperature.

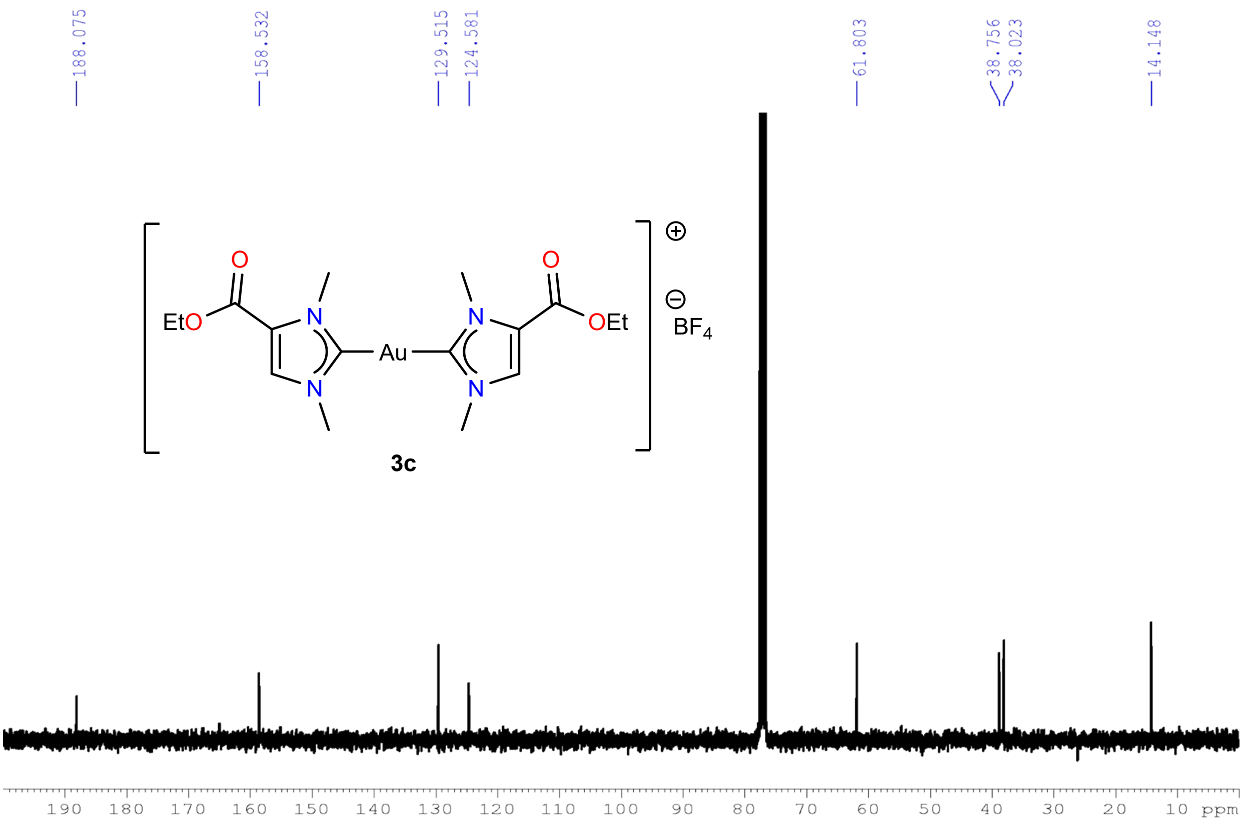

Figure S18. $^{13}C\{^{1}H\}$ NMR spectrum of **3c** in $CDCl_3$ at room temperature.

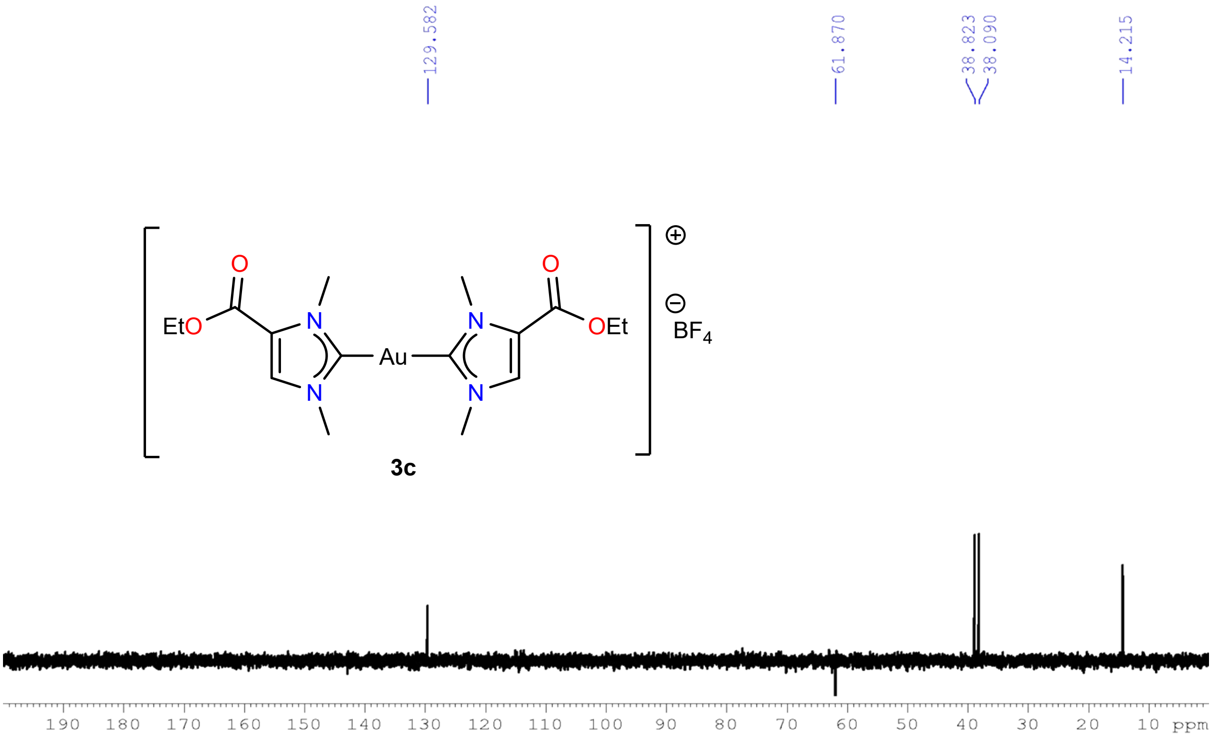

Figure S19. ^{13}C dept NMR spectrum of **3c** in $CDCl_3$ at room temperature.

S1.2.7. Synthesis of compound 4a

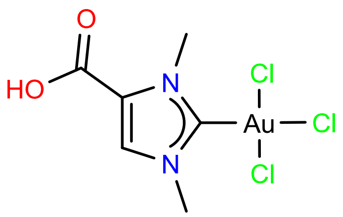

4a

A mixture of MeOH/DCM (1:1) was used to dissolve compound **2a** (0.039, 0.1 mmol, 1 eq.). Iodobenzene dichloride (0.038 g, 0.14 mmol, 1.4 eq.) was added, and the mixture was stirred for 24 hours at room temperature. The resulting light-yellow solution was vacuum evaporated and washed three times with hexane (20 mL) and diethyl ether (20 mL) to obtain complex **4a** as a white powder (0.038 g, 85%). ^{1}H NMR (CD_3OD, 300.1 MHz, 293 K): *δ.* 8.21 (1H, s, CH_{im}), 4.21 (3H, s, CH_3), 3.99 (3H, s, CH_3). $^{13}C\{^{1}H\}$ NMR (CD_3OD, 75.5 MHz, 293 K): *δ.* 160.2 (*C*OO), 146,4 (*C*Au), 132.2 (N*C*HC), 128.5 (N*C*COO), 38.4 (N*C*H_3), 37.7 (N*C*H_3). Elemental analysis calculated (%) for $C_6H_8AuCl_3N_2O_2$: C, 16.25, H, 1.82, N, 6.32; found: C, 16.17, H, 1.717, N, 5.78. ESI-MS *m/z* anion: calculated for $[M + Na]^+$ 464.92; Found $[M + Na]^+$ 464.86 m/z.

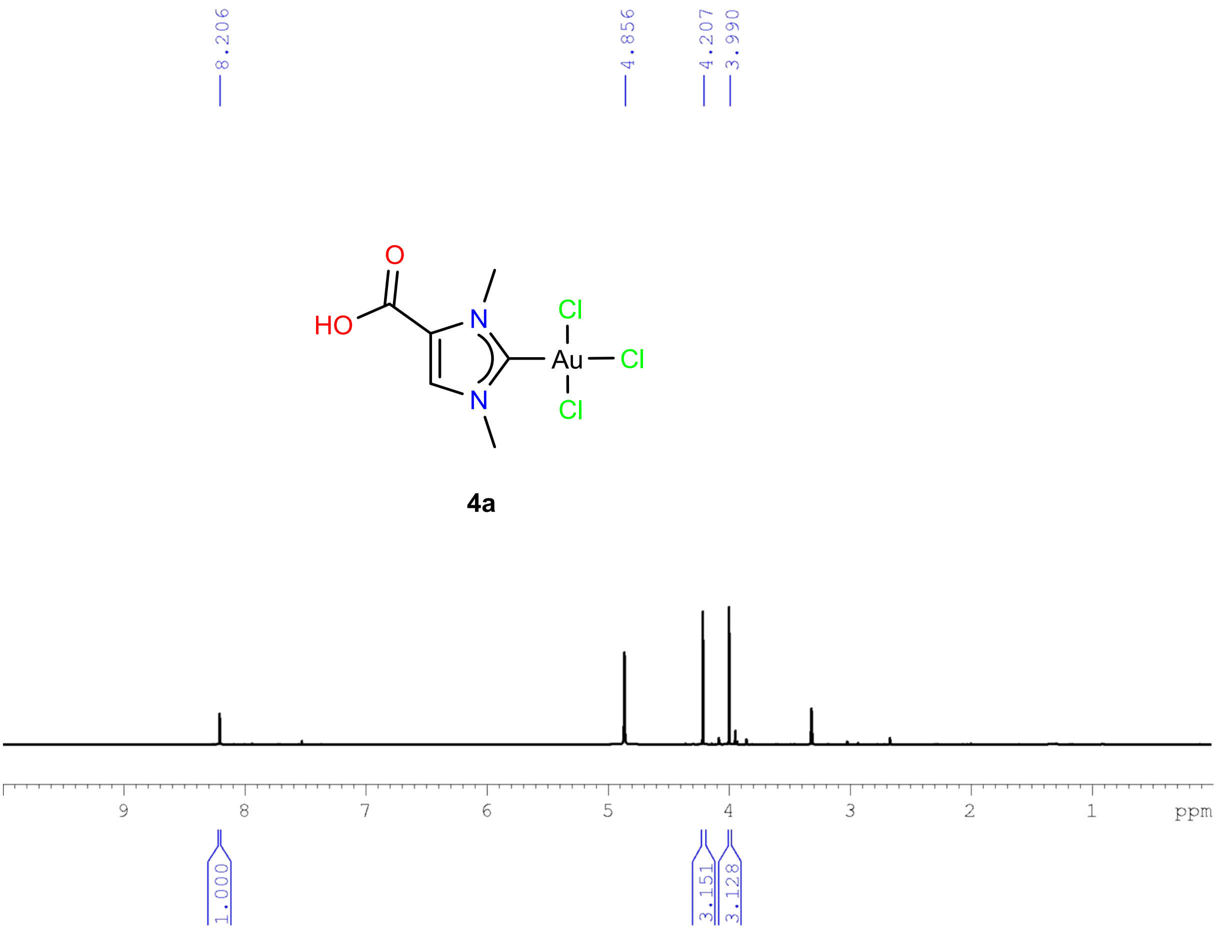

Figure S20. ^{1}H NMR spectrum of **4a** in CD_3OD at room temperature.

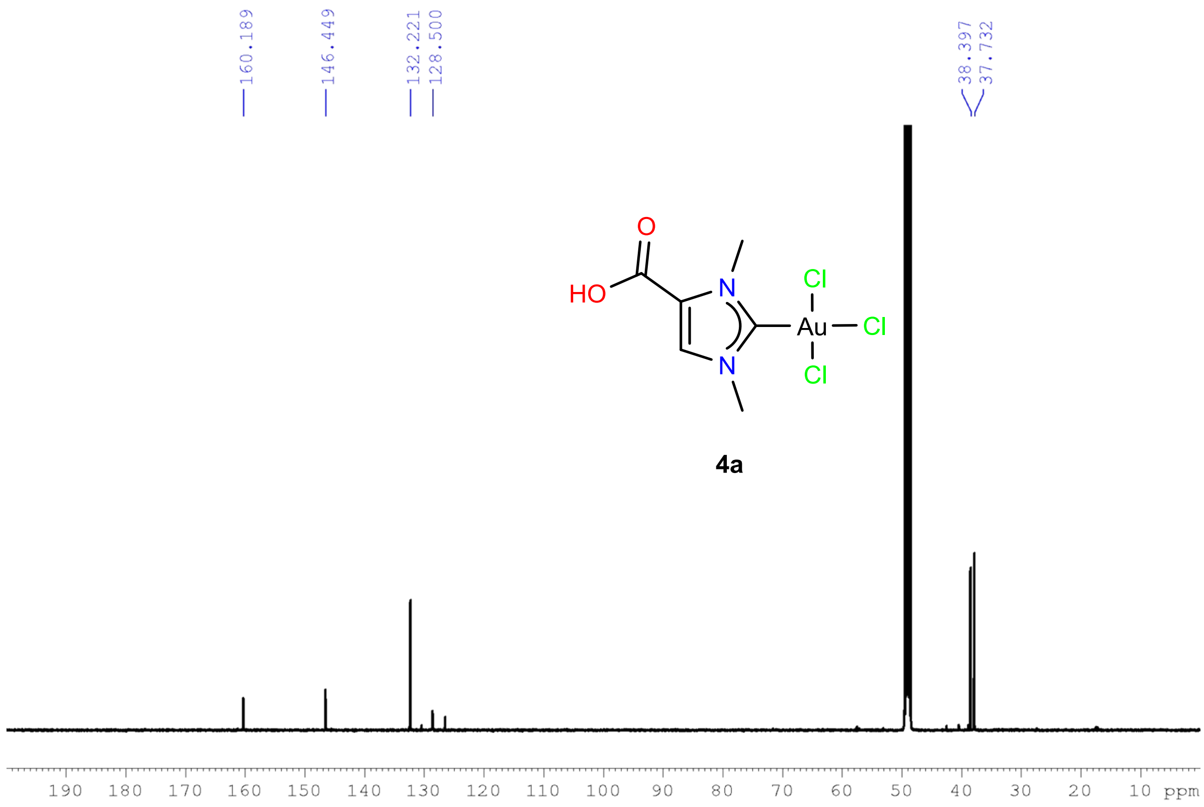

Figure S21. $^{13}C\{^{1}H\}$ NMR spectrum of **4a** in MeOD at room temperature.

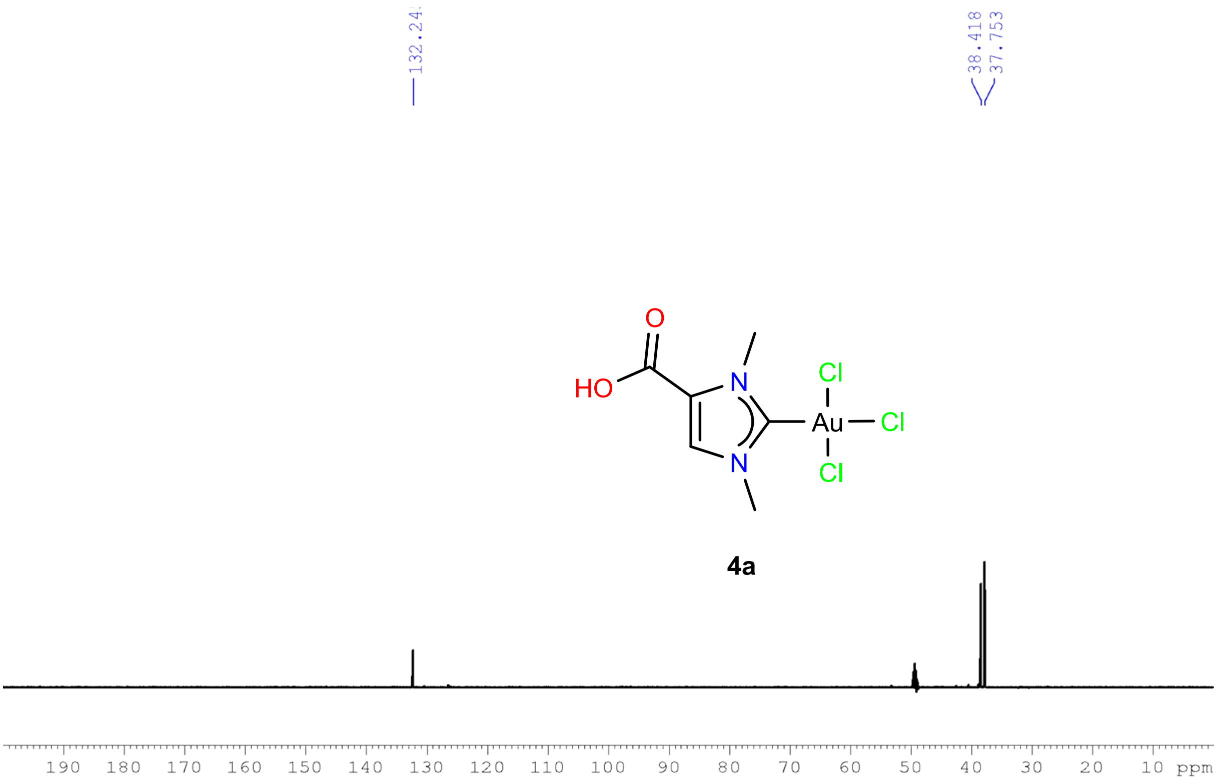

Figure S22. ^{13}C dept NMR spectrum of **4a** in MeOD at room temperature.

S1.2.8. Synthesis of compound 4b

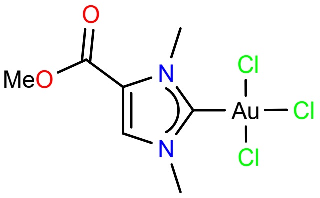

4b

Iodobenzene dichloride ($PhICl_2$, 0.14 mmol, 0.038 g, 1.4 eq.) was added to the methanol solution of compound **2b** (0.1 mmol, 0.039 g, 1 eq.). The mixture was stirred for 24 h at room temperature resulting in a light-yellow solution. The solvent was removed under vacuum. The residue was washed three times with hexane (20 mL), diethyl ether (20 mL), and cold chloroform (5 mL) to obtain complex **4b** as a white powder (0.040 g, 87%). ^{1}H NMR ($CDCl_3$, 300.1 MHz, 293 K): *δ.* 7.76 (1H, s, CH_{im}), 4.26 (3H, s, CH_3), 4.03 (3H, s, CH_3), 3.95 (3H, s, CH_3). $^{13}C\{^1H\}$ NMR ($CDCl_3$, 75.5 MHz, 293 K): *δ.* 157.7 (*C*OO), 148.9 (*C*Au), 129.8 (N*C*HC), 126.4 (N*C*COO), 53.0 (O*C*H_3), 38.6 (N*C*H_3), 37.7 (N*C*H_3). Elemental analysis calculated (%) for $C_7H_{10}AuCl_3N_2O_2$: C, 18.38, H, 2.20, N, 6.12; found: C, 18.27, H, 2.01, N, 5.93. ESI-MS m/*z* anion: calculated for $[M + Na]^+$ 478.94; Found $[M + Na]^+$478.84 m/z and calculated for $[M – CH_3]^-$ 370.99; Found $[M – CH_3]^-$ 370.68 m/z.

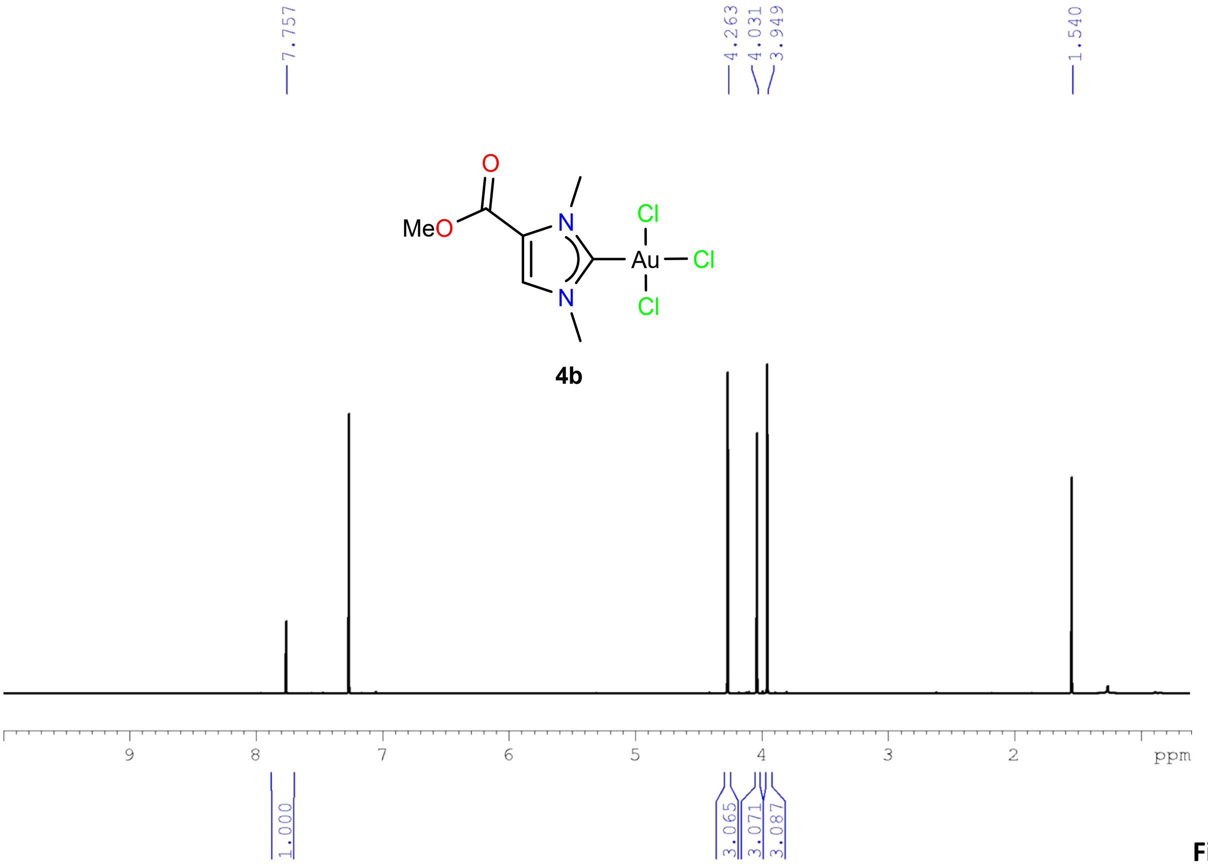

Fi

gure S23. ^{1}H NMR spectrum of **4b** in $CDCl_3$ at room temperature.

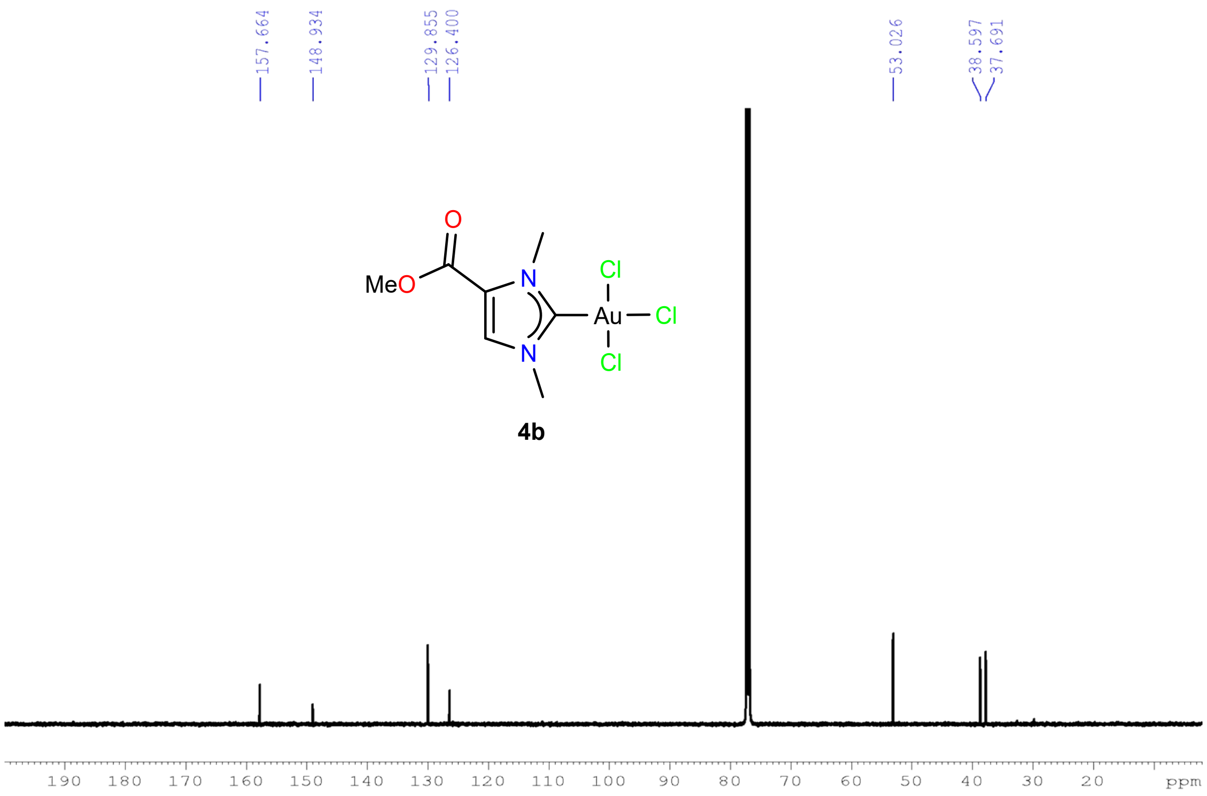

Figure S24. $^{13}C\{^{1}H\}$ NMR spectrum of **4b** in $CDCl_3$ at room temperature.

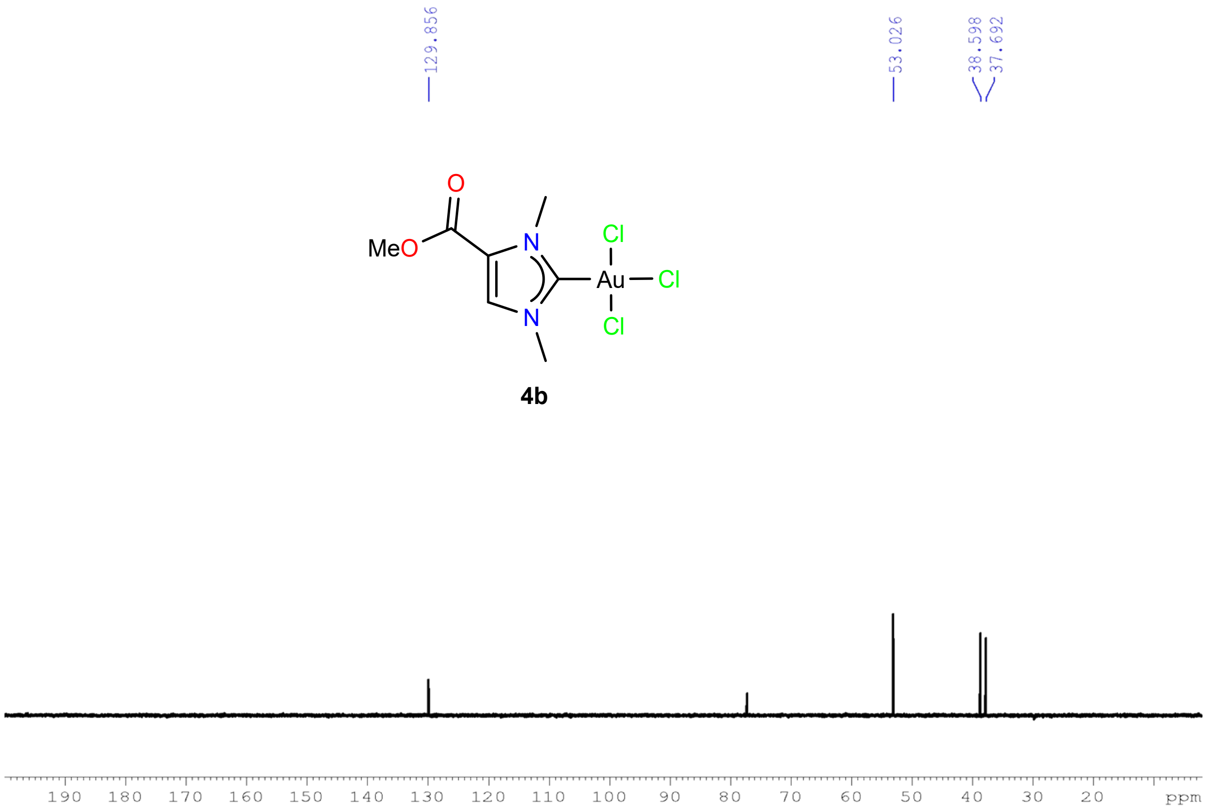

Figure S25. ^{13}C dept NMR spectrum of **4b** in $CDCl_3$ at room temperature.

S1.2.9. Synthesis of compound 4c

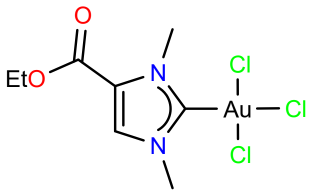

4c

Iodobenzene dichloride ($PhICl_2$, 0.038 g, 0.14 mmol, 1.4 eq.) was added to a methanol solution of compound **2c** (0.040 g, 0.1 mmol, 1 eq.). After stirring the mixture for 24 hours at room temperature, a light-yellow solution was obtained. The solvent was removed under vacuum. Three portions of hexane (20 mL) and cold diethyl ether (10 mL) were applied to the residue to obtain complex **4c** as a white powder (0.041 g, 89%). ^1H NMR ($CDCl_3$, 300.1 MHz, 293 K): *δ.* 7.78 (1H, s, CH_{imid}), 4.42 (2H, q, $^3J_{HH}$= 7.2 Hz, CH_2CH_3), 4.25 (3H, s, CH_3), 4.03 (3H, s, CH_3), 1.39 (3H, t, $^3J_{HH}$= 7.1 Hz, CH_2CH_3). $^{13}C\{^1H\}$ NMR ($CDCl_3$, 75.5 MHz, 293 K): *δ.* 157.2 (*C*OO), 148,3 (*C*Au), 129.8 (N*C*HC), 126.6 (N*C*COO), 62.5 (CH_2CH_3), 38.6 (NCH_3), 37.7 (NCH_3), 14.2 (CH_2CH_3). Elemental analysis calculated (%) for $C_8H_{12}AuCl_3N_2O_2$: C, 20.38, H, 2.57, N, 5.94; found: C, 20.49, H, 2.34, N, 5.61. ESI-MS m/*z* anion: calculated for $[M - H]^-$ 468.95; Found $[M - H]^-$ 469.02 m/z.

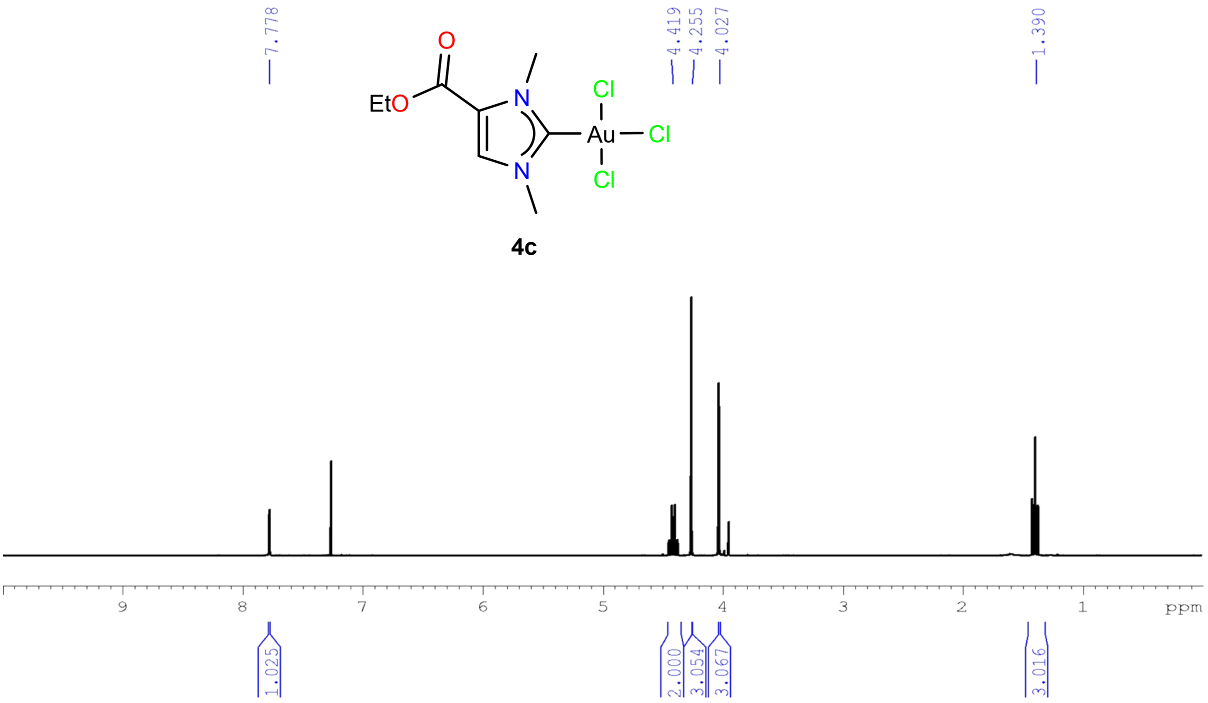

Figure S26. ^1H NMR spectrum of **4c** in $CDCl_3$ at room temperature.

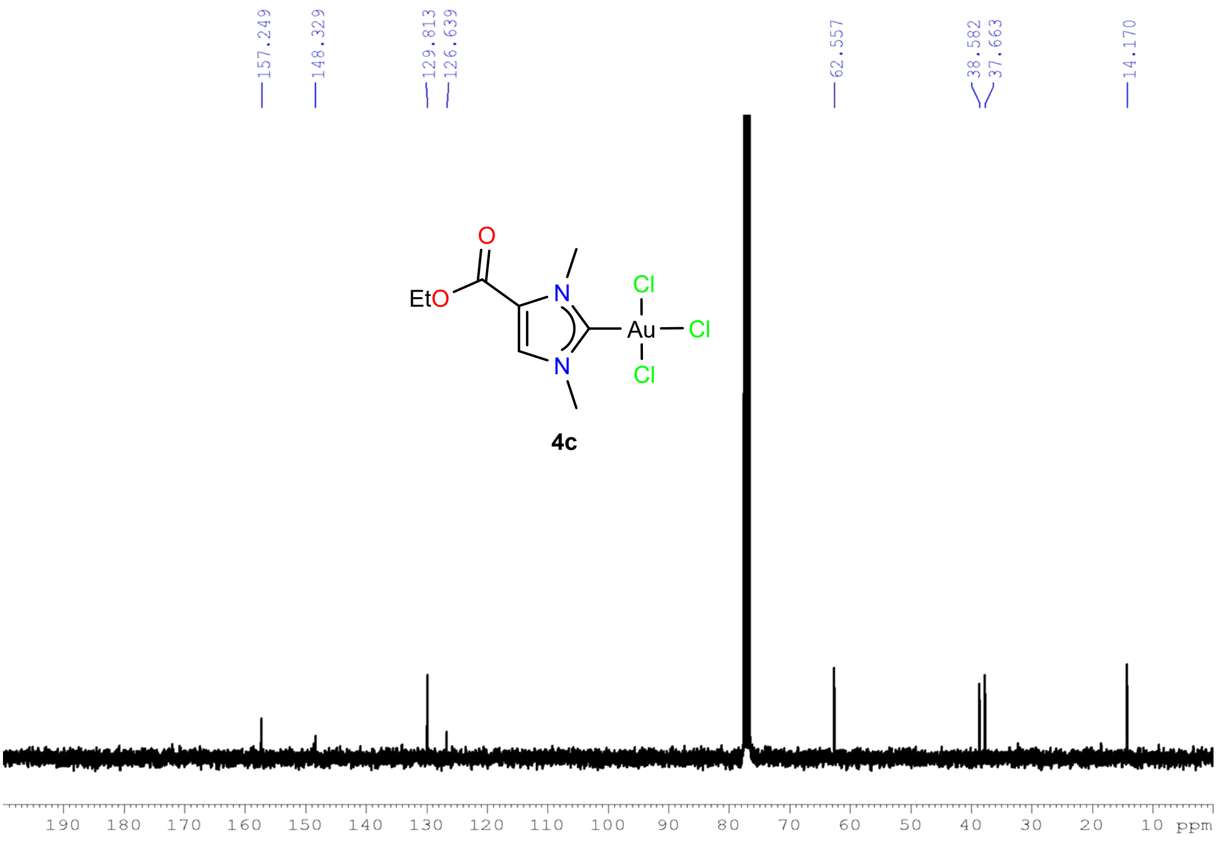

Figure S27. $^{13}C\{^{1}H\}$ NMR spectrum of **4c** in $CDCl_3$ at room temperature.

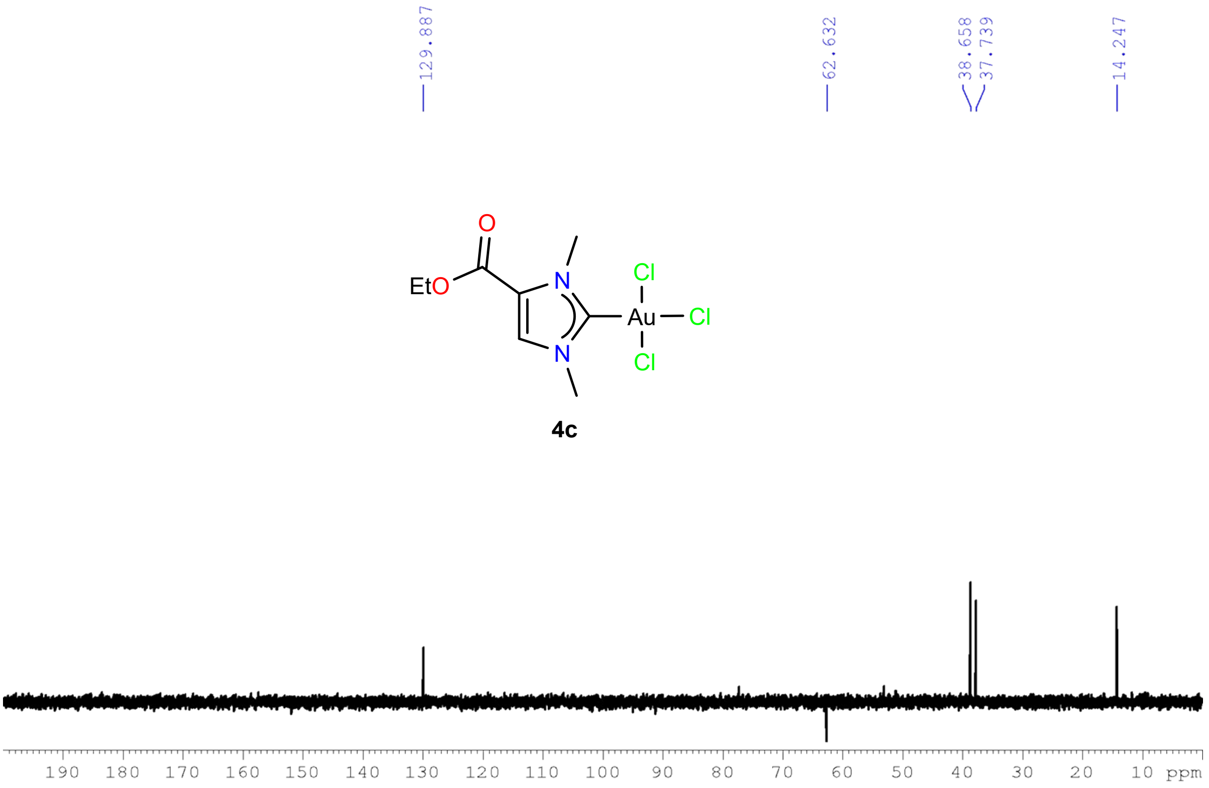

Figure S28. ^{13}C dept NMR spectrum of **4c** in $CDCl_3$ at room temperature.

S1.2.10. Synthesis of compound 5a

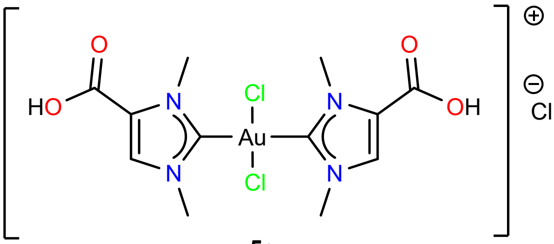

5a

Compound **3a** (0.051 g, 0.1 mmol, 1 eq.) was dissolved in a mixture of MeOH and DCM (1:1), then iodobenzene dichloride (0.027 g, 0.1 mmol, 1 eq.) was added and the mixture was stirred at room temperature for 24 hours. The solvent was removed in vacuum, and the residue was washed three times with hexane (20 mL) and diethyl ether (20 mL) and cold chloroform (5 mL) to afford complex **5a** as a white powder (0.041 g, 70%). ^{1}H NMR (CD_3OD, 300.1 MHz, 293 K): *δ.* 7.96 (1H, s, CH_{im}), 4.27 (3H, s, CH_3), 4.04 (3H, s, CH_3). $^{13}C\{^{1}H\}$ NMR (CD_3OD, 75.5 MHz, 293 K): *δ.* 157.9 (*C*Au), 130.3 (N*C*HC), 37.9 (N*C*H_3), 37.4 (N*C*H_3). due to low solubility ^{13}C resonances for (*C*OO) and (N*C*COO) were not observed. Elemental analysis calculated (%) for $C_{12}H_{16}AuCl_3F_3N_4O_4$: C, 24.70, H, 2.76, N, 9.60; found: C, 24.13, H, 2.48, N, 9.35. ESI-MS *m/z* anion: calculated for $[M]^+$ 547.02; Found $[M]^+$ 547.00 m/z.

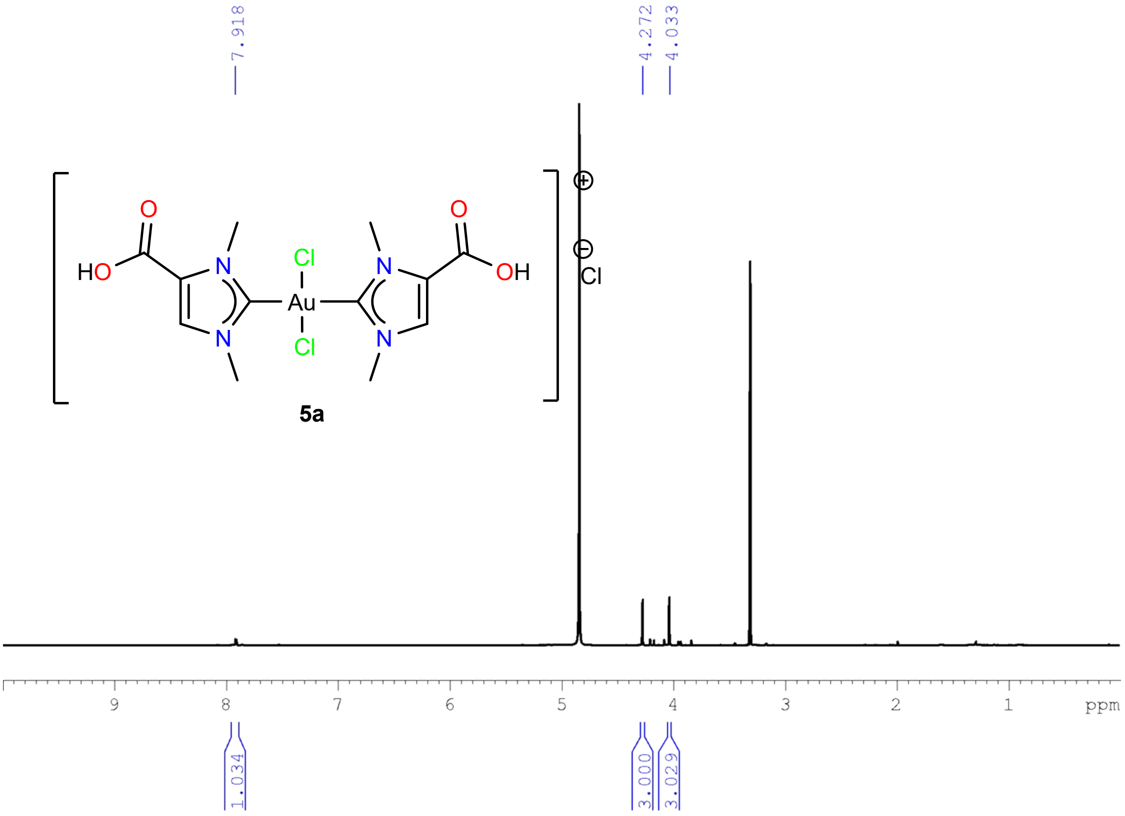

Figure S29. ^{1}H NMR spectrum of **5a** in CD_3OD at room temperature.

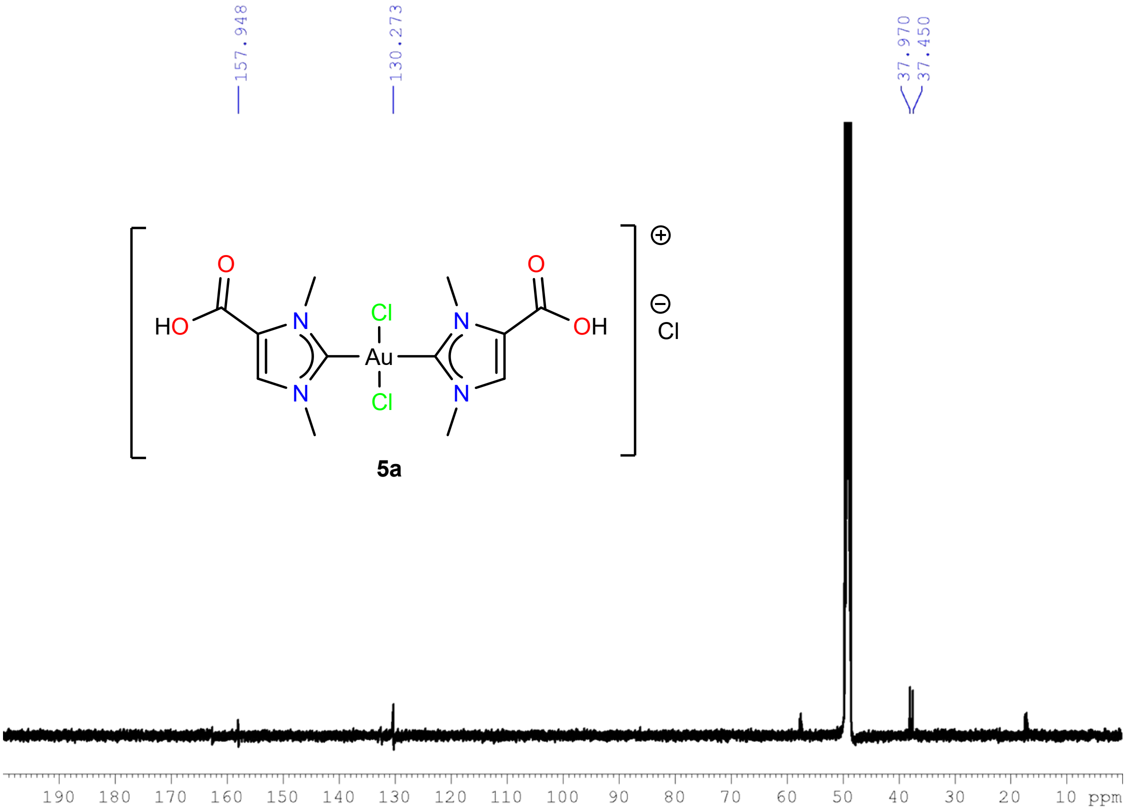

Figure S30. $^{13}C\{^{1}H\}$ NMR spectrum of **5a** in CD_3OD at room temperature.

S1.2.11. Synthesis of compound 5b

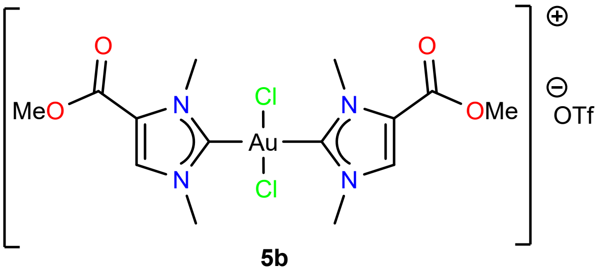

5b

The mixture of compound **3b** (0.131 g, 0.2 mmol, 1 eq.) and iodobenzene dichloride ($PhICl_2$, 0.055 g, 0.2 mmol, 1 eq.) in dichloromethane was stirred for 24 h at room temperature, resulting in a light-yellow solution. After removing the solvent, a yellowish solid was obtained. It was washed three times with hexane (20 mL) followed by diethyl ether (20 mL) and once with cold methanol (5 mL) to obtain complex **5b** as a white powder. A flash chromatography for further purification (93% DCM: 7% MeOH) to afford **5b** (0.103 g, 71%). ^{1}H NMR ($CDCl_3$, 300.1 MHz, 293 K): *δ.* 8.17 (1H, s, CH_{im}), 4.24 (3H, s, CH_3), 4.12 (3H, s, CH_3), 3.92 (3H, s, CH_3). $^{13}C\{^1H\}$ NMR ($CDCl_3$, 75.5 MHz, 293 K): *δ.* 158.4 (*C*OO), 158.3 (*C*Au), 131.6 (N*C*HC), 125.9 (N*C*COO), 120.9 (q, $^1J_{C,F}$= 320 MHz, *C*F_3), 52.9 (O*C*H_3), 38.6 (N*C*H_3), 37.4 (N*C*H_3). Elemental analysis calculated (%) for $C_{15}H_{20}AuCl_2F_3N_4O_7S$: C, 24.84, H, 2.78, N, 7.73; found: C, 24.37, H, 2.77, N, 7.42. ESI-MS m/*z* anion: calculated for $[M]^+$ 575.05; Found $[M]^+$ 575.04 m/z.

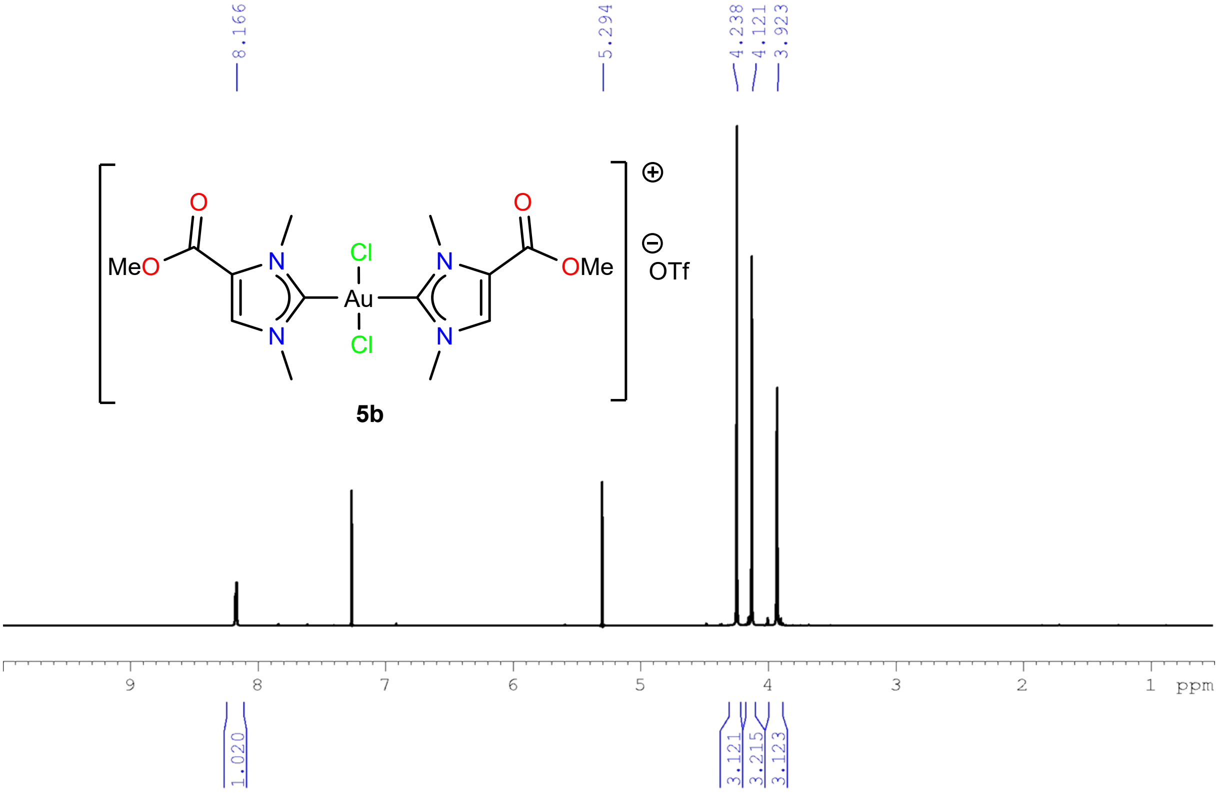

Figure S31. ^{1}H NMR spectrum of **5b** in $CDCl_3$ at room temperature.

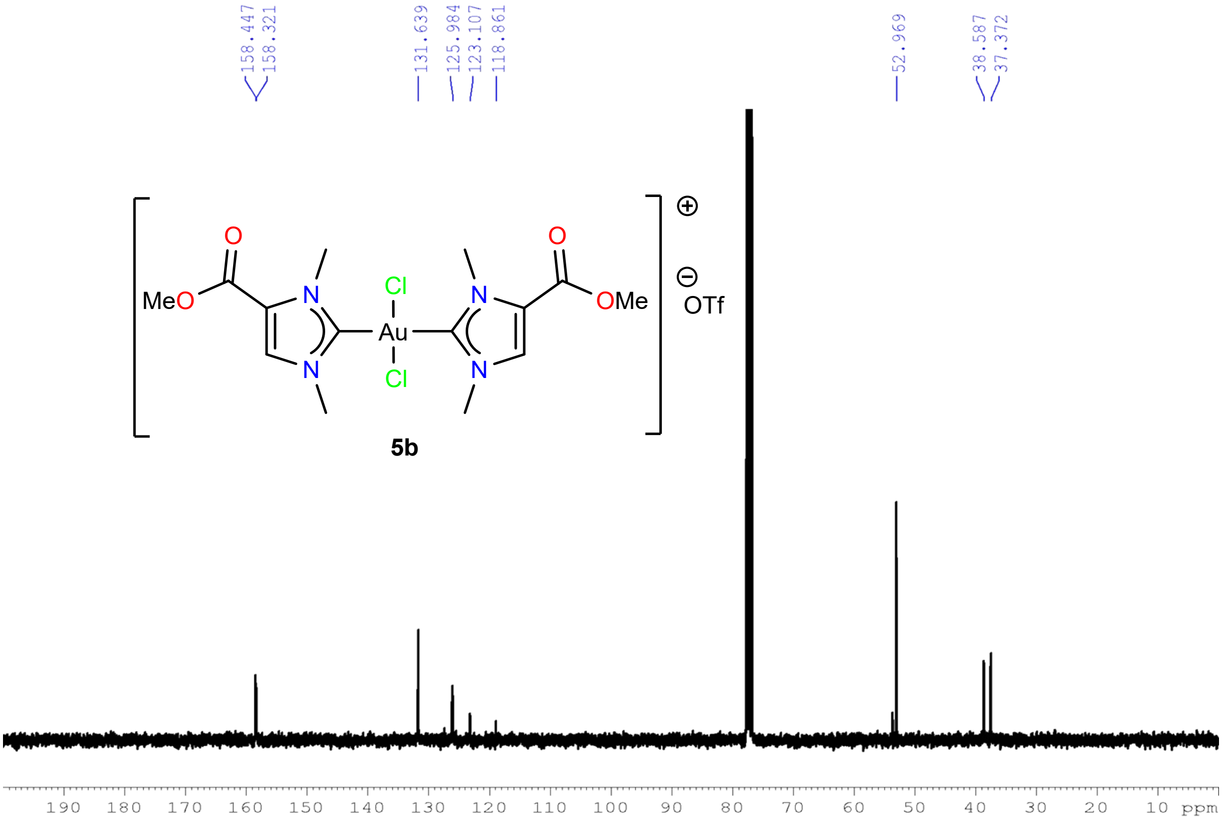

Figure S32. $^{13}C\{^{1}H\}$ NMR spectrum of **5b** in $CDCl_3$ at room temperature.

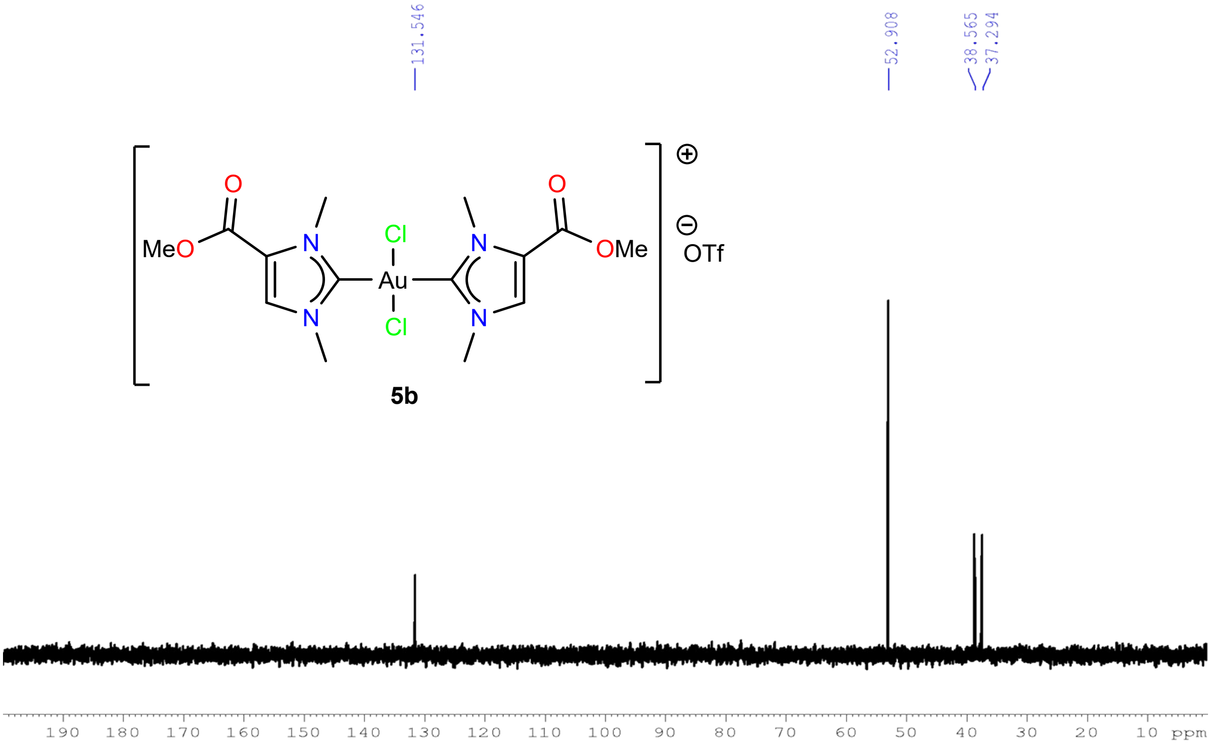

Figure S33. ^{13}C dept NMR spectrum of **5b** in $CDCl_3$ at room temperature.

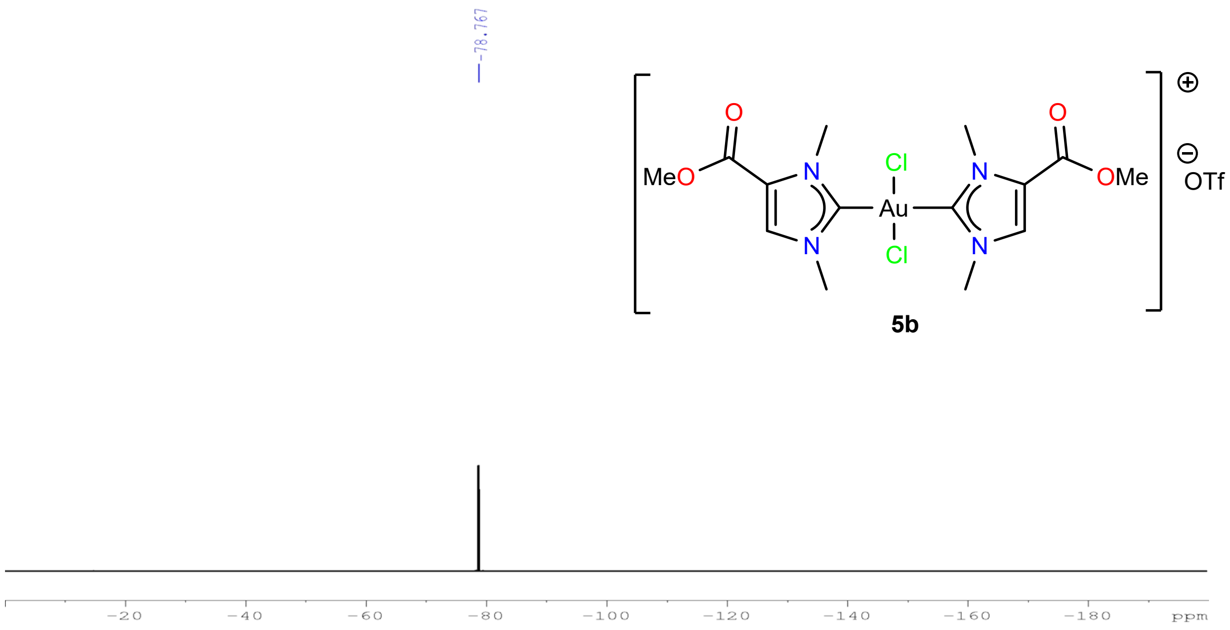

Figure S34. ^{19}F NMR spectrum of **5b** in $CDCl_3$ at room temperature.

S1.2.12. Synthesis of compound 5c

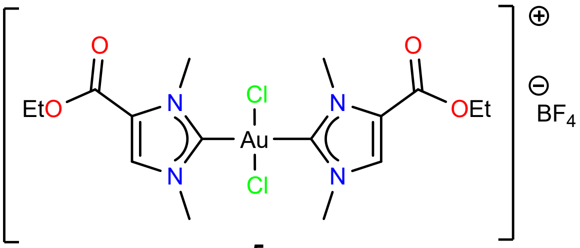

5c

The mixture of compound **3c** (0.62 g, 0.1 mmol, 1 eq.) and iodobenzene dichloride ($PhICl_2$, 0.027 g, 0.1 mmol, 1 eq.) in dichloromethane was stirred for 24 h at room temperature resulting in a light-yellow solution. The solvent was removed in vacuum to obtain a yellowish solid. The residue was washed three times with hexane (20 mL), twice with diethyl ether (20 mL), and once with cold methanol (5 mL) to obtain complex **5c**. Further purificatin by flash chromatography using silica 60 (90% DCM: 10% MeOH) afforded **5c** as a white solid (0.049 g, 72%). ^{1}H NMR ($CDCl_3$, 300.1 MHz, 293 K): *δ.* 8.07 (1H, s, CH_{imid}), 4.38 (2H, q, $^3J_{HH}$= 7.1 Hz, CH_2CH_3), 4.23 (3H, s, CH_3), 4.11 (3H, s, CH_3), 1.38 (3H, t, $^3J_{HH}$= 7.1 Hz, CH_2CH_3). ^{13}C{^{1}H} NMR ($CDCl_3$, 75.5 MHz, 293 K): *δ.* 158.2 (*C*OO), 157.9 (*C*Au), 131.2 (N*C*HC), 126.1 (N*C*COO), 62.3 (*C*H$_2$CH$_3$), 38.4 (N*C*H$_3$), 37.2 (N*C*H$_3$), 14.1 (*C*H$_3$CH$_2$). ^{19}F NMR ($CDCl_3$, 282.5 MHz, 293 K): *δ.* -152.7 (m,$^{11}BF_4$), -152.6 (q, $^3J_{HH}$= 1.3 MHz, $^{10}BF_4$). Elemental analysis calculated (%) for $C_{16}H_{24}AuBCl_2F_4N_4O_4$ (691.06): C, 27.81, H, 3.50, N, 8.11; found: C, 27.42, H, 3.75, N, 7.80. ESI-MS m/*z* anion: calculated for [M]$^+$ 603.08; Found [M]$^+$ 603.06 m/z.

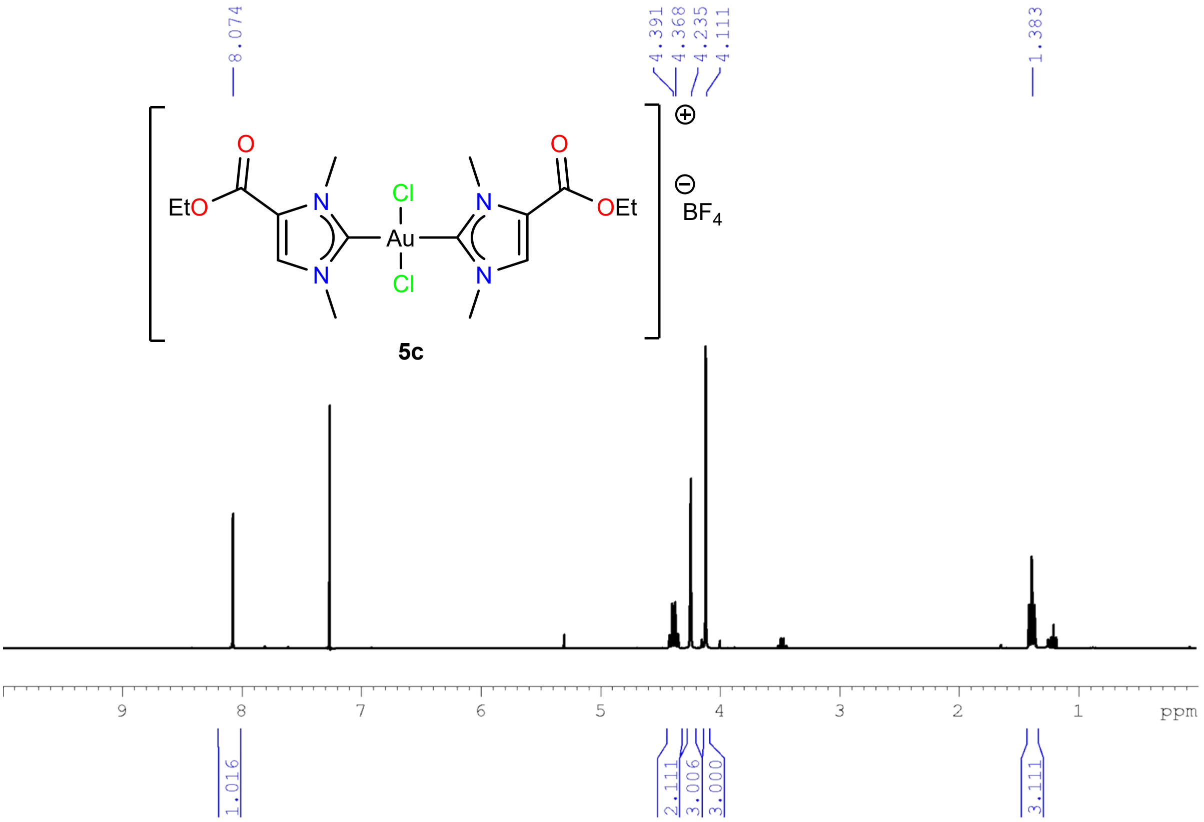

Figure S35. ^{1}H NMR spectrum of **5c** in $CDCl_3$ at room temperature.

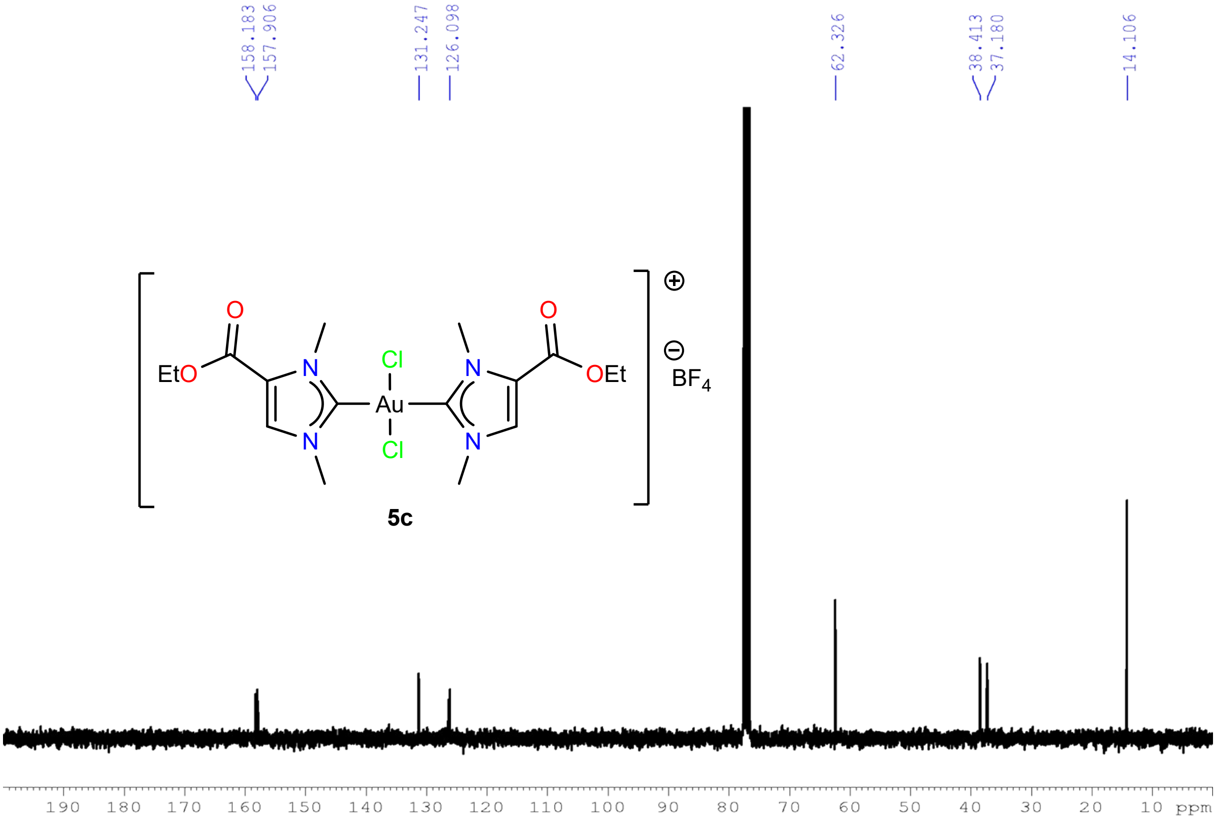

Figure S36. $^{13}C\{^{1}H\}$ NMR spectrum of **5c** in $CDCl_3$ at room temperature.

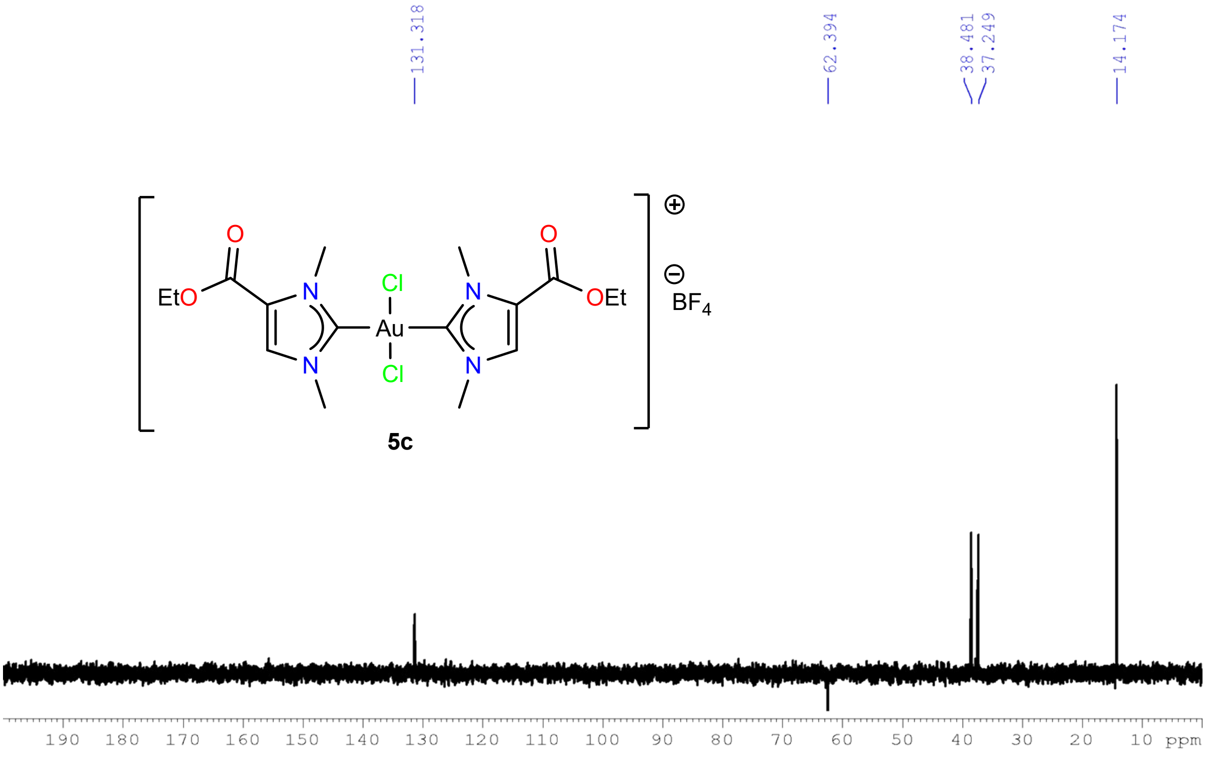

Figure S37. ^{13}C dept NMR spectrum of **5c** in $CDCl_3$ at room temperature.

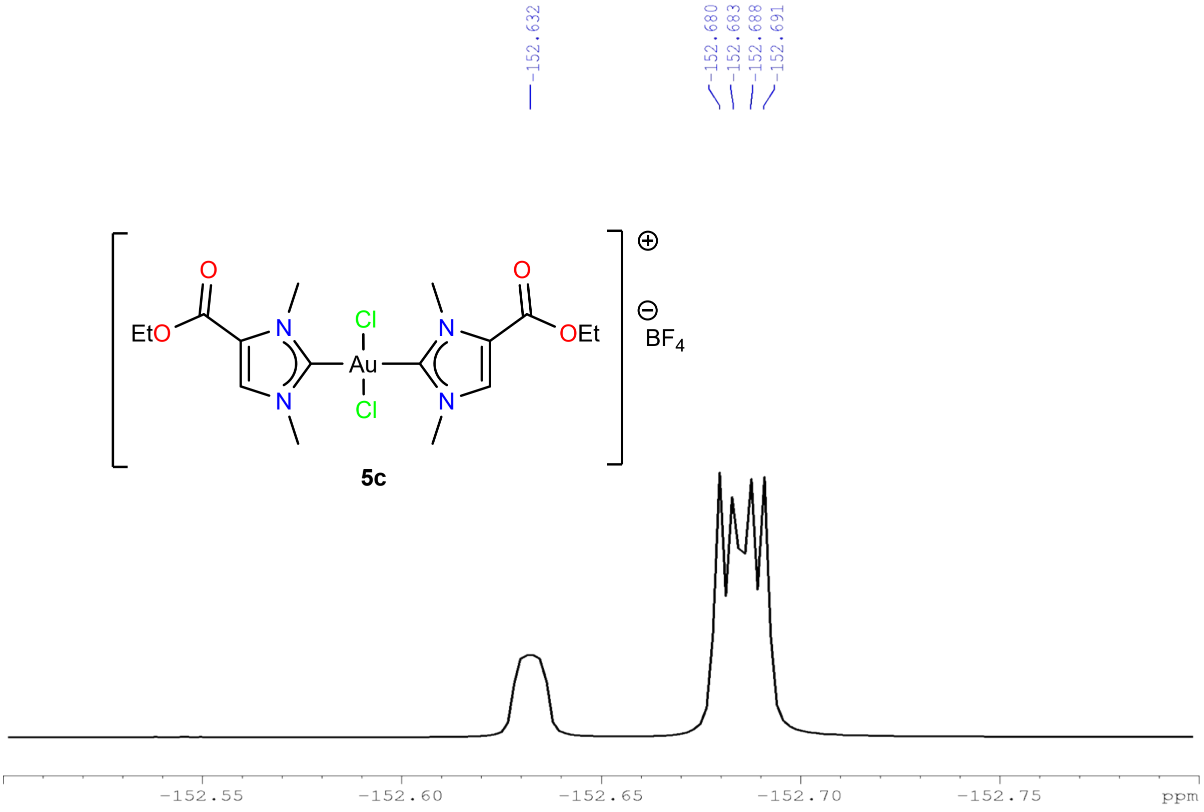

Figure S38. ^{19}F NMR spectrum of **5c** in $CDCl_3$ at room temperature.

S1.3. Single Crystal X-Ray Diffraction

Crystals were mounted on top of a human hair or on a Hampton Research CryoLoop™ with perfluorinated inert oil. Data were recorded on Rigaku XtaLAB Synergy S Single Source diffractometers equipped with a HyPix-6000HE detector and a PhotonJet microfocus source with Cu-Kα (**1c**, **3c**, **5c**) or Mo-Kα (**2b**, **1b**, **2c**, **4b**, **4c**, **4a**, **3b**, **5b**) radiation. Data reduction was performed with CrysalisPro[3]. Absorption correction was based on multi-scans for **1c** and **2b**. For all other structures additionally face indexation and integration on a Gaussian grid was applied. The structure of compound **3b** was solved with the Patterson method with SHELXS-2013/1[4] whereas all other structures were solved by intrinsic phasing with SHELXT-2018/2[5] and refinements are based on F^2 using the program SHELXL-2018/3[5] in OLEX^2 [6]. The hydrogen atoms of the OH groups in **4a** have been refined freely. All other hydrogen atoms were placed in idealized positions and refined using a riding model.

1c was refined as a non-merohedral twin with the second component rotated by 179.8790° around [0.71 0.71 -0.01] (reciprocal) or [0.69 0.71 -0.14] (direct) and the relative volume of the smaller component refined to 42.8 %.

2b was refined as a non-merohedral twin with the minor component rotated by 179.8488° around [0.16 0.77 -0.61] (reciprocal) or [0.58 0.58 -0.58] (direct). The relative volume of the smaller component refined to 2.3 %.

For the structure of **2c** the program ADDSYMM as part of the CHECKCIF[7] routine suggests a different cell setup. A significant number of reflections could not be indexed with the cell suggested by ADDSYMM (97% indexation with the given cell vs. 73 % indexation with the suggested smaller cell volume). Additionally, the two molecules are not exactly overlapping. The torsion angles C6-O2-C7-C8 f e. are 156.22° and 171.91° for the two independent molecules.

4a was refined as a non-merohedral twin with component 2 rotated by 179.9923° around [0.39 0.84 0.37] (reciprocal) or [0.00 1.00 0.00] (direct). The relative volume of the smaller component refined to 48.9 %.

3b was refined as a pseudo merohedral twin (with twin matrix 1 0 0 / 0 -1 0 / -1 0 -1) and the relative volume of the smaller component refined to 33.7 %.

3c was refined as a non-merohedral 3-component twin with component 2 rotated by 6.0221° around [0.83 0.37 0.41] (reciprocal) or [1.00 0.05 0.02] (direct) and component 3 rotated by 1.8219° around [-0.18 -0.97 0.15] (reciprocal) or [-0.10 -0.99 0.12] (direct). The relative volume of the smaller components refined to 28.9 % and 30.2 % respectively. Additionally, the BF_4 anion was found disordered and refined accordingly. This structure suffers from low bond length precision and exhibits high R values. Despite several attempts only weakly diffracting crystals of this compound were obtained. This structure only confirms the connectivity of the compound and any geometrical parameters should be interpreted with caution.

5b was refined as a non-merohedral twin with the second component rotated by -179.9751° around [0.62 -0.78 -0.07] (reciprocal) or [0.71 -0.71 -0.00] (direct). The relative volume of the smaller component refined to 27.9 %.

Two BF_4 anions in **5c** were found disordered and refined accordingly. Chemically equivalent B-F and F-F distances were restrained to be equal.

Table S1. Crystal data and structure refinement of **1b**.

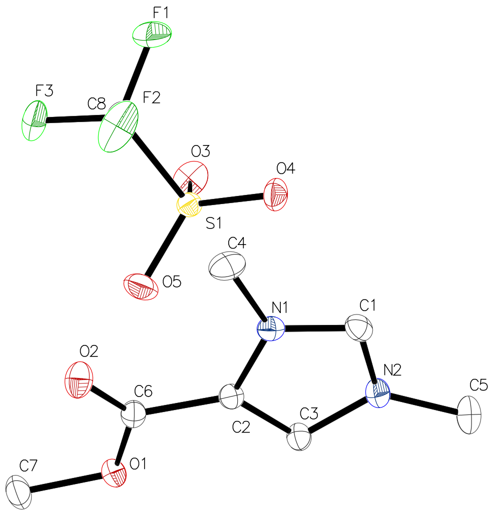

	CCDC	2355435
	Empirical formula	$C_8H_{11}F_3N_2O_5S$
	Formula weight	304.25
	Temperature	100(2) K
	Wavelength	0.71073 Å
	Crystal system	Orthorhombic
	Space group	$P2_12_12_1$
	Unit cell dimensions	
	a = 8.7426(3) Å	α = 90°
	b = 8.8249(3) Å	β = 90°
	c = 15.7395(5) Å	γ = 90°
Volume	1214.34(7) Å^3	
Z	4	
Density (calculated)	1.664 Mg/m^3	
Absorption coefficient	0.325 mm^{-1}	
F(000)	624	
Crystal habitus	irregular (colourless)	
Crystal size	0.408 x 0.287 x 0.091 mm^3	
ϑ range for data collection	3.280 to 41.005°	
Index ranges	$-16 \le h \le 15$, $-15 \le k \le 16$, $-29 \le l \le 28$	
Reflections collected	38656	
Independent reflections	7712[R_{int} = 0.0225]	
Completeness to ϑ = 25.242°	99.8 %	
Max. and min. transmission	1.000 and 0.318	
Data / restraints / parameters	7712 / 0 / 175	
Goodness-of-fit on F^2	1.035	
Final R indices [$I > 2\sigma(I)$]	R_1 = 0.0266, wR_2 = 0.0695	
R indices (all data)	R_1 = 0.0304, wR_2 = 0.0711	
Absolute structure parameter	0.027(12)	
Largest diff. peak and hole	0.440 and -0.197 e·Å^3	
Crystallisation Details:	from chloroform / n-hexane	

Thermal ellipsoids are presented at the 50 % level of probability. Bond distances and bond angles are reported in Å or degree (°), respectively. N1–C1 1.3238(12), N1–C2 1.3861(11), N1–C4 1.4657(12), N2–C1 1.3369(12), N2–C3 1.3712(11), O1–C6 1.3287(10), O1–C7 1.4460(12), O2–C6 1.2107(11), S1–O4 1.4470(7), S1–C8 1.8257(9), F1–C8 1.3424(12), N1–C1–N2 109.21(7), C3–C2–N1 107.11(7), C1–N1–C2 108.15(7), C1–N1–C4 123.40(8), C1–N2–C3 108.65(7), O2–C6–O1 125.34(8), C6–O1–C7 115.35(8).

Table S2. Crystal data and structure refinement of **1c**.

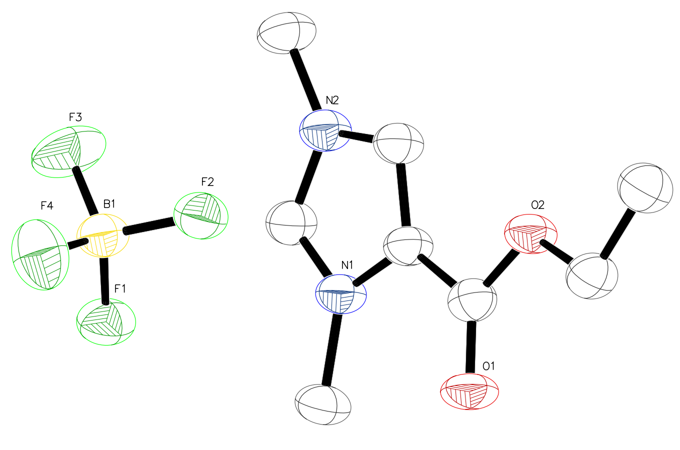	CCDC	2355436
	Empirical formula	$C_8H_{13}BF_4N_2O_2$
	Formula weight	256.01
	Temperature	100(2) K
	Wavelength	1.54184 Å
	Crystal system	Triclinic
	Space group	$P\bar{1}$
	Unit cell dimensions	
	a = 8.1082(7) Å	α = 79.200(10)°
	b = 8.1979(10) Å	β = 88.373(8)°
	c = 9.6164(11) Å	γ = 66.083(10)°
Volume	573.16(12) Å^3	
Z	2	
Density (calculated)	1.483 Mg/m^3	
Absorption coefficient	1.296 mm^{-1}	
F(000)	264	
Crystal habitus	irregular (colourless)	
Crystal size	0.07 x 0.05 x 0.010 mm^3	
ϑ range for data collection	4.688 to 66.596°	
Index ranges	$-9 \leq h \leq 9$, $-9 \leq k \leq 9$, $-11 \leq l \leq 11$	
Reflections collected	6548	
Independent reflections	6548[R_{int} = ?]	
Completeness to ϑ = 66.596°	99.8 %	
Max. and min. transmission	1.00000 and 0.49233	
Data / restraints / parameters	6548 / 0 / 158	
Goodness-of-fit on F^2	1.102	
Final R indices [$I > 2\sigma(I)$]	R_1 = 0.1020, wR_2 = 0.2657	
R indices (all data)	R_1 = 0.1366, wR_2 = 0.2979	
Largest diff. peak and hole	0.579 and -0.328 e·Å^3	
Crystallisation Details:	from DCM / n-hexane, r.t., gas diffusion	

Thermal ellipsoids are presented at the 50 % level of probability. Bond distances and bond angles are reported in Å or degree (°), respectively. N1–C1 1.316(7), N1–C2 1.382(7), N1–C4 1.472(7), N2–C1 1.336(7), N2–C3 1.372(7), N2–C5 1.463(7), C2–C3 1.362(7), C2–C6 1.470(7), O1–C6 1.212(6), O2–C7 1.464(6), O2–C6 1.331(7), F1–B1 1.404(7), N1–C1–N2 109.7(5), C3–C2–N1 107.0(4), C1–N1–C2 108.2(4), C1–N1–C4 123.7(4), C1–N2–C3 108.1(4), O1–C6–O2 125.4(5), C6–O2–C7 116.2(4), O2–C6–C2 110.4(4).

Table S3. Crystal data and structure refinement of **2b**.

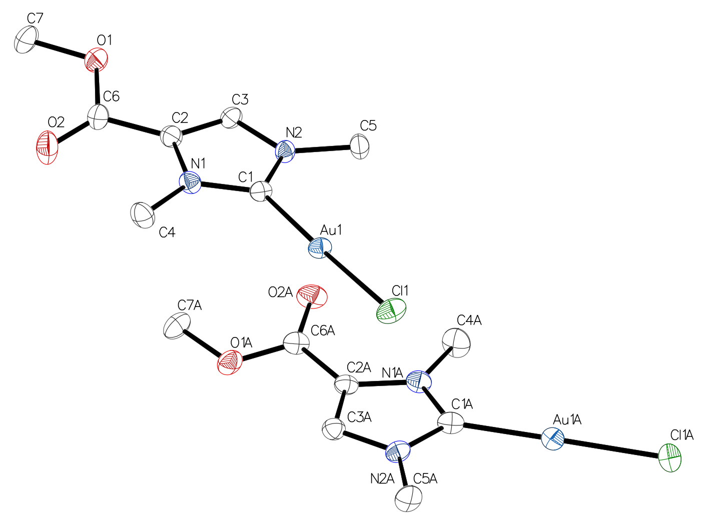

	CCDC	2355437
	Empirical formula	$C_7H_{10}AuClN_2O_2$
	Formula weight	386.59
	Temperature	109.5(4) K
	Wavelength	0.71073 Å
	Crystal system	Monoclinic
	Space group	$P2_1/n$
	Unit cell dimensions	
	a = 7.68619(11) Å	α = 90°
	b = 17.2395(3) Å	β = 92.2421(14)°
	c = 15.2298(2) Å	γ = 90°
Volume	2016.50(5) Å^3	
Z	8	
Density (calculated)	2.547 Mg/m^3	
Absorption coefficient	14.825 mm^{-1}	
F(000)	1424	
Crystal habitus	irregular (colourless)	
Crystal size	0.13 x 0.11 x 0.09 mm^3	
ϑ range for data collection	2.677 to 31.328°	
Index ranges	$-11 \le h \le 11$, $-24 \le k \le 24$, $-22 \le l \le 22$	
Reflections collected	14688	
Independent reflections	14688[R_{int} = ?]	
Completeness to ϑ = 25.242°	99.9 %	
Max. and min. transmission	1.00000 and 0.68399	
Data / restraints / parameters	14688 / 0 / 242	
Goodness-of-fit on F^2	1.066	
Final R indices [$I > 2\sigma(I)$]	R_1 = 0.0270, wR_2 = 0.0609	
R indices (all data)	R_1 = 0.0403, wR_2 = 0.0641	
Largest diff. peak and hole	1.941 and -1.973 e·Å^3	
Crystallisation Details:	from $CHCl_3$ / n-hexane,	

Thermal ellipsoids are presented at the 50 % level of probability. Bond distances and bond angles are reported in Å or degree (°), respectively. N1–C1 1.354(6), N1–C2 1.390(6), Au1–Cl1 2.2844(6), Au1–C1 1.987(2), N1–C4 1.466(6), N2–C1 1.362(5), N2–C3 1.366(5), N2–C5 1.467(5), C6–C2 1.469(6), C2–C3 1.356(6), O1–C6 1.341(6), O1–C7 1.451(6), O2–C6 1.203(6), N1–C1–N2 105.2(4), C3–C2–N1 107.2(4), C1–N1–C2 109.8(4), C1–N1–C4 123.8(4), C1–N2–C3 111.1(4), O1–C6–O2 124.6(4), C6–O1–C7 114.9(4), O1–C6–C2 110.5(4), N1–C1–Au1 129.3(3), C1–Au1–Cl1 178.05(13).

Table S4. Crystal data and structure refinement of **2c**.

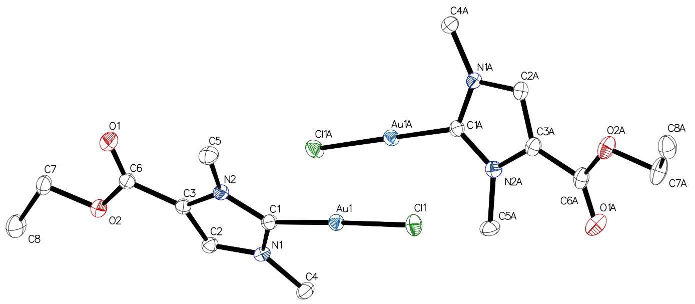	CCDC	2355438
	Empirical formula	$C_8H_{12}AuClN_2O_2$
	Formula weight	400.61
	Temperature	100(2) K
	Wavelength	0.71073 Å
	Crystal system	Monoclinic
	Space group	$P2_1/c$
	Unit cell dimensions	
	a = 17.5275(4) Å	α = 90°
	b = 7.06271(17) Å	β = 96.314(2)°
	c = 17.9398(4) Å	γ = 90°
Volume	2207.33(9) Å^3	
Z	8	
Density (calculated)	2.411 Mg/m^3	
Absorption coefficient	13.548 mm^{-1}	
F(000)	1488	
Crystal habitus	irregular (colourless)	
Crystal size	0.100 x 0.090 x 0.040 mm^3	
ϑ range for data collection	2.284 to 38.264°	
Index ranges	$-30 \leq h \leq 30$, $-11 \leq k \leq 12$, $-30 \leq l \leq 30$	
Reflections collected	114486	
Independent reflections	11729[R_{int} = 0.0404]	
Completeness to ϑ = 25.242°	100.0 %	
Max. and min. transmission	0.794 and 0.413	
Data / restraints / parameters	11729 / 0 / 259	
Goodness-of-fit on F^2	1.022	
Final R indices [$I > 2\sigma(I)$]	R_1 = 0.0262, wR_2 = 0.0435	
R indices (all data)	R_1 = 0.0405, wR_2 = 0.0460	
Largest diff. peak and hole	1.755 and -1.499 e·Å^3	
Crystallisation Details:	from $CHCl_3$ / *n*-pentane	

Thermal ellipsoids are presented at the 50 % level of probability. Bond distances and bond angles are reported in Å or degree (°), respectively. N1–C1 1.347(3), N1–C2 1.366(3), Au1–Cl1 2.2873(5), Au1–C1 1.984(2), N2–C3 1.392(3), N2–C5 1.459(3), C2–C3 1.365(3), C3–C6 1.464(3), O1–C6 1.205(3), O2–C7 1.453(3), O2–C6 1.341(3), N1–C1–N2 105.84(17), C3–C2–N1 106.89(18), C1–N1–C2 111.04(17), C1–N1–C4 124.31(17), C1–N2–C3 109.81(17), O1–C6–O2 124.5(2), C6–O2–C7 115.91(17), O2–C6–C3 109.73(18), N1–C1–Au1 126.52(15), C1–Au1–Cl1 178.42(6).

Table S5. Crystal data and structure refinement of **3b**.

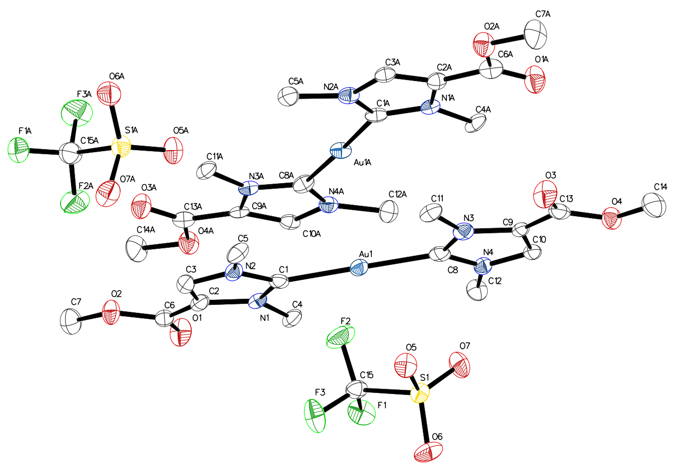

CCDC	2355439
Empirical formula	$C_{15}H_{20}AuF_3N_4O_7S$
Formula weight	654.38
Temperature	100(2) K
Wavelength	0.71073 Å
Crystal system	Monoclinic
Space group	$P2_1/c$
Unit cell dimensions	
Volume	1132.52(4) Å^3
Z	4
Density (calculated)	2.601 Mg/m^3

Volume	4248.4(4) Å^3
Z	8
Density (calculated)	2.046 Mg/m^3
Absorption coefficient	7.095 mm^{-1}
F(000)	2528
Crystal habitus	plate (colourless)
Crystal size	0.260 x 0.180 x 0.030 mm^3
ϑ range for data collection	1.974 to 33.138°
Index ranges	$-31 \leq h \leq 31$, $-10 \leq k \leq 10$, $-47 \leq l \leq 48$
Reflections collected	147355
Independent reflections	16356[R_{int} = 0.0798]
Completeness to ϑ = 25.242°	100.0 %
Max. and min. transmission	1.000 and 0.267
Data / restraints / parameters	16356 / 0 / 572
Goodness-of-fit on F^2	1.049
Final *R* indices [$I > 2\sigma(I)$]	R_1 = 0.0618, wR_2 = 0.1143
R indices (all data)	R_1 = 0.0848, wR_2 = 0.1211
Largest diff. peak and hole	4.018 and -5.653 e·Å^3
Crystallisation Details:	$CHCl_3$ / *n*-pentane

Thermal ellipsoids are presented at the 50 % level of probability. Bond distances and bond angles are reported in Å or degree (°), respectively. N1–C1 1.357(11), N1–C4 1.471(11), Au1–C1 2.014(9), N2–C1 1.363(11), N2–C5 1.475(12), O1–C6 1.204(12), O2–C7 1.452(13), O2–C6 1.338(12), N1–C1–N2 104.4(7), C3–C2–N1 106.9(8), C1–N1–C2 110.2(7), C1–N1–C4 122.9(7), O1–C6–O2 125.0(9), C6–O2–C7 116.5(9), N1–C1–Au1 127.1(6), C1–Au1–C8 178.7(4).

Table S6. Crystal data and structure refinement of **3c**.

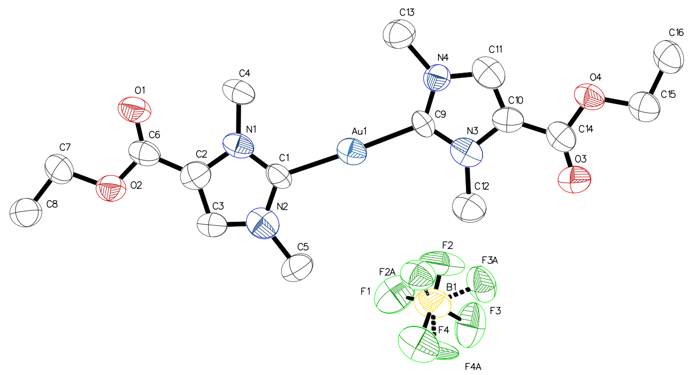

	CCDC	2355440
	Empirical formula	$C_{16}H_{24}AuBF_4N_4O_4$
	Formula weight	620.17
	Temperature	100(2) K
	Wavelength	1.54184 Å
	Crystal system	Triclinic
	Space group	$P\bar{1}$
	Unit cell dimensions	
	a = 7.0200(6) Å	α = 86.570(8)°
	b = 10.3525(10) Å	β = 79.101(9)°
	c = 15.2507(17) Å	γ = 76.542(8)°
Volume	1058.32(19) $Å^3$	
Z	2	
Density (calculated)	1.946 Mg/m^3	
Absorption coefficient	13.662 mm^{-1}	
F(000)	600	
Crystal habitus	plate (colourless)	
Crystal size	0.160 x 0.120 x 0.020 mm^3	
ϑ range for data collection	2.951 to 75.012°	
Index ranges	$-8 \le h \le 8$, $-12 \le k \le 12$, $-19 \le l \le 19$	
Reflections collected	10988	
Independent reflections	10988[R_{int} = ?]	
Completeness to ϑ = 67.684°	98.9 %	
Max. and min. transmission	1.000 and 0.246	
Data / restraints / parameters	10988 / 0 / 307	
Goodness-of-fit on F^2	1.613	
Final R indices [$I > 2\sigma(I)$]	R_1 = 0.1326, wR_2 = 0.3468	
R indices (all data)	R_1 = 0.1674, wR_2 = 0.3902	
Largest diff. peak and hole	7.325 and -3.611 e·$Å^3$	
Crystallisation Details:	CH_2Cl_2 / n-pentane, r.t.	

Thermal ellipsoids are presented at the 50 % level of probability. Bond distances and bond angles are reported in Å or degree (°), respectively. N1–C1 1.34(3), N1–C4 1.43(3), Au1–C1 2.05(2), N2–C1 1.33(3), N2–C5 1.48(4), O1–C6 1.20(3), O2–C6 1.34(3), O2–C7 1.47(3), N2–C1–N1 108(2), C3–C2–N1 107(2), C1–N1–C2 108(2), C1–N1–C4 126(2), O1–C6–O2 125(3), C6–O2–C7 114(2), N1–C1–Au1 124.1(18), C9–Au1–C1 177.5(9).

Table S7. Crystal data and structure refinement of **4a**.

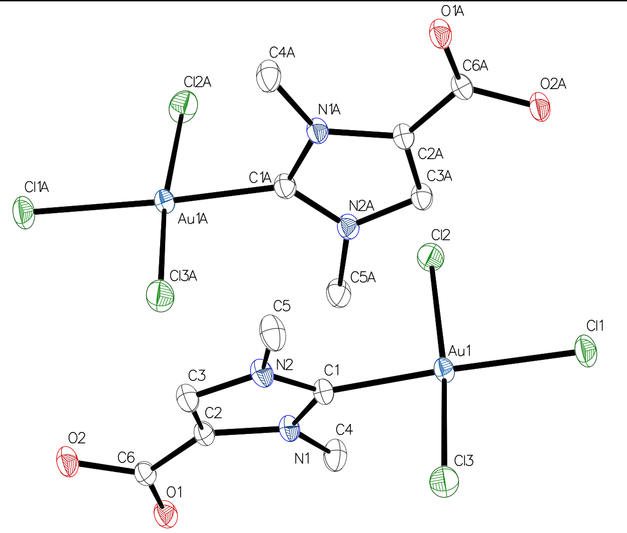

CCDC	2355441
Empirical formula	$C_6H_8AuCl_3N_2O_2$
Formula weight	443.46
Temperature	100(2) K
Wavelength	0.71073 Å
Crystal system	Triclinic
Space group	$P\bar{1}$
Unit cell dimensions	
a = 10.7651(2) Å	α = 66.2328(18)°
b = 10.9548(2) Å	β = 83.8452(16)°
c = 11.9269(2) Å	γ = 62.158(2)°
Volume	1132.52(4) Å^3
Z	4
Density (calculated)	2.601 Mg/m^3
Absorption coefficient	13.673 mm^{-1}
F(000)	816
Crystal habitus	irregular (colourless)
Crystal size	0.240 x 0.150 x 0.080 mm^3
ϑ range for data collection	2.150 to 45.023°
Index ranges	$-21 \le h \le 21$, $-21 \le k \le 21$, $-23 \le l \le 23$
Reflections collected	41473
Independent reflections	41473[R_{int} = ?]
Completeness to ϑ = 25.242°	100.0 %
Max. and min. transmission	0.724 and 0.113
Data / restraints / parameters	41473 / 0 / 266
Goodness-of-fit on F^2	1.078
Final R indices [$I > 2\sigma(I)$]	R_1 = 0.0267, wR_2 = 0.0652
R indices (all data)	R_1 = 0.0395, wR_2 = 0.0688
Largest diff. peak and hole	2.950 and -1.297 e·Å^3
Crystallisation Details:	from MeOH / toluene, r.t.

Thermal ellipsoids are presented at the 50 % level of probability. Bond distances and bond angles are reported in Å or degree (°), respectively. N1–C1 1.337(3), N1–C2 1.388(3), Au1–Cl1 2.3192(6), Au1–C1 1.997(2), N2–C3 1.367(3), N2–C5 1.468(3), C2–C3 1.365(3), C2–C6 1.462(3), O1–C6 1.223(3), O2–C6 1.324(3), N1–C1–N2 107.70(17), C3–C2–N1 107.26(17), C1–N1–C2 108.65(17), C1–N1–C4 124.76(17), C1–N2–C3 109.70(18), O1–C6–O2 124.25(19), O2–C6–C2 111.99(18), N1–C1–Au1 126.48(15), C1–Au1–Cl1 176.95(6), Cl2–Au1–Cl1 93.17(2), Cl3–Au1–Cl2 174.44(2).

Table S8. Crystal data and structure refinement of **4b**.

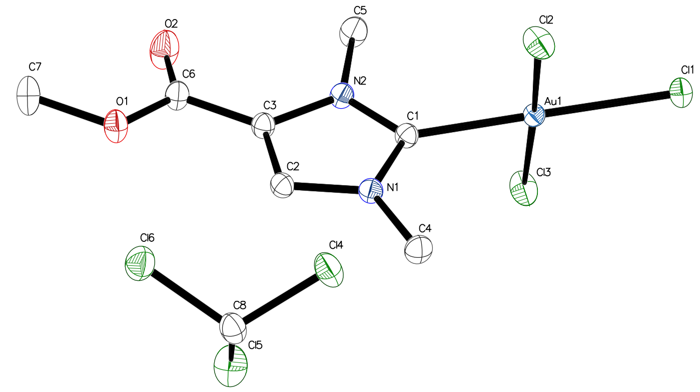

	CCDC	2355442
	Empirical formula	$C_8H_{11}AuCl_6N_2O_2$
	Formula weight	576.85
	Temperature	100(2) K
	Wavelength	0.71073 Å
	Crystal system	Monoclinic
	Space group	$P2_1/c$
	Unit cell dimensions	
	a = 7.9945(2) Å	α = 90°
	b = 10.9103(2) Å	β = 100.267(2)°
	c = 19.0105(4) Å	γ = 90°
Volume	1631.59(6) Å^3	
Z	4	
Density (calculated)	2.348 Mg/m^3	
Absorption coefficient	9.995 mm^{-1}	
F(000)	1080	
Crystal habitus	block (colourless)	
Crystal size	0.320 x 0.070 x 0.060 mm^3	
ϑ range for data collection	2.161 to 65.671°	
Index ranges	$-20 \leq h \leq 20$, $-26 \leq k \leq 27$, $-48 \leq l \leq 48$	
Reflections collected	289263	
Independent reflections	28759[R_{int} = 0.0626]	
Completeness to ϑ = 25.242°	99.9 %	
Max. and min. transmission	1.000 and 0.263	
Data / restraints / parameters	28759 / 0 / 175	
Goodness-of-fit on F^2	1.011	
Final *R* indices [$I > 2\sigma(I)$]	R_1 = 0.0357, wR_2 = 0.0750	
R indices (all data)	R_1 = 0.0640, wR_2 = 0.0842	
Largest diff. peak and hole	2.527 and -4.379 e·Å^3	
Crystallisation Details:	from $CHCl_3$ / *n*-Hexane (DCM), r.t.	

Thermal ellipsoids are presented at the 50 % level of probability. Bond distances and bond angles are reported in Å or degree (°), respectively. N1–C1 1.3478(18), N1–C2 1.3692(19), Au1–Cl1 2.3129(4), Au1–C1 2.0022(14), N2–C3 1.3903(18), N2–C5 1.465(2), C2–C3 1.363(2), C3–C6 1.472(2), O1–C6 1.331(2), O1–C7 1.451(2), O2–C6 1.207(2), N2–C1–N1 107.68(12), C3–C2–N1 107.06(12), C1–N1–C2 109.51(12), C1–N1–C4 125.78(13), C1–N2–C3 108.84(12), O2–C6–O1 125.16(15), C6–O1–C7 115.40(15), O2–C6–C3 124.87(15), N1–C1–Au1 125.84(10), C1–Au1–Cl1 178.89(4), C1–Au1–Cl2 89.70(4), Cl3–Au1–Cl2 177.747(15).

Table S9. Crystal data and structure refinement of **4c**.

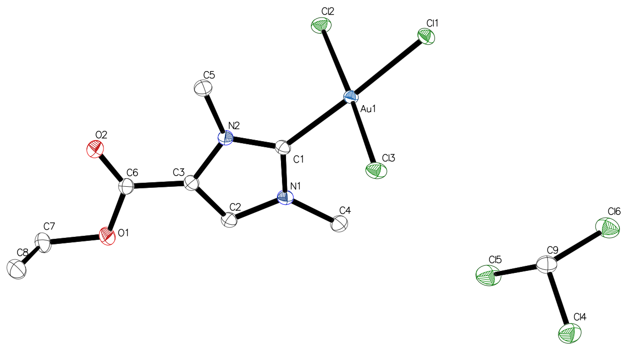

CCDC	2355443	
Empirical formula	$C_9H_{12}AuCl_6DN_2O_2$	
Formula weight	591.89	
Temperature	100(2) K	
Wavelength	0.71073 Å	
Crystal system	Monoclinic	
Space group	$P2_1/c$	
Unit cell dimensions		
	a = 8.2100(2) Å	α = 90°
	b = 11.0977(2) Å	β = 98.627(2)°
	c = 19.0738(5) Å	γ = 90°
Volume	1718.19(7) $Å^3$	
Z	4	
Density (calculated)	2.288 Mg/m^3	
Absorption coefficient	9.495 mm^{-1}	
F(000)	1112	
Crystal habitus	irregular (colourless)	
Crystal size	0.630 x 0.220 x 0.110 mm^3	
ϑ range for data collection	2.129 to 62.673°	
Index ranges	$-20 \leq h \leq 18$, $-27 \leq k \leq 27$, $-47 \leq l \leq 47$	
Reflections collected	293238	
Independent reflections	28103[R_{int} = 0.0897]	
Completeness to ϑ = 25.242°	99.8 %	
Max. and min. transmission	1.000 and 0.126	
Data / restraints / parameters	28103 / 0 / 185	
Goodness-of-fit on F^2	1.024	
Final R indices [$I > 2\sigma(I)$]	R_1 = 0.0344, wR_2 = 0.0614	
R indices (all data)	R_1 = 0.0601, wR_2 = 0.0676	
Largest diff. peak and hole	4.014 and -3.374 e·$Å^3$	
Crystallisation Details:	from $CDCl_3$ / n-pentane, r.t.	

Thermal ellipsoids are presented at the 50 % level of probability. Bond distances and bond angles are reported in Å or degree (°), respectively. N1–C1 1.3512(16), N1–C2 1.3718(17), Au1–Cl1 2.3165(3), Au1–C1 1.9997(12), N2–C3 1.3937(16), N2–C5 1.4652(17), C2–C3 1.3614(18), C3–C6 1.4707(18), O1–C6 1.3341(17), O1–C7 1.4546(19), O2–C6 1.2084(18), N2–C1–N1 107.49(11), C3–C2–N1 107.05(11), C1–N1–C2 109.62(11), C1–N1–C4 126.04(11), C1–N2–C3 108.81(10), O2–C6–O1 125.72(13), C6–O1–C7 115.94(12), O2–C6–C3 124.65(13), N1–C1–Au1 127.20(9), C1–Au1–Cl1 176.53(4), Cl2–Au1–Cl1 91.157(13), Cl3–Au1–Cl2 177.234(14).

Table S10. Crystal data and structure refinement of **5b**.

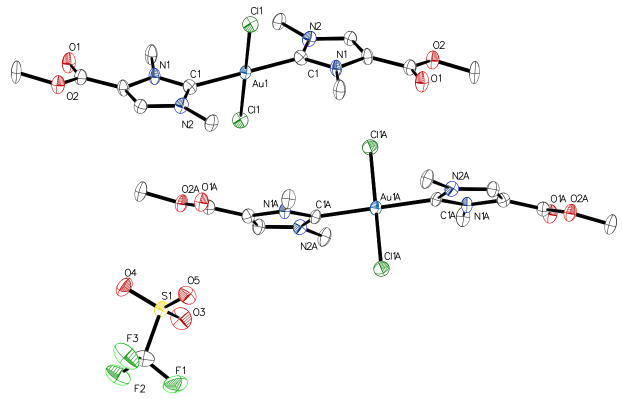	CCDC	2355444
	Empirical formula	$C_{15}H_{20}AuCl_2F_3N_4O_7S$
	Formula weight	725.28
	Temperature	100(2) K
	Wavelength	0.71073 Å
	Crystal system	Triclinic
	Space group	$P\bar{1}$
	Unit cell dimensions	
	a = 9.84290(10) Å	α = 82.8460(10)°
	b = 10.46050(10) Å	β = 85.0970(10)°
	c = 12.6806(2) Å	γ = 65.3480(10)°
Volume	1176.57(3) Å^3	
Z	2	
Density (calculated)	2.047 Mg/m^3	
Absorption coefficient	6.635 mm^{-1}	
F(000)	700	
Crystal habitus	block (colourless)	
Crystal size	0.200 x 0.080 x 0.060 mm^3	
ϑ range for data collection	2.153 to 32.585°	
Index ranges	$-14 \le h \le 14$, $-15 \le k \le 15$, $-19 \le l \le 19$	
Reflections collected	16519	
Independent reflections	16519[R_{int} = ?]	
Completeness to ϑ = 25.242°	99.9 %	
Max. and min. transmission	1.000 and 0.272	
Data / restraints / parameters	16519 / 0 / 308	
Goodness-of-fit on F^2	1.055	
Final *R* indices [$I > 2\sigma(I)$]	R_1 = 0.0718, wR_2 = 0.2010	
R indices (all data)	R_1 = 0.0889, wR_2 = 0.2122	
Largest diff. peak and hole	5.734 and -3.532 e·Å^3	
Crystallisation Details:	from $CDCl_3$, r.t.	

Thermal ellipsoids are presented at the 50 % level of probability. Bond distances and bond angles are reported in Å or degree (°), respectively. N1–C1 1.343(12), N1–C4 1.464(12), Au1–Cl1 2.036(9), Au1–Cl1 2.275(2), N2–C1 1.347(11), N2–C5 1.470(11), O1–C6 1.196(11), O2–C6 1.336(11), O2–C7 1.455(11), N1–C1–N2 107.4(8), C3–C2–N1 107.0(8), C1–N1–C2 108.7(8), C1–N1–C4 123.6(8), O1–C6–O2 125.3(9), C6–O2–C7 113.9(8), N1–C1–Au1 125.1(7), Cl1$^{\#1}$–Au1–Cl1 180.0, C1–Au1–C1$^{\#1}$ 180.0(5) (Symmetry transformations used to generate equivalent atoms: $^{\#1}$ -x+2,-y+2,-z+1).

Table S11. Crystal data and structure refinement of **5c**.

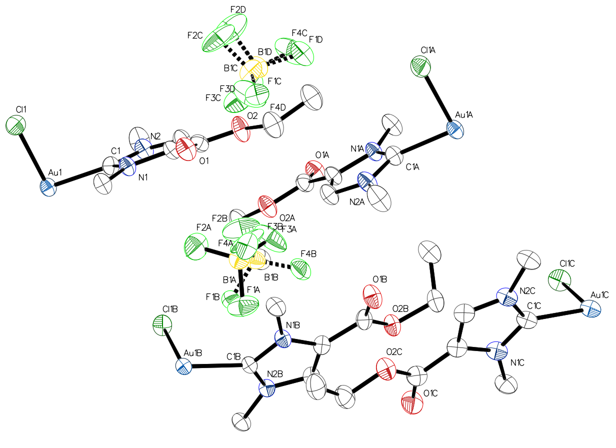	CCDC	2355445
	Empirical formula	$C_{16}H_{24}AuBCl_2F_4N_4O_4$
	Formula weight	691.07
	Temperature	100(2) K
	Wavelength	1.54184 Å
	Crystal system	Triclinic
	Space group	$P\bar{1}$
	Unit cell dimensions	
	a = 10.9098(4) Å	α = 104.139(2)°
	b = 11.4446(4) Å	β = 92.906(2)°
	c = 21.1235(4) Å	γ = 107.309(3)°
Volume	2419.94(14) $Å^3$	
Z	4	
Density (calculated)	1.897 Mg/m^3	
Absorption coefficient	14.013 mm^{-1}	
F(000)	1336	
Crystal habitus	plate (colourless)	
Crystal size	0.160 x 0.140 x 0.010 mm^3	
ϑ range for data collection	4.176 to 80.549°	
Index ranges	$-13 \le h \le 13$, $-14 \le k \le 12$, $-26 \le l \le 26$	
Reflections collected	104474	
Independent reflections	10403[R_{int} = 0.0775]	
Completeness to ϑ = 67.684°	100.0 %	
Max. and min. transmission	1.000 and 0.182	
Data / restraints / parameters	10403 / 254 / 688	
Goodness-of-fit on F^2	1.142	
Final R indices [$I > 2\sigma(I)$]	R_1 = 0.0371, wR_2 = 0.1176	
R indices (all data)	R_1 = 0.0396, wR_2 = 0.1200	
Largest diff. peak and hole	1.360 and -1.754 e·$Å^3$	
Crystallisation Details:	from CH_2Cl_2 / n-hexane, rt.	

Thermal ellipsoids are presented at the 50 % level of probability. Bond distances and bond angles are reported in Å or degree (°), respectively. N1–C1 1.343(4), N1–C4 1.468(4), Au1–C1 2.033(3), N2–C1 1.352(4), N2–C5 1.475(4), O1–C6 1.211(4), O2–C6 1.336(4), O2–C7 1.457(4), N1–C1–N2 106.8(3), C3–C2–N1 107.1(3), C1–N1–C2 109.0(3), C1–N1–C4 124.3(2), O1–C6–O2 125.0(3), C6–O2–C7 115.7(3), N1–C1–Au1 126.7(2), Cl1[#1]–Au1–Cl1 180.0, C1[#1]–Au1–C1 180.0 (Symmetry transformations used to generate equivalent atoms: [#1] -x+1,-y+2,-z+1).

S1.4. Stability Tests of Gold Complexes

The stability of the gold complexes **2a-c** to **5a-c** was investigated by ^{1}H NMR monitoring with respect to a conceivable Au-C bonds scission and the concomitant formation of protonated carbenes **1a-c**. A mixture of DMSO-d_6 and D_2O (9:1, w/w) was employed as an NMR solvent to simulate the conditions of the biological assays applied. ^{1}H NMR spectra of the pure ligand precursors **1a-c** displayed signals, which were significantly different from those of complexes **2a-c** to **5a-c**. All ^{1}H NMR samples did not show any diagnostic changes after 24 h. Additionally, ^{1}H NMR samples of equimolar mixture of ligands **1a-c** and complexes **2a-c** to **5a-c** gave rise to spectra, which resulted from the overlap of two independent species, i.e. ligand and respective complex.

<u>Example 1</u>

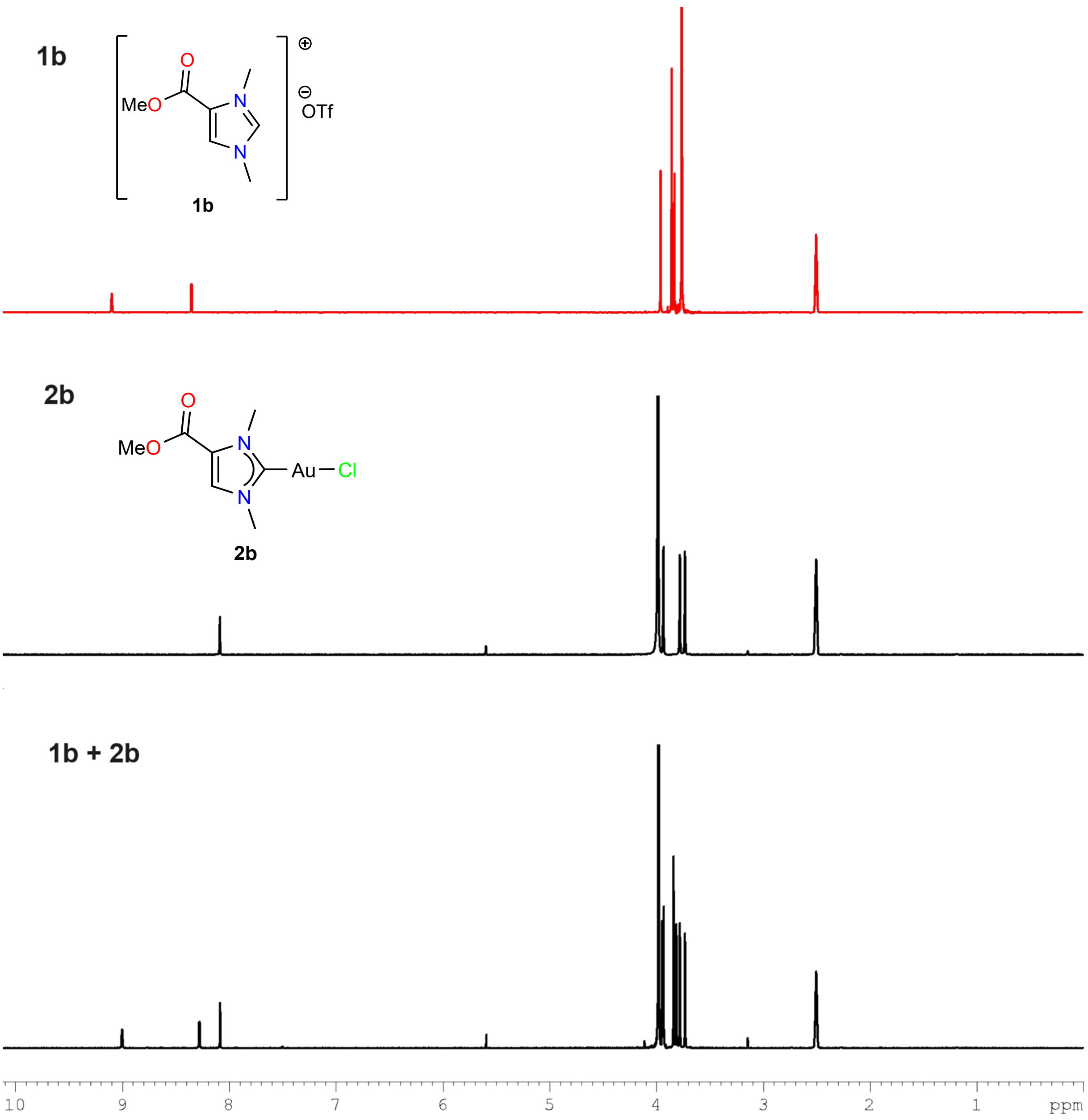

Figure S39. ^{1}H NMR spectra of **1b**, **2b** and an equimolar mixture of **1b** + **2b** at room temperature. All spectra were retained after 24 h without significant changes.

Example 2

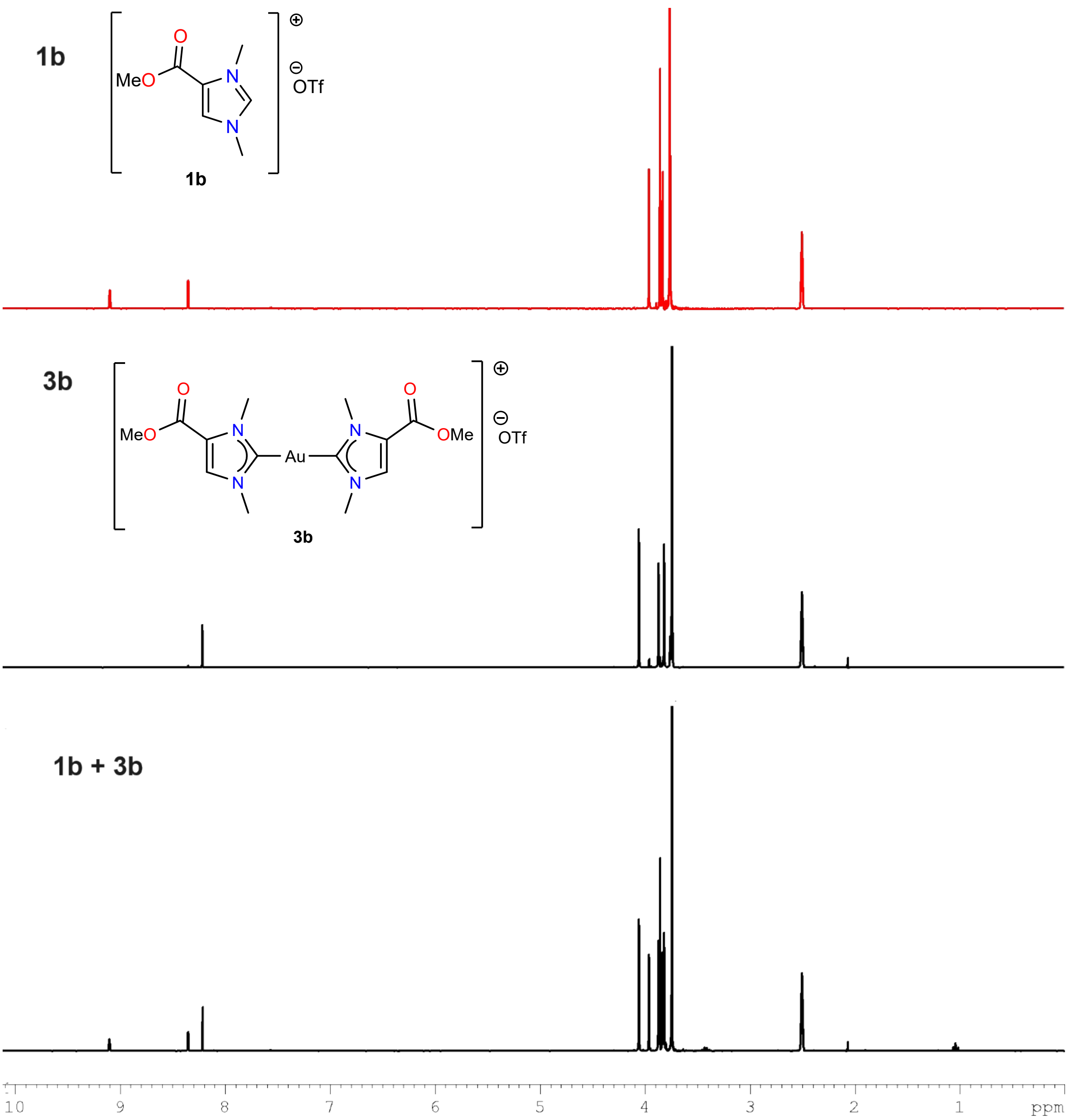

Figure S40. ^{1}H NMR spectra of **1b**, **3b** and an equimolar mixture of **1b** + **3b** at room temperature. All spectra were retained after 24 h without significant changes.

Example 3

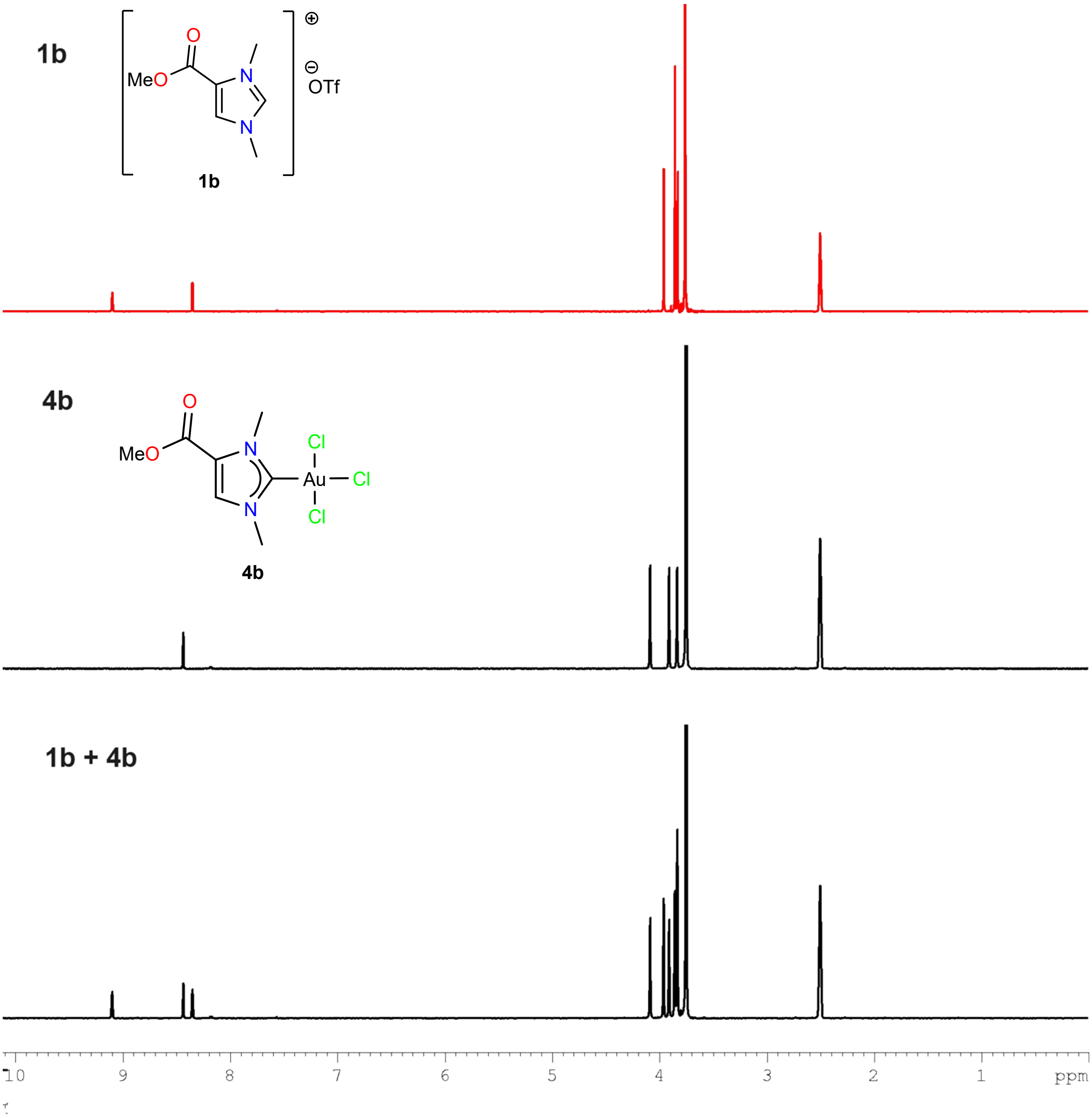

Figure S41. ^{1}H NMR spectra of **1b**, **4b** and an equimolar mixture of **1b** + **4b** at room temperature. All spectra were retained after 24 h without significant changes.

S2.1. Antibacterial Studies

Bacterial cultures were prepared by overnight aerobic incubation at 37 °C. Gram-negative strains were cultivated in Müller Hinton broth supplemented with 1% glucose at pH 7.2, while Gram-positive strains were grown in Trypticase soy yeast extract medium (TSY) with a composition of 30 g/l trypticase soy broth, 3 g/l yeast extract, and pH 7.2. The cultures were adjusted to an OD600 of 0.001, resulting in a final starting OD600 of 0.0005 for the subsequent tests. For testing, 25 µL of the prepared culture was combined with 25 µL of serially diluted test compounds in 384 well plates, following established protocols for different strains. Screening was performed at compound concentrations of 0.5, 5, and 50 µM, and for selected compounds, growth inhibition curves were recorded using DMSO stock solutions at final concentrations of 100, 50, 25, 12.5, 6,25, 3.125, 1.56, 0.78, 0.39, and 0.2 µM.
Positive control compounds, namely linezolid (for MRSA strains), ciprofloxacin (for *E. faecium* and *E. coli*), and amikacin (for *P. aeruginosa*), were included. The maximum DMSO concentration in the assay was 1%, which did not impact bacterial growth. After an 18-hour incubation at 37 °C under moist conditions, the optical density at 600 nm was measured using a Fusion Universal Microplate Analyser (Perkin–Elmer, Waltham, USA). The minimum inhibitory concentration (MIC) values, representing the lowest compound concentration that completely suppressed growth, were determined through curve fitting with Sigma Plot. The bacterial strains used included Gram-negative strains: *Escherichia coli* (DSM 1116), *A. baumannii* (DSM30007), *K. pneumoniae* (DSM111678), and *Pseudomonas aeruginosa* PA7 (DSM 24068); and Gram-positive strains: *E. faecium* (DSM20477) and *Staphylococcus aureus* MRSA (DSM 11822).

Table S12. Antibacterial activities of mono and dicarbene gold (I) and gold (III) complexes. Minimal inhibitory concentrations (MIC) are given in µM ± standard error of the mean. As positive control antibiotics, amikacin (P.a.), linezolid (MRSA) and ciprofloxacin (all other strains) have been used.

Compound	*E. faecium*	*MRSA*	*A. baumannii*	*E. coli*	*K. pneumoniae*	*P. aeruginosa*
**2a**	>100	>100	42.95±0	85.89±0	42.95±6.75	>100
**2b**	10.35±0	10.35±0	10.35±0	41.39±0	41.39±0	>100
**2c**	9.98±0	9.98±0	9.98±2.07	39.94±0	39.94±0	>100
**3a**	>100	>100	31.2±4.90	62.4±0	62.4±0	62.4±0
**3b**	>100	>100	>100	>100	>100	>100
**3c**	>100	25.79±0	>100	>100	>100	>100
**4a**	>100	>100	36.08±2.83	72.16±0	36.08±5.67	>100
**4b**	17.49±0	17.49±0	8.74±1.82	34.97±0	34.97±0	>100
**4c**	16.97±0	16.97±2.67	8.48±1.33	33.93±0	33.93±5.33	>100
**5a**	>100	>100	>100	>100	>100	>100
**5b**	>100	>100	>100	>100	>100	>100
**5c**	>100	23.15±0	>100	>100	>100	>100
**Auranofin**	0.3	0.4	55	46	81	>100
**Antibiotic**[b)]	9.5	4.7	1	0.1	0.2	7

S2.2. Cytotoxicity and Anticancer Studies

Cell culture and cytotoxicity

Cell lines were sourced from DSMZ (German Collection of Microorganisms and Cell Cultures GmbH) and maintained through standard procedures in Dulbecco's Modified Eagle Medium (DMEM) supplemented with 10% fetal calf serum and 50 mg/L gentamycin. Antiproliferative effects were assessed using a well-established assay protocol. In brief, 100 µL volumes of A549 cells (13,243 cells per mL), HT-29 cells (14,657 cells per mL), MDA-MB-231 cells (6,613 cells per mL), or MCF-7 cells (6,917 cells per mL) were transferred into the wells of 96-well plates and incubated at 37°C/5% CO_2 for 72 hours (MCF-7, MDA-MB-231) or 48 hours (A549, HT-29).

Freshly prepared stock solutions of the compounds in DMSO were diluted with the respective cell culture medium to achieve graded concentrations (final concentration of DMSO: 0.1% v/v). After 72 hours (A549, HT-29) or 96 hours (MCF-7, MDA-MB-231) of exposure, the cell biomass was assessed through crystal violet staining, and the IC_{50} value was determined as the concentration causing 50% inhibition of cell proliferation compared to an untreated control. Results were calculated as the mean values of three independent experiments.

S2.3. Inhibition of bacterial TrxR from *E. coli*

The TrxR (*E. coli*) inhibition assay was performed according to previously published procedures.[8] The assay is partly based on the procedure developed by Lu et al[9] and makes use of the reduction of DTNB (5,5'-dithiobis-(2-nitrobenzoic acid)). Solutions of *E. coli* TrxR (10.1 U/mL) and *E. coli* thioredoxin (Trx) (312 µg/mL) (both purchased from Sigma-Aldrich) were prepared in distilled water. Stock solutions of the test compounds (2 mM) were prepared in DMSO and serially diluted with TE buffer (Tris-HCl 50 mM, EDTA 1 mM, pH 7.5). 20 µL of these solutions or TE buffer without the test compounds (positive control) were mixed with the TrxR solution (10 µL), the Trx solution (10 µL) and 100 µl of NADPH (200 µM) in TE buffer in a well on a 96-well plate. As a blank control, 200 µM NADPH in TE buffer (100 µL) mixed with a DMSO / buffer mixture (40 µL) was used (final concentrations of DMSO: 0.5% v/v). The plate was incubated for 75 min at 25°C with moderate shaking. After incubation, 100 µL of a reaction mixture (TE buffer containing 200 µM NADPH and 5 mM DTNB) was added to each well to initiate the reaction. After thorough mixing, the formation of 5-TNB was monitored by a microplate reader at 405 nm in 35 s intervals (10 measurements). The values were corrected by subtraction of the blank solution absorption values. The increase in concentration of 5-TNB followed a linear trend ($r^2 \geq 0.990$) and the enzymatic activities were calculated as the gradients (increase in absorbance per second) thereof. Absence of interference with the assay components was confirmed by a negative control experiment for each test compound, where the highest test compound concentration was used and the enzyme solution was replaced by TE buffer. The inhibition is presented as the mean IC_{50} values and standard deviations obtained in three independent experiments.

Table S13. Activity of TrxR from *E. coli* in presence of compounds at 0.5 µM (n = 2, n.d.= not determined)

Compound	**2a**	**2b**	**2c**	**3a**	**3b**	**3c**	**4a**	**4b**	**4c**	**5a**	**5b**	**5c**
TrxR activity, % of control	94.6	8.9	13.8	n.d.	106	115	86.1	36.5	28.5	91.6	90.8	93.5

S2.4. Cellular uptake and metal quantification studies in A549 cells

Cellular uptake studies in A549 cells

For cellular accumulation studies, A549 lung carcinoma cells were seeded in 75 cm^2 cell culture flasks, incubated with 10 ml DMEM/10% FCS cell culture medium at 37 °C in a 5% CO_2 humidified atmosphere until they reached a confluence of 75-80%. The cells were treated with DMEM containing selected test compounds **3a** and **3c** dissolved in DMF (final DMF concentration: 0.1%; final test compound concentration: 2 µM). After 6 and 24 h of exposure, cells were isolated by removing the media followed by trypsinization. The trypsinized cells were washed with 9 ml PBS buffer (pH 7.4), scraped, and isolated by centrifugation (5 min, 3500 rpm). The obtained cell pellets were stored at -20 °C for further use. All experiments were carried out in triplicate.

Metal quantification in cell lysates by HRCS-AAS

Dry cell pellets were diluted with 400 µl of Milli-Q® water and lysed for 30 min in an ultrasonic bath (240 W, 65 Hz). For protein quantification. 20 µl of each sample was added to 200 µl of Bradford reagent (25 mg Serva Blue G (Sigma Aldrich) in 25 ml Ethanol 96%, 25 ml Milli-Q® water and 50 ml H_3PO_4 86%, stored at -20 °C and freshly diluted 1+4 with Milli-Q® water prior to use) and incubated on a 96-well plate while shaking at room temperature for 30 min and measured by a Perkin Elmer Victor X4 plate reader at a wavelength of 595 nm. The measurements were performed in duplicate, and the mean was used to calculate the protein content. For a standard calibration curve, bovine serum albumin (Sigma Aldrich) was used in graded concentrations.

The gold quantification was performed using a high-resolution continuum source atomic absorption spectrometer ContrAA® 700 (Analytik Jena AG). A pure sample of the respective complex **5c** was used as standard, and calibration was done in a matrix-matched manner (meaning all samples and standards were adjusted to a protein concentration of 1 mg/ml by dilution with Milli-Q® water). The calibration and samples were measured in duplicate. The final cellular gold concentrations were calculated from data obtained in three independent experiments and are expressed as pmol of metal per mg of cell protein. Sample preparation: Triton-X 100 (1%, 20 µl) and ascorbic acid (1%, 20 µl) were added to each calibration standard or probe (200 µl). A volume of 20 µl was injected into a coated standard graphite tube (Analytik Jena AG) and thermally processed as previously described.[10] The gold amount was analyzed at a wavelength of 242.79 nm.

S3.1. References

[1] M.-C. Brandys, M. C. Jennings, R. J. Puddephatt, *Journal of the Chemical Society, Dalton Transactions* **2000**, 4601.

[2] S. Mahdavi, D. Bockfeld, R. Büssing, B. Karge, T. Bannenberg, R. Frank, M. Brönstrup, I. Ott, M. Tamm, *Dalton Transactions* **2024**.

[3] Rigaku Oxford Diffraction, CrysAlisPRO Software System, versions 1.171.40.61a, 1.171.42.64a, and 1.171.42.101a (2021), Rigaku Corporation, Oxford, UK.

[4] G. M. Sheldrick, *Acta Crystallographica Section A: Foundations of Crystallography* **2008**, *64*, 112.

[5] G. M. Sheldrick, *Acta Crystallographica Section A: Foundations and Advances* **2015**, *71*, 3.

[6] O. V. Dolomanov, L. J. Bourhis, R. J. Gildea, J. A. K. Howard, H. Puschmann, *Journal of applied crystallography* **2009**, *42*, 339.

[7] A. L. Spek, *Journal of applied crystallography* **2003**, *36*, 7.

[8] C. Schmidt, B. Karge, R. Misgeld, A. Prokop, R. Franke, M. Brönstrup, I. Ott, *Chem. Eur. J.* **2017**, *23*, 1869.

[9] J. Lu, A. Vlamis-Gardikas, K. Kandasamy, R. Zhao, T. N. Gustafsson, L. Engstrand, S. Hoffner, L. Engman, A. Holmgren, *FASEB journal : official publication of the Federation of American Societies for Experimental Biology* **2013**, *27*, 1394.

[10] C. Schmidt, B. Karge, R. Misgeld, A. Prokop, R. Franke, M. Brönstrup, I. Ott, *Chem. Eur. J.* **2017**, *23*, 1869.
